# Understanding Cu(i) local environments in MOFs *via*^63/65^Cu NMR spectroscopy†

**DOI:** 10.1039/d4sc00782d

**Published:** 2024-02-27

**Authors:** Wanli Zhang, Bryan E. G. Lucier, Victor V. Terskikh, Shoushun Chen, Yining Huang

**Affiliations:** a Department of Chemistry, The University of Western Ontario 1151 Richmond Street London Ontario N6A 5B7 Canada yhuang@uwo.ca; b Metrology, National Research Council Canada Ottawa Ontario K1A 0R6 Canada; c College of Chemistry and Chemical Engineering, Lanzhou University Lanzhou 730000 China

## Abstract

The field of metal–organic frameworks (MOFs) includes a vast number of hybrid organic and inorganic porous materials with wide-ranging applications. In particular, the Cu(i) ion exhibits rich coordination chemistry in MOFs and can exist in two-, three-, and four-coordinate environments, which gives rise to many structural motifs and potential applications. Direct characterization of the structurally and chemically important Cu(i) local environments is essential for understanding the sources of specific MOF properties. For the first time, ^63/65^Cu solid-state NMR has been used to investigate a variety of Cu(i) sites and local coordination geometries in Cu MOFs. This approach is a sensitive probe of the local Cu environment, particularly when combined with density functional theory calculations. A wide range of structurally-dependent ^63/65^Cu NMR parameters have been observed, including ^65^Cu quadrupolar coupling constants ranging from 18.8 to 74.8 MHz. Using the data from this and prior studies, a correlation between Cu quadrupolar coupling constants, Cu coordination number, and local Cu coordination geometry has been established. Links between DFT-calculated and experimental Cu NMR parameters are also presented. Several case studies illustrate the feasibility of ^63/65^Cu NMR for investigating and resolving inequivalent Cu sites, monitoring MOF phase changes, interrogating the Cu oxidation number, and characterizing the product of a MOF chemical reaction involving Cu(ii) reduction to Cu(i). A convenient avenue to acquire accurate ^65^Cu NMR spectra and NMR parameters from Cu(i) MOFs at a widely accessible magnetic field of 9.4 T is described, with a demonstrated practical application for tracking Cu(i) coordination evolution during MOF anion exchange. This work showcases the power of ^63/65^Cu solid-state NMR spectroscopy and DFT calculations for molecular-level characterization of Cu(i) centers in MOFs, along with the potential of this protocol for investigating a wide variety of MOF structural changes and processes important for practical applications. This approach has broad applications for examining Cu(i) centers in other weight-dilute systems.

## Abstract 2

^63/65^Cu solid-state NMR spectroscopy provides a wealth of information regarding the local geometry and bonding at Cu(i) in metal–organic frameworks. This approach is also applicable when systems contain Cu(ii) centers or at lower magnetic fields.

## Graphical abstract

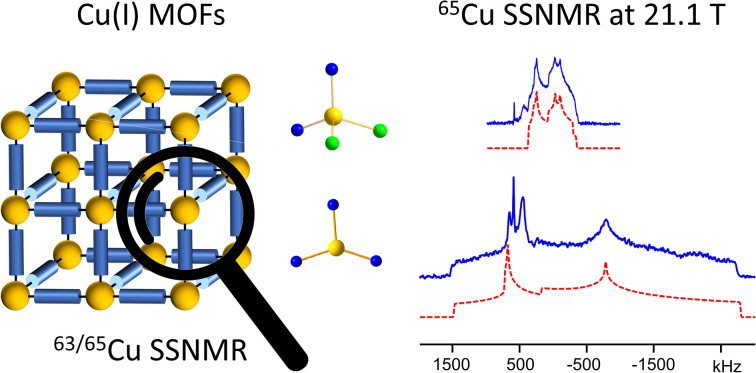

## Introduction

Metal–organic frameworks (MOFs) are porous crystalline materials composed of organic and inorganic components, arranged in a motif that features metal cations or metal–inorganic clusters connected by organic linkers.^1,2^ Due to their porosity, structural diversity, and functionality, these materials have shown promise for diverse applications in fields such as gas storage, gas separation, catalysis, sensing and drug delivery.^3–6^ The metal-centered entities are typically referred to as secondary building units (SBUs); the SBU composition and coordination can be tailored to achieve desired MOF topologies and properties.^7,8^

Copper(i) is a versatile metal that can adopt a multitude of coordination states; Cu(i) applications range from serving as active sites in catalysts to playing an integral role in proteins and biology. From a materials perspective, Cu(i) has the ability to form a wide variety of cluster-based compounds and MOFs.^9^ The copper(i) halide clusters Cu_*x*_X_*y*_ (X = Cl, Br, I) exhibit unique luminescent behaviors.^9^ Cu(i)-based MOFs have demonstrated catalytic activity in addition to luminescent properties.^10–12^ Cu(i) centers in MOFs can adopt three distinct local coordination geometries: two-coordinate linear, three-coordinate trigonal planar, and four-coordinate tetrahedral. Cu(i) can bind to a variety of different donor atoms on MOF linkers, including nitrogen, sulfur, phosphorus, and oxygen, and can form diverse one-, two-, and three-dimensional frameworks.^10,12–18^

Structural characterization is critical to understanding the molecular-level origins of unique MOF properties. The coordination state, geometry, local environment, and position of Cu(i) sites in the SBU and MOF influence the properties and applications of the resulting material. Cu(i) is generally regarded as a “spectroscopically silent” target that cannot be probed through traditional routes such as EPR and UV-vis spectroscopies, which makes characterization very challenging. Solid-state NMR spectroscopy can provide detailed information regarding local atomic environments in MOFs,^19–26^ including in cases of low sample crystallinity,^27,28^ short-range disorder,^29,30^ and framework defects.^31–33^ Many of the metal centers incorporated into MOFs are potential targets for NMR experiments.^20,34^^63/65^Cu solid-state NMR is one of the few spectroscopic techniques that can directly probe Cu(i) metal centers, and has previously been used to extract rich short-range data from simpler Cu(i) compounds.^35–50^^63/65^Cu NMR is subject to the anisotropic quadrupolar and chemical shift (CS) NMR interactions, and is thus a useful tool for understanding the three-dimensional local geometry and bonding around Cu(i) sites.^35,36,51–56^^63/65^Cu solid-state NMR is a promising untapped avenue for probing the local metal structure and unravelling structure–property relationships in Cu(i) MOFs.

Copper has two NMR active isotopes, ^63^Cu and ^65^Cu, which are both quadrupolar nuclei with a spin number (*I*) of 3/2. The electric quadrupolar moments (*Q*) of both nuclei are relatively high, where *Q*(^63^Cu) = −0.220 and *Q*(^65^Cu) = −0.204 barn.^57,58^ The natural abundance of ^63^Cu is 69.2% and ^65^Cu is 30.8%,^59^ yet ^65^Cu is generally the preferred option for solid-state NMR in systems where sensitivity is not an issue due to the smaller *Q* and higher gyromagnetic ratio (*γ*, where *γ*(^65^Cu) = 7.6104 × 10^7^ rad T^−1^ s^−1^ and *γ*(^63^Cu) = 7.1088 × 10^7^ rad T^−1^ s^−1^).^60^ In situations when the Cu(i) density within a material is low (*e.g.*, catalytic applications), the significantly more abundant ^63^Cu isotope may be a more prudent choice for NMR experiments. The sizeable *Q* of both isotopes renders ^63/65^Cu NMR spectra very broad when Cu does not reside in a local environment of high symmetry, making spectral acquisition challenging. The same anisotropic quadrupolar and chemical shift interactions that give rise to broadened and complicated ^63/65^Cu NMR spectra also encode a wealth of information regarding the local Cu environment.

The ^63/65^Cu NMR signals of many materials are broadened into the “ultra-wideline” frequency regime^61^ and are difficult to acquire, which has limited the use of ^63/65^Cu NMR for practical applications. Non-spinning (*i.e.*, static) experiments are well-suited for acquiring ultra-wideline ^63/65^Cu NMR spectra.^35,51^ Challenges associated with ^63/65^Cu NMR have been partially mitigated through the use of increasingly accessible high magnetic fields (*i.e.*, >18.8 T).^19,35,53,54^ The second order quadrupolar interaction (QI) that broadens central transition (+1/2 ↔ −1/2) ^63/65^Cu NMR spectra is inversely proportional to the magnetic field strength, which results in narrower signals at higher fields. Higher magnetic fields also enhance the population difference between the +1/2 and −1/2 spin states, increasing NMR sensitivity. While ^1^H–^63^Cu RESPDOR NMR experiments have been used to examine a Cu(i) MOF,^62^ there have been no reports regarding direct Cu(i) NMR of MOFs.

In this work, we report a ^63/65^Cu NMR study of Cu(i) MOFs featuring copper sites in various two-, three- and four-coordinate environments. The ^63/65^Cu NMR parameters quantified from 21.1 T data reveal key information regarding local symmetry and coordination about Cu. We use data from this work and prior studies to illustrate how the Cu quadrupolar coupling constant (*C*_Q_) values are highly dependent on the coordination number and geometric configuration of Cu(i) in MOFs, and present a general scale to guide researchers in determining the Cu(i) coordination number from *C*_Q_(Cu) values in MOFs and many other compounds. This experimental approach can be employed to monitor the structural evolution of MOFs, such as phase transitions, *via* effects on Cu(i) local environments. In favorable situations, the resolution of ultra-wideline ^63/65^Cu solid-state NMR spectra is sufficient to resolve signals from multiple Cu(i) sites.^35^ Practical applications of ^63/65^Cu NMR are explored with experiments on a Cu(i)/Cu(ii) mixed valence MOF featuring paramagnetic metal centers that lacks single crystal X-ray diffraction (XRD) data. A comprehensive examination of density functional theory (DFT) calculations and associated geometry optimizations have been performed to better understand the structural origins of experimental electric field gradient (EFG) tensors, along with any discrepancies between calculated and experimental NMR parameters. To finish, we show that ^65^Cu solid-state NMR spectra of Cu(i) MOFs can be successfully acquired at a more accessible lower magnetic field of 9.4 T with sufficient resolution to accurately extract Cu NMR parameters. The practical applications of this concept are illustrated by using ^65^Cu NMR at 9.4 T to elucidate local structural transformations associated with anion exchange in Cu MOFs. The ^63/65^Cu solid-state NMR approach in this work demonstrates a promising investigative route for the characterization of Cu(i)-based MOFs and their derivative materials, whether the crystal structure is known or unknown. The MOFs involved in this work are [CuCl(bpy)], [CuI(bpy)], [Cu_2_I_2_(bpy)], [Cu_2_Cl_2_(bpy)], [Cu_2_I_2_(pyz)], [Cu_4_I_4_(DABCO)_2_], {[CuI][Cu(pdc)(H_2_O)]·1.5MeCN·H_2_O}_*n*_, Cu_2_BDC, Cu(bpy)_1.5_NO_3_·1.25H_2_O, Cu_3_(4hypymca)_3_, SLUG-22, [Cu_6_I_6_(DABCO)_2_], and Cu_2_(pyz)_2_(SO_4_)(H_2_O)_2_, with additional details provided in Table S1.†

## Results and discussion

### MOFs with four-coordinate Cu(i) sites

#### Representative ^63/65^Cu NMR of tetrahedral Cu(i) sites in MOFs: [CuCl(bpy)] and [CuI(bpy)]

**Fig. 1:**
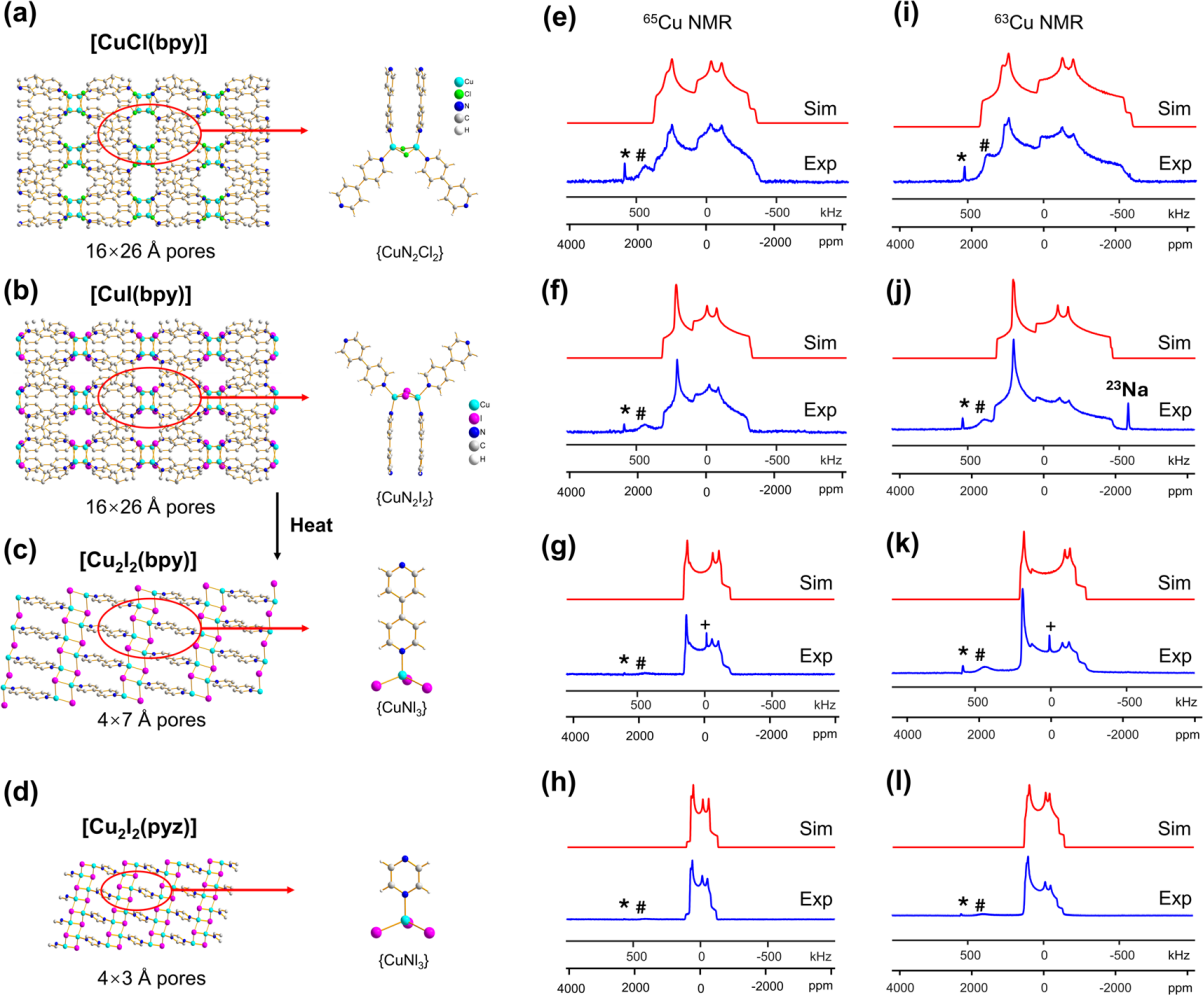
(a–d) A schematic illustration of the local and long-range structure in [CuCl(bpy)], [CuI(bpy)], [Cu_2_I_2_(bpy)], and [Cu_2_I_2_(pyz)]. The MOF pore size is listed below each structure. In (e–h), the experimental (“Exp.,” blue) and simulated (“Sim.,” red) ^65^Cu static NMR spectra of [CuCl(bpy)], [CuI(bpy)], [Cu_2_I_2_(bpy)] and [Cu_2_I_2_(pyz)] at 21.1 T are shown, with the corresponding ^63^Cu NMR spectra and simulations in (i–l). The asterisk (*) denotes a signal from metallic copper (Cu^0^) and the pound (#) marks a signal from probe background. The plus symbol (+) marks a resonance arising from residual CuI at 0 ppm after thermal treatment. A background ^23^Na signal is also noted in (j). The definitions of *, #, and + also apply to all other figures in this work.

The most common bonding configuration for Cu(i) in MOFs is in a four-coordinate tetrahedral or distorted tetrahedral fashion. The Cu-X-bpy series of MOFs (X = Cl, Br, I, bpy = 4,4′-bipyridine) have exhibited potential applications in photocatalytic hydrogen production.^10^ [CuCl(bpy)] is a neutral three-dimensional framework with open pores measuring *ca.* 2 × 4 Å. This compound crystallizes in the *I*4_1_/*acd* space group (Fig. 1(a)).^16^ Cu(i) resides in a slightly distorted tetrahedral environment in which adjacent Cu(i) centers are bound to two 4,4′-bpy ligands and bridged by two *μ*_2_-Cl atoms (Fig. 1(a)). The ^63/65^Cu static NMR spectra of [CuCl(bpy)] at 21.1 T are shown in Fig. 1(e and i). Both the ^63^Cu and ^65^Cu NMR spectra exhibit typical QI-dominated powder patterns that can be simulated using a single signal arising from one unique Cu site, which is consistent with the XRD structure.^16^ The metallic Cu(0) signal denoted by an asterisk (*) originates from the NMR probe, rather than the sample (Fig. S3†). There is also an additional check on the *C*_Q_ obtained from simulating the spectra at 21.1 T; the condition *C*_Q_(^63^Cu)/*C*_Q_(^65^Cu) = 1.078 must be satisfied, owing to the ratio between the respective nuclear *Q* values. Despite the dominance of the QI, there are fine features in the ^63/65^Cu NMR spectra that cannot be simulated using only quadrupolar parameters, which unambiguously confirms that Cu chemical shift anisotropy (CSA) must be present. The ^63/65^Cu NMR parameters (Table 1) were determined to be *C*_Q_(^65^Cu) = 30.0(4) MHz, *η*_Q_ = 0.45(3), *δ*_iso_ = 500(50) ppm, *Ω* = 600(200) ppm, *κ* = 0.4(1), *α* = 10(3)°, *β* = 28(3)°, and *γ* = 35(3)°; see the ESI† for descriptions of the NMR parameters.

**Table 1:** Experimental and calculated^a^^63/65^Cu NMR parameters

Site	Method^b^^,^^c^	*C*_Q_(^65^Cu)^d^ (MHz)	*C*_Q_(^63^Cu)^d^ (MHz)	*η*_Q_	*δ*_iso_ (ppm)	*Ω* (ppm)	*κ*	*α* (°)	*β* (°)	*γ* (°)
**[CuCl(bpy)]**										
Cu1	Exp.	30.0(4)	33.5(4)	0.45(3)	500(50)	600(200)	0.4(1)	10(3)	28(3)	35(3)
Cu1	Calc.	34.1	36.8	0.41	2188.7	2402.7	−0.24	27.3	60.4	−134.0

**[CuI(bpy)]**										
Cu1	Exp.	28.7(3)	30.2(4)	0.50(4)	400(50)	300(200)	1.0(1)	90(2)	35(2)	10(2)
Cu1	Calc.	28.6	30.8	0.45	1215.0	806.2	−0.24	34.3	45.0	−172.4

**[Cu**_**2**_ **I**_**2**_ **(bpy)]**										
Cu1	Exp.	24.0(4)	26.0(5)	0.18(3)	280(50)	400(200)	−1.0(3)	0(3)	25(2)	65(5)
Cu1	Calc.	25.5	27.5	0.47	867.3	741.4	0.15	23.4	20.0	−57.2

**[Cu**_**2**_ **Cl**_**2**_ **(bpy)]**										
Cu1	Exp.	30.0(3)	32.0(5)	0.25(2)	230(30)	500(100)	0.1(2)	0(2)	25(3)	58(2)
Cu1	Calc.	27.6	29.4	0.80	1070.0	906.5	−0.67	103.1	89.5	92.8

**[Cu**_**2**_ **I**_**2**_ **(pyz)]**										
Cu1	Exp.	18.8(4)	19.6(5)	0.35(2)	300(50)	480(50)	−0.8(2)	10(3)	25(2)	60(4)
Cu1	Calc.	18.2	19.6	0.53	3701.7	2585.8	−0.53	−53.5	4.6	43.5

**[Cu**_**4**_ **I**_**4**_ **(DABCO)**_**2**_ **]**										
Cu1	Exp.	22.1(5)	23.8(3)	0.09(3)	320(40)	250(75)	1.0(4)	0	0	0
Cu1	Calc.	16.4	17.7	0.22	−58.33	335.57	−0.56	−90	12.5	−180
Cu2	Exp.	20.6(3)	22.0(4)	0.14(4)	280(20)	280(50)	1.0(3)	0	0	0
Cu2	Calc.	15.3	16.5	0.37	−90.4	589.8	0.17	7.4	85.8	−160.5
Cu3	Exp.	26.7(6)	29.1(5)	0.03(3)	320(40)	200(50)	1.0(3)	0	0	0
Cu3	Calc.	23.0	24.8	0.02	−58.8	265.7	−0.87	90.0	5.4	−90.0

**{[CuI][Cu(pdc)(H**_**2**_ **O)]·1.5MeCN·H**_**2**_ **O}**_***n***_										
Cu1	Exp.	22.0(3)	24.0(2)	0.02(2)	400(15)	150(200)	1.0(4)	0	0	0
Cu1	Calc.	23.2	25.0	0.02	970.0	441.2	−0.87	0	15.9	90

**Cu**_**2**_ **BDC**										
Cu1	Exp.	53.0(3)	57.0(4)	0.22(3)	200(150)	1800(300)	1.0(4)	0	0	0
Cu1	Calc.	57.5	62.0	0.17	3713.6	6569.0	0.24	158.7	2.2	25.4

**Cu(bpy)**_**1.5**_ **NO**_**3**_ **·1.25H**_**2**_ **O**										
Cu1	Exp.	74.0(4)	79.0(6)	0.18(2)	300(100)	0	0	0	0	0
Cu1	Calc.	74.5	80.3	0.17	1337.3	2229.1	0.16	−40.8	1.3	39.0
Cu2	Exp.	55.2(8)	58.5(4)	0.00(0)	1300(200)	0	0	0	0	0
Cu3	Exp.	0	0	0	700(100)	0	0	0	0	0

**Cu**_**3**_ **(4hypymca)**_**3**_										
Cu1	Exp.	74.8(6)	80.6(4)	0.55(2)	150(200)	0	0	0	0	0
Cu1	Calc.	95.6	103.1	0.11	786.4	744.3	0.16	98.5	180.0	−136.0

**SLUG-22**										
Cu1,2	Exp.	63.0(1.0)	67.0(8)	0.34(2)	100(150)	1500(200)	1.0(1)	0	0	0
Cu1	Calc.	40.0	44.2	0.74	786.5	3181.5	−0.24	174.4	172.4	53.0
Cu2	Calc.	42.2	45.5	0.86	859.8	3055.1	−0.24	−17.3	3.17	−22.3

**[Cu**_**6**_ **I**_**6**_ **(DABCO)**_**2**_ **]**										
Cu1,2,3	Exp.	19.1(3)	21.4(3)	0.70(2)	670(20)	0	0	0	0	0
Cu1	Calc.	19.2	20.7	0.54	81.27	709.4	0.45	−28.6	125.2	175.7
Cu2	Calc.	19.3	20.8	0.25	124.5	724.9	−0.18	96.3	21.1	−143.4
Cu3	Calc.	7.2	7.8	0.34	324.5	704.7	0.53	0	104.3	0
Cu4	Exp.	24.1(2)	27.0(2)	0.20(3)	280(50)	0	0	0	0	0
Cu4	Calc.	24.1	26.0	0.24	257.0	868.6	−0.90	−90	0.22	90

**Cu**_**2**_ **(pyz)**_**2**_ **(SO**_**4**_ **)(H**_**2**_ **O)**_**2**_										
Cu1	Exp.	25.2(2)	27.2(4)	0.54(2)	500(100)	900(100)	0.0	70(2)	−4(2)	−11(3)
Cu1	Calc.	23.7	25.5	0.55	2079.4	2995	−0.39	43.5	58.0	−73.0

a Differences between the experimental and calculated values for both the CS tensor parameters and Euler angles are considerable due to the computational difficulties involved with calculating Cu CS tensor parameters.

b The “Exp.” label denotes experimental Cu NMR parameters obtained from best-fit simulations of ^65/63^Cu NMR spectra acquired at 21.1 T.

c The “Calc.” label denotes the NMR parameters obtained from plane-wave DFT calculations using the CASTEP software package. A geometry optimization of all atoms in the reported crystal structure was performed before calculation of NMR parameters; see the Materials and Methods section for additional details. Please see Table S5 for additional calculations performed using defined cluster models.

d The ^65/63^Cu NMR spectra were simulated independently. The experimental *C*_Q_(^63^Cu)/*C*_Q_(^65^Cu) ratio of 1.080 was found to be very close to the accepted quadrupole moment ratio *Q*(^63^Cu)/*Q*(^65^Cu) of 1.078,^57^ which gives additional confidence to the simulated fits.

The [CuI(bpy)] MOF (Fig. 1(b)) has a topology and local coordination of Cu(i) ions similar to that in [CuCl(bpy)]. The main difference between these compounds is that the Cu–I bond length in [CuI(bpy)] is *ca.* 0.2 Å longer than the Cu–Cl distance in [CuCl(bpy)]. The Cu NMR powder pattern (Fig. 1(f and j)) could be simulated using one unique Cu site (Table 1). [CuI(bpy)] has a lower *C*_Q_ and slightly higher *η*_Q_ (*C*_Q_(^65^Cu) = 28.7(3) MHz, *η*_Q_ = 0.50(4)) than [CuCl(bpy)], partially due to the more ionic nature of the Cu–I bond, illustrating the sensitivity of Cu NMR to local structure.

#### ^63/65^Cu solid-state NMR for detecting phase transitions in MOFs: [CuI(bpy)] and [Cu_2_I_2_(bpy)]

MOFs may undergo a phase transition upon external stimuli, such as exposure to different temperatures and pressures. A typical approach for monitoring long-range structural effects of MOF phase changes is powder XRD (PXRD); however, PXRD cannot intimately probe short-range structural variations, whereas solid-state NMR offers much more information regarding the local metal structure in MOFs.^63,64^ With this in mind, the ability of ^63/65^Cu NMR to investigate phase transitions in MOFs was explored using [CuI(bpy)].

The three-dimensional porous [CuI(bpy)] MOF is transformed to two-dimensional [Cu_2_I_2_(bpy)] with heat (Fig. 1(b, c) and S4†).^65^ [CuI(bpy)] crystallizes in the *I*4_1_/*acd* space group. The single unique Cu(i) center resides in a CuN_2_I_2_ slightly distorted tetrahedral local environment, which involves bonding to two N atoms from separate 4,4′-bpy ligands along with two bridging *μ*_2_-I ligands. In contrast, [Cu_2_I_2_(bpy)] is a two-dimensional layered material that crystallizes in the *P*1̄ space group with layer stacking along the crystallographic *b* axis. In [Cu_2_I_2_(bpy)], there is one Cu(i) site in a CuNI_3_ distorted tetrahedral environment, which is bound to three *μ*_3_-I species and one nitrogen atom from the 4,4′-bpy ligands. ^63/65^Cu solid-state NMR experiments were performed to investigate the local structure at Cu in both [CuI(bpy)] and the [Cu_2_I_2_(bpy)] product from thermal treatment (Fig. 1(b and c)). These MOFs give rise to well-defined ^63/65^Cu NMR powder patterns, which are dominated by the QI but also influenced by CSA, and are both indicative of one unique Cu site. The NMR spectra of [CuI(bpy)] and [Cu_2_I_2_(bpy)] are visually distinct and yield different ^63/65^Cu NMR parameters (Table 1). The *C*_Q_(^65^Cu) value of 28.7(3) MHz in [CuI(bpy)] is reduced to 24.0(4) MHz in [Cu_2_I_2_(bpy)], with the increased symmetry at Cu attributed to the change from a CuN_2_I_2_ to a CuNI_3_ local environment. The *η*_Q_ parameter is also sensitive to the phase change, falling from 0.50(4) in [CuI(bpy)] to 0.18(3) in [Cu_2_I_2_(bpy)], which is indicative of increased axial symmetry about the Cu center in [Cu_2_I_2_(bpy)].

In a manner similar to [CuI(bpy)], the three-dimensional [CuCl(bpy)] MOF can also undergo a transformation to the two-dimensional [Cu_2_Cl_2_(bpy)] MOF upon thermal treatment. The ^63/65^Cu NMR spectra of these two MOFs (Fig. S5†) are distinct and diagnostic of the phase change. While the *C*_Q_(Cu) values are very similar in both forms, *η*_Q_ changes from 0.45(3) in [CuCl(bpy)] to 0.25(2) in [Cu_2_Cl_2_(bpy)] (Table 1), producing a clear spectral difference indicative of a significant increase in local axial symmetry. The results indicate that ^63/65^Cu NMR is a viable spectroscopic route for tracking phase changes in Cu MOFs.

#### ^63/65^Cu solid-state NMR in reticular MOFs: [Cu_2_I_2_(bpy)] and [Cu_2_I_2_(pyz)]

The reticular synthesis of MOFs from specifically selected metal centers and organic linkers is an active field of research, since the pore size and eventual material properties can be controlled to a significant degree. Adjustment of the linker length while retaining key binding functional groups has proven an effective avenue to modify the pore size and specific surface area without changing the MOF topology.^66^ In the previous case, ^63/65^Cu NMR was used to investigate [Cu_2_I_2_(bpy)]. If the bpy linker is substituted with pyrazine (pyz) during synthesis, the [Cu_2_I_2_(pyz)] MOF is obtained. The shorter pyz linker means that [Cu_2_I_2_(pyz)] has a pore size of *ca*. 4 × 3 Å, while the longer bpy ligand translates to a larger *ca*. 4 × 7 Å aperture in [Cu_2_I_2_(bpy)]. The [Cu_2_I_2_(pyz)] MOF crystallizes in triclinic symmetry (space group *P*1̄) and has one unique Cu(i) site residing in a distorted tetrahedral CuNI_3_ environment. While the Cu(i) local bonding geometries are similar between [Cu_2_I_2_(bpy)] and [Cu_2_I_2_(pyz)],^65,67^ the ^63/65^Cu NMR powder patterns are relatively narrower in [Cu_2_I_2_(pyz)] (Fig. 1), and *C*_Q_(^65^Cu) falls from 24.0(4) MHz in [Cu_2_I_2_(bpy)] to 18.8(4) MHz in [Cu_2_I_2_(pyz)]. One reason for the decrease in *C*_Q_ is the smaller bond angle and bond length distributions involving Cu, while another possibility lies in long-range influences on the EFG that originate beyond the first coordination sphere of Cu (*i.e.*, the effect of different N-bound linker groups). A more detailed discussion can be found in the ESI.†

#### ^63/65^Cu solid-state NMR for resolving inequivalent Cu(i) sites in MOFs: [Cu_4_I_4_(DABCO)_2_]

In many MOFs there are multiple unique metal sites, which can often be challenging to characterize and distinguish using NMR techniques. Higher-resolution solid-state NMR techniques (*e.g.*, MAS, MQMAS) are not applicable due to the large quadrupolar interactions and broad lineshapes in ^63/65^Cu solid-state NMR; however, wideline NMR experiments on nuclei such as ^35^Cl have demonstrated that it is possible to distinguish multiple inequivalent sites.^68,69^ In this section, we show that ^63/65^Cu NMR can resolve separate resonances arising from several crystallographically inequivalent Cu sites in MOFs.

**Fig. 2:**
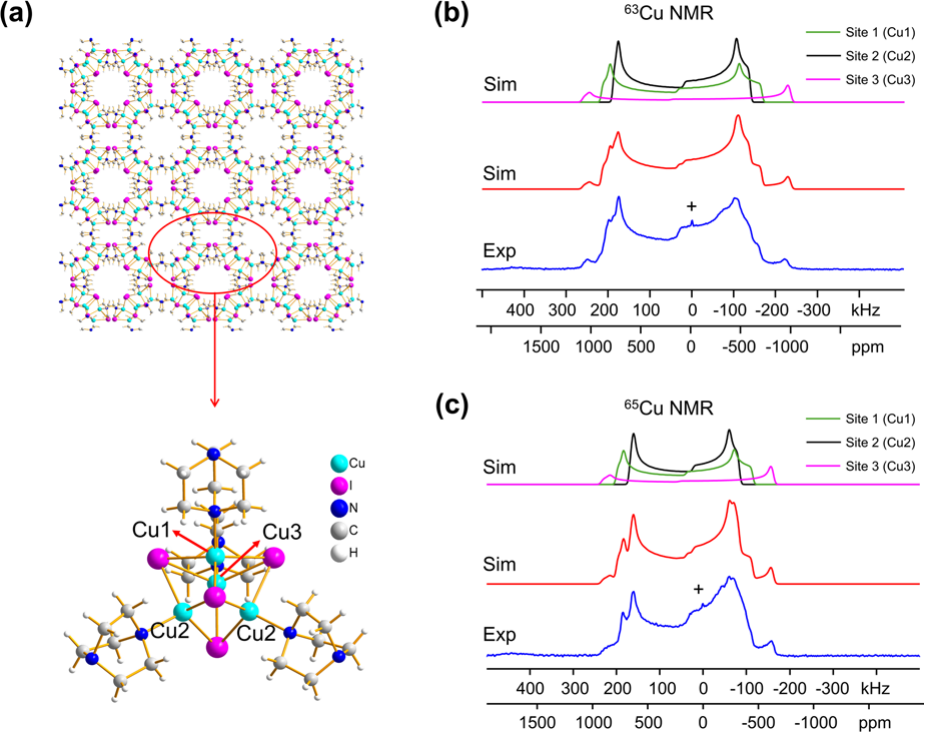
A schematic illustration of the long-range structure of [Cu_4_I_4_(DABCO)_2_] along with the local structure about Cu is shown in (a). The experimental (b) ^63^Cu and (c) ^65^Cu static NMR spectra (blue), cumulative simulations (red), and individual Cu site simulations (black, purple, green) of [Cu_4_I_4_(DABCO)_2_] at 21.1 T are also included.

Many MOFs containing Cu_*x*_I_*y*_ clusters feature multiple unique Cu(i) sites and have luminescent properties. The luminescent [Cu_4_I_4_(DABCO)_2_] MOF is composed of Cu_4_I_4_ clusters along with 1,4-diazabicyclo[2.2.2]octane (DABCO) linkers (Fig. 2(a)).^12^ This material crystallizes in the *P*_4_/*mcc* space group and has three inequivalent Cu(i) sites in the Cu_4_I_4_ unit, where the Cu sites are populated in the ratio Cu1 : Cu2 : Cu3 = 1 : 2 : 1. Each inequivalent Cu(i) site resides in a CuNI_3_ distorted tetrahedral environment, with the three coordinated iodine atoms originating from the Cu_4_I_4_ cluster and the nitrogen atom from a DABCO linker. The ^63/65^Cu NMR spectra (Fig. 2(b and c)) are both >400 kHz broad at 21.1 T, with fine features that hint at overlapping Cu resonances. Simulations of experimental data confirm that there are two narrower and overlapping Cu signals of higher intensity nested within a less intense, broader underlying signal; the NMR parameters obtained from simulations are summarized in Table 1.

With the NMR parameters successfully extracted, plane-wave DFT calculations were performed to assign ^63/65^Cu resonances to crystallographic sites. The calculated ^63/65^Cu NMR parameters (Table 1) indicate that Cu sites 1 and 2 should exhibit similar NMR parameters, including relatively smaller *C*_Q_(^63/65^Cu) values, while site 3 should correspond to unique NMR parameters and a larger *C*_Q_(^63/65^Cu). Accordingly, the two narrower components of the spectrum were assigned to Cu sites 1 and 2, with the broader signal corresponding to site 3. Given the similarities in *C*_Q_ values between Cu sites 1 and 2, an alternate NMR parameter, such as *η*_Q_, must be used to distinguish between them. A careful examination of the left quadrupolar “horn” of the two narrower signals located between +150 and +250 kHz in the ^63^Cu NMR spectra reveals significant detail, which differentiates the Cu1 and Cu2 powder patterns based on *η*_Q_ values. A comparison of local structural parameters between Cu2 and Cu1 shows that Cu2 has both the larger Cu–I bond length distribution and ∠N–Cu–I distribution of all Cu sites;^12^ this combination reflects a relatively lower axial symmetry in the Cu2 local environment and should result in a relatively higher *η*_Q_ value. Cu2 is thus assigned to the signal with *η*_Q_ = 0.14(4) and Cu1 is assigned to the signal with a smaller *η*_Q_ of 0.09(3). This assignment is also consistent with DFT calculations (Table 1, where *η*_Q_(Cu2) > *η*_Q_(Cu1) > *η*_Q_(Cu3)). For a more detailed discussion, please see the ESI.†

#### ^63/65^Cu NMR of mixed valence Cu(i/ii) MOFs: {[Cu(i)][Cu(ii)(pdc)(H_2_O)]·1.5MeCN·H_2_O}_*n*_ and Cu_2_BDC

**Fig. 3:**
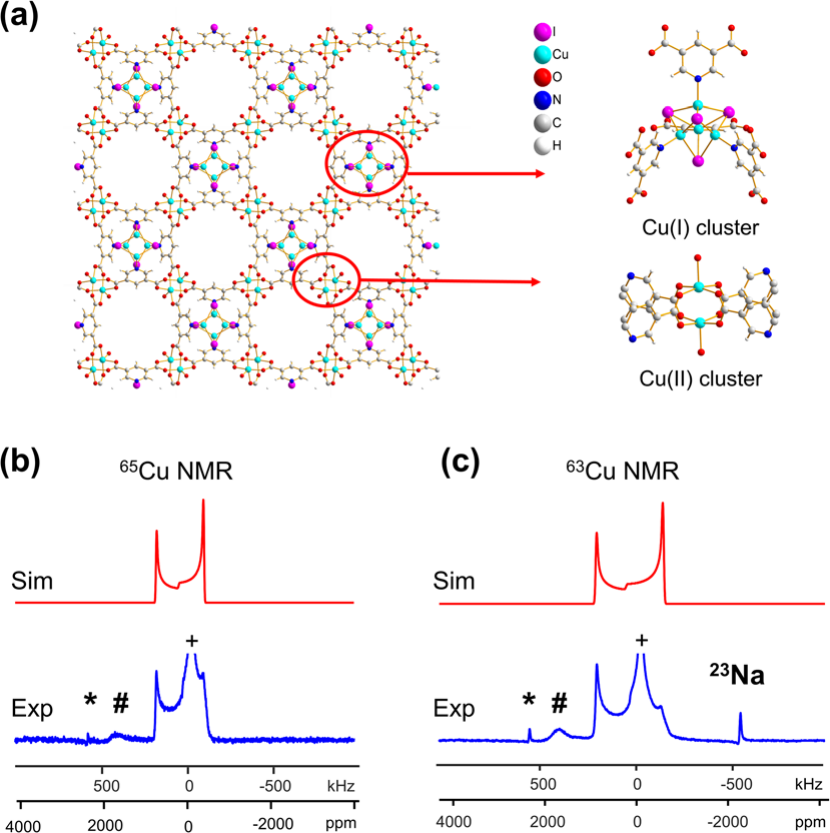
(a) A schematic illustration of the long-range and local structure in the {[Cu(i)][Cu(ii)(pdc)(H_2_O)]·1.5MeCN·H_2_O}_*n*_ MOF, including the Cu(i) and Cu(ii) clusters. The blue experimental and red simulated (b) ^65^Cu and (c) ^63^Cu static NMR spectra at 21.1 T are also shown.

There is a distinct family of mixed-valence MOFs that incorporate both Cu(i) and Cu(ii) metal centers, which have a variety of diverse structures, unique electronic properties, and catalytic applications.^70,71^ The mixed valence {[Cu(i)][Cu(ii)(pdc)(H_2_O)]·1.5MeCN·H_2_O}_*n*_ (where pdc = pyridine-3,5-dicarboxylic acid) MOF was selected as a test compound to investigate if ^63/65^Cu NMR could be used to probe materials containing both Cu(i) and Cu(ii).^72^ The pdc linker contains both nitrogen and carboxylate groups, which can form MOFs with two separate types of metal nodes upon reaction with CuI. The {[Cu(i)][Cu(ii)(pdc)(H_2_O)]·1.5MeCN·H_2_O}_*n*_ MOF features a paddlewheel-type local structure incorporating Cu(ii) centers, along with a Cu_4_I_4_ cluster containing Cu(i) (Fig. 3(a)). Cu 2p_3/2_ XPS spectra (Fig. S7(a)†) confirmed that both Cu(i) and Cu(ii) centers were present in the sample. The single crystal XRD structure of {[Cu(i)][Cu(ii)(pdc)(H_2_O)]·1.5MeCN·H_2_O}_*n*_^72^ features Cu(i) sites in CuNI_3_ distorted tetrahedral environments. The ^63/65^Cu NMR spectra (Fig. 3(b and c)) were simulated using one Cu signal with a small amount of CSA (Fig. S8†). Nevertheless, the prominent CuI signal in the middle of the spectrum introduces uncertainty in CSA quantification and may obscure additional Cu signals, although the likelihood of their presence is low.

A *C*_Q_(^65^Cu) value of 22.0(3) MHz and *η*_Q_ of 0.02(2) were obtained from {[Cu(i)][Cu(ii)(pdc)(H_2_O)]·1.5MeCN·H_2_O}_*n*_; the near-zero *η*_Q_ value is in good agreement with the high local rotational symmetry at Cu(i) indicated from the single crystal XRD structure.^72^ The very slight departure from perfect *C*_3_ rotational symmetry indicated by the *η*_Q_ value of 0.02 can be traced to one of the Cu-bonded iodine atoms, which lies slightly out of a truly *C*_3_ symmetrical ligand arrangement. The dominance of the QI on ^63/65^Cu NMR spectral appearance, paired with the high signal-to-noise ratio, indicates that there is very little paramagnetic influence on the Cu(i) NMR parameters. The lack of paramagnetic effects can be attributed to two reasons. First, the paddlewheel Cu_2_ dimer in its ground state is an antiferromagnetically coupled spin singlet due to the short Cu–Cu bond length;^73^ the EPR spectrum of this MOF yielded a *g*-value of 2.161 (Fig. S7(b)†), which falls in the range of reported *g*-values for MOFs containing a Cu_2_ dimer in paddle-wheel units.^74–78^ Second, the distance between the Cu(i) and Cu(ii) dimers is 7.05 Å, which is long enough that the Cu(i) spin energy levels are only perturbed to a minor degree by any paramagnetic interaction.

**Fig. 4:**
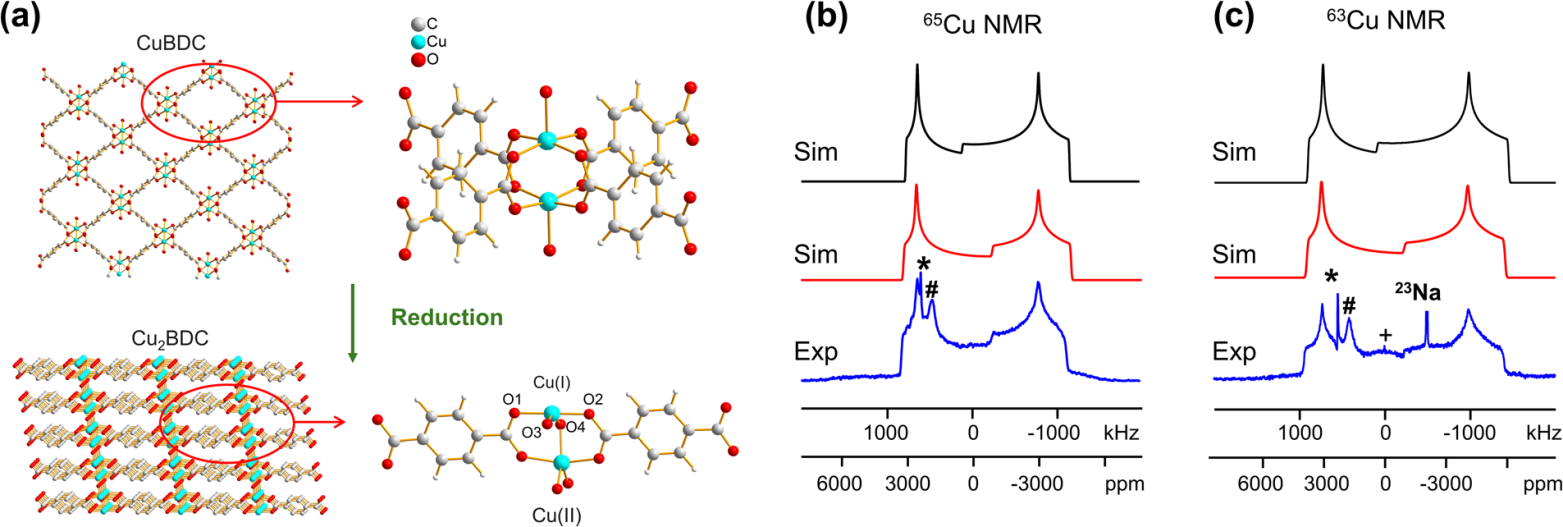
(a) The reduction of CuBDC to Cu_2_BDC and the local environment of Cu(i) in CuBDC and Cu_2_BDC is pictured. (b) Blue experimental (b) ^65^Cu and (c) ^63^Cu static NMR spectra of Cu_2_BDC at 21.1 T are shown, along with simulations incorporating CSA effects (red trace) and neglecting CSA effects (black trace). Note the effects of CSA on the central spectral discontinuity. The signal from ^23^Na background is also indicated in the ^63^Cu NMR spectrum but truncated for clarity.

In addition to the direct synthesis of Cu (i/ii) MOFs, a post-synthetic approach to Cu(i/ii) MOFs affords alternate avenues for tuning MOF properties; however, it is difficult or impossible to obtain diffraction-caliber single crystals of product using this approach. Potential applications for ^63/65^Cu solid-state NMR in the characterization of post-reduction Cu(i)-containing MOFs were explored by examining the case of Cu_2_BDC synthesis from the reduction of CuBDC. Cu(ii) sites in the two-dimensional CuBDC (BDC, 1,4-benzendicarboxylic acid) MOF can be partially reduced with l-ascorbic acid (LA acid) *via* post-synthetic modification to introduce Cu(i) sites, forming a three-dimensional Cu_2_BDC MOF (Fig. 4(a)).^14^ Powder XRD (Fig. S9†) clearly indicates that Cu_2_BDC resides in a different phase than the parent CuBDC MOF, but further analysis of the Cu local environment is hampered by the difficulties in obtaining Cu_2_BDC single crystals. The parent CuBDC MOF contains a paddlewheel local structure about Cu, where each Cu(ii) center is linked to four carboxylic groups from BDC linkers along with one water molecule, forming a stacked layered structure held together through intermolecular interactions. After LA-acid post-synthetic modification to produce the Cu_2_BDC MOF, half of the Cu(ii) sites in the MOF were reduced to Cu(i) (Fig. 4(a)). The four-coordinate Cu(i) center in Cu_2_BDC resides in a local CuO_4_ environment of seesaw geometry, with Cu(i) connected to two carboxylic oxygen atoms (O1, O2) from two BDC ligands, one oxygen atom (O3) of a water molecule, and one oxygen atom (O4) of a bridging OH group. The successful reduction of Cu(ii) centers to Cu(i) was confirmed by Cu 2p_3/2_ X-ray photoelectron spectroscopy (XPS, Fig. S9†) and X-band EPR (Fig. S10†). The ^63/65^Cu NMR spectra of Cu_2_BDC (Fig. 4(b and c)) features a QI-dominated NMR powder pattern that yields *C*_Q_(^65^Cu) = 53.0(3) MHz and *η*_Q_ = 0.22(3); the well-defined spectrum with relatively sharp features is also indicative of a highly ordered local structure. Although Cu(i) is four-coordinate in this system, the *C*_Q_ value is much larger than those of other four-coordinate Cu(i) centers previously discussed. This discrepancy arises from the seesaw local geometry about Cu(i) in Cu_2_BDC, which is a much more significant deviation from tetrahedral symmetry *versus* previous examples of distorted tetrahedral geometry.

A CSA span value of 1800 ppm was necessary to achieve good agreement between the simulated and experimental ^63/65^Cu NMR spectra of Cu_2_BDC (Fig. 4(b and c)), owing to the hyperfine interaction. The presence of paramagnetic centers, with their associated unpaired electrons, influences the NMR spectral appearance and CSA parameters of nearby diamagnetic nuclei.^79–83^ The corresponding hyperfine interactions between Cu(ii) unpaired electrons and Cu(i) nuclei leads to very large ^63/65^Cu NMR span values. We have performed localized molecular orbital calculations on Cu_2_BDC, which revealed that the unpaired electrons of Cu(ii) are indeed able to sample regions proximate to Cu(i) (Fig. S11†); this finding, together with the unremarkable Cu chemical shift of 200 ppm, indicates that an electron delocalization effect is present rather than a spin-polarization effect.^79,82,83^ This shows how ^63/65^Cu NMR can be a robust local characterization technique in the presence of proximate paramagnetic Cu(ii) centers, extending the applications of this technique to a wider variety of Cu MOFs.

### MOFs with three-coordinate Cu(i) sites

#### Cu(bpy)_1.5_NO_3_·1.25H_2_O

**Fig. 5:**
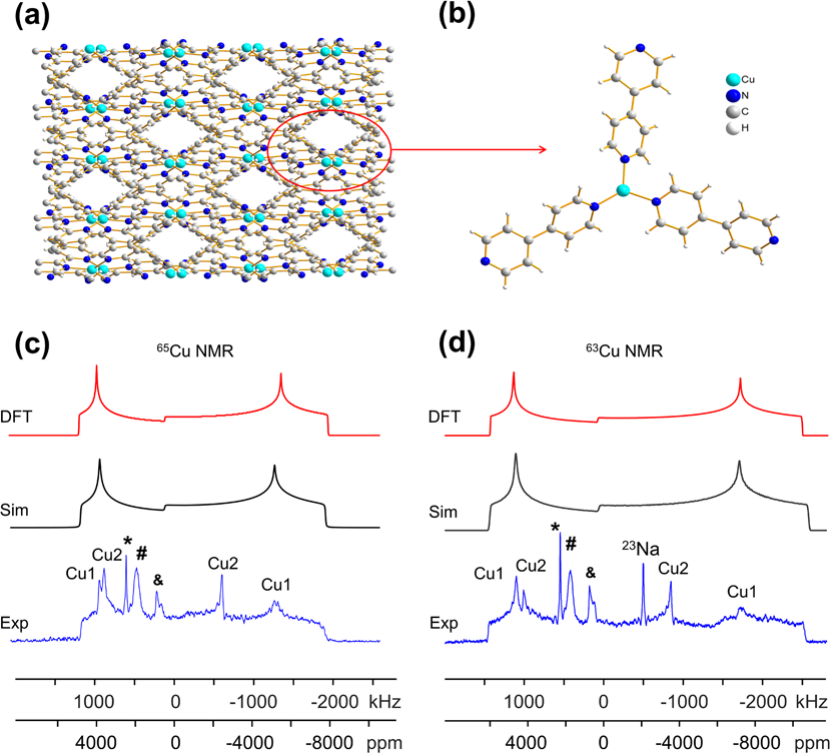
(a) The 3D framework structure and (b) local structure of the Cu(bpy)_1.5_NO_3_·1.25H_2_O MOF. The charge-balancing NO_3_^−^ anion and guest water molecules are omitted for clarity. (c) ^65^Cu and (d) ^63^Cu static NMR spectra of Cu(bpy)_1.5_NO_3_·1.25H_2_O at 21.1 T; the blue traces are experimental, black are simulated, and red are simulated using NMR parameters from DFT calculations.

One of the first reported MOFs containing three-coordinate Cu(i) was Cu(bpy)_1.5_NO_3_·1.25H_2_O in the 1990s.^15^ This MOF has applications in anion exchange due to weak bonding between the nitrate ions and the framework. In addition, Cu(bpy)_1.5_NO_3_·1.25H_2_O and its analogue {[M_2_(4,4′-bpy)_3_(NO_3_)_4_]·*x*H_2_O}_*n*_ (M = Co, Ni, Zn) MOFs have demonstrated reversible adsorption of small molecules such as CH_4_, N_2_, and O_2_.^84^ Cu(bpy)_1.5_NO_3_·1.25H_2_O crystallizes in the orthorhombic space group *Fddd* and is cationic, featuring large rectangular channels propagating along the [100], [010], and [001] axes which measure 26 × 20, 10 × 12, and 43 × 18 Å, respectively (Fig. 5(a)). The pores are occupied by anionic charge-balancing nitrate anions along with water molecules. The unit cell of the as-made MOF contains one unique Cu(i) site bound to three nitrogen atoms originating from three separate 4,4′-bpy linkers in a distorted trigonal planar CuN_3_ geometric arrangement (Fig. 5(b)), along with water molecules.

The ^6^3^/6^5^^Cu static solid-state NMR spectra of the as-made hydrated Cu(bpy)_1.5_NO_3_·1.25H_2_O MOF at 21.1 T are shown in Fig. 5(c and d). The local Cu environment is significantly distorted from trigonal planar symmetry (∠N_1_–Cu–N_2_ = 125.39°, ∠N_1_–Cu–N_3_ = 125.71°, ∠N_2_–Cu–N_3_ = 108.54°), which leads to an increased *C*_Q_(^63/65^Cu) value and spreads the ^6^3^/6^5^^Cu NMR spectral powder patterns across breadths of *ca*. 3 MHz and 4 MHz, respectively. The ^63/65^Cu NMR spectra feature a broad signal with some additional details, along with metallic copper (*) and background signals (#).

A successful simulation of all spectral features is challenging due to the multiple Cu powder patterns, despite the single Cu(i) site present in this MOF. Several spectral simulation strategies were explored, but only one produced a satisfactory fit (Fig. S12†), which is discussed below. The extremely broad underlying powder pattern with corresponding quadrupolar horns marked “Cu1” in Fig. 5, which has the highest integrated ratio of *ca*. 80%, originates from the Cu(bpy)_1.5_NO_3_·1.25H_2_O MOF. The extracted parameters are *C*_Q_(^65^Cu) = 74.0(4) MHz, *C*_Q_(^63^Cu) = 79.0(6) MHz, *η*_Q_ = 0.18(2), and *δ*_iso_ = 300(100) ppm, where both *C*_Q_ values are remarkably high among reported values.^35,51^ This assignment is also supported by DFT calculations, which yielded *C*_Q,calc_ (^65^Cu) = 74.5 MHz and *η*_Q,calc_ = 0.17 (Table 1, Fig. 5(c and d)). Another narrower resonance labelled Cu2, with an integrated area ratio of *ca*. 18%, is assigned to a side product; the NMR parameters are reported in Table 1 for reference. The experimental PXRD pattern of Cu(bpy)_1.5_NO_3_·1.25H_2_O in Fig. S2† agrees well with the pattern simulated from the reported crystal structure, yet the experimental diffractogram also exhibits some additional reflections at low angles that are attributed to the Cu(i) impurity. There is also a trace amount of an unidentified impurity accounting for *ca*. 2% of total spectral intensity that is labelled with the “&” character and assigned to a Cu3 species.

#### ^63/65^Cu NMR of [Cu_3_(4hypymca)_3_]

**Fig. 6:**
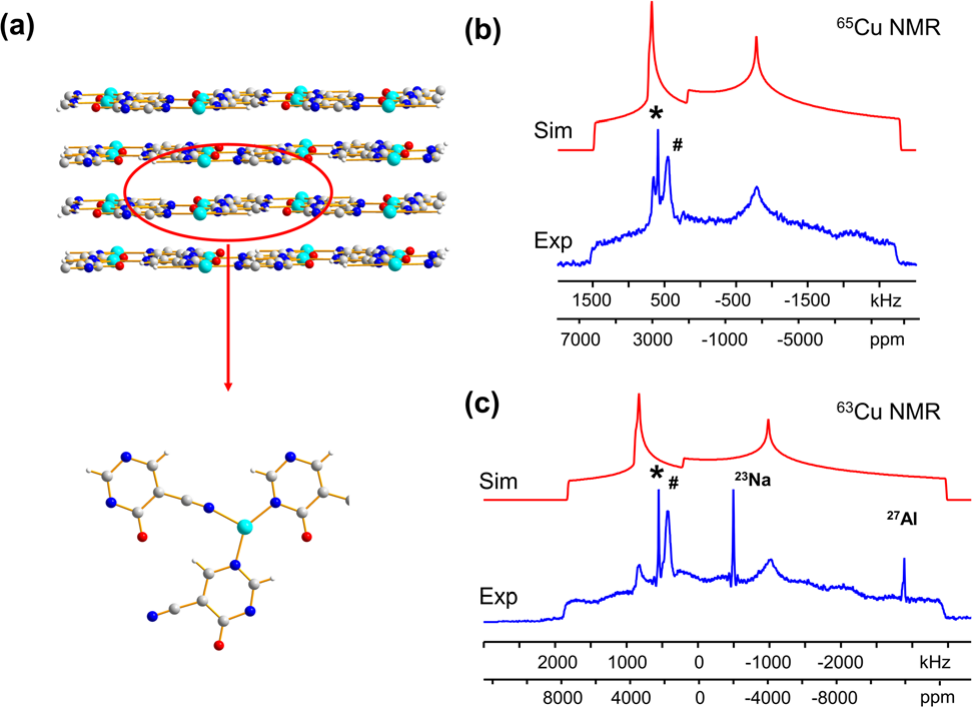
(a) The long-range and local structure of Cu_3_(4hypymca)_3_. The experimental (blue) and simulated (red) ^65^Cu static NMR spectra and ^63^Cu static NMR spectra at 21.1 T are shown in (b and c), respectively. ^27^Al marks a signal from probe background. The signal from ^23^Na background is also marked in the ^63^Cu NMR spectrum, but is truncated for clarity.

The electrically conductive two-dimensional [Cu_3_(4hypymca)_3_] MOF^13^ is composed of Cu(i) centers connected by 4-hydroxypyrimidine-5-carbonitrile (4hypymca) linkers. [Cu_3_(4hypymca)_3_] crystallizes in the orthorhombic system (*Pbcm* space group), with a long-range structure consisting of equidistant flat layered sheets (Fig. 6(a)). The single unique three-coordinate Cu(i) center is bound to N atoms from three separate 4hypymca linkers, forming a distorted trigonal planar local environment of relatively low symmetry about Cu. Accordingly, the ^63/65^Cu NMR spectra (Fig. 6(b and c)) are quite broad. The ^63^Cu NMR spectrum extends across a frequency range of *ca*. 5.3 MHz at 21.1 T, which corresponds to exceptionally large *C*_Q_(^63^Cu) = 80.6(4) MHz and *C*_Q_(^65^Cu) = 74.8(6) MHz values.

While both [Cu_3_(4hypymca)_3_] and Cu(bpy)_1.5_NO_3_·1.25H_2_O feature Cu(i) bound to three nitrogen atoms, the *η*_Q_ value is significantly higher in [Cu_3_(4hypymca)_3_] (Table 1). Cu(bpy)_1.5_NO_3_·1.25H_2_O features Cu coordinated to three N atoms of pyridine groups, but the Cu center in [Cu_3_(4hypymca)_3_] is connected to two pyridine-based N atoms and one nitrile N atom, which corresponds to decreased axial symmetry about Cu and a higher *η*_Q_ value. The average trigonal distortion (
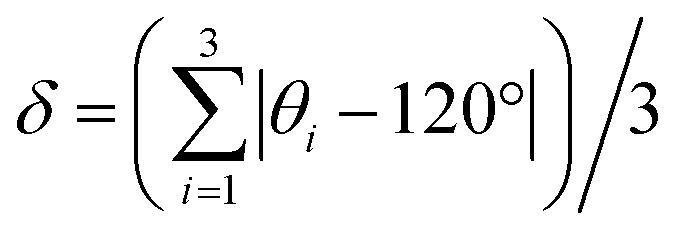
, where *θ*_*i*_, *i* = 1, 2, 3 are the three ∠N–Cu–N bond angles around Cu) is also larger in [Cu_3_(4hypymca)_3_] than in Cu(bpy)_1.5_NO_3_·1.25H_2_O by 8.0°, which further explains the increase in *η*_Q_.

### MOFs with two-coordinate Cu(i) sites

**Fig. 7:**
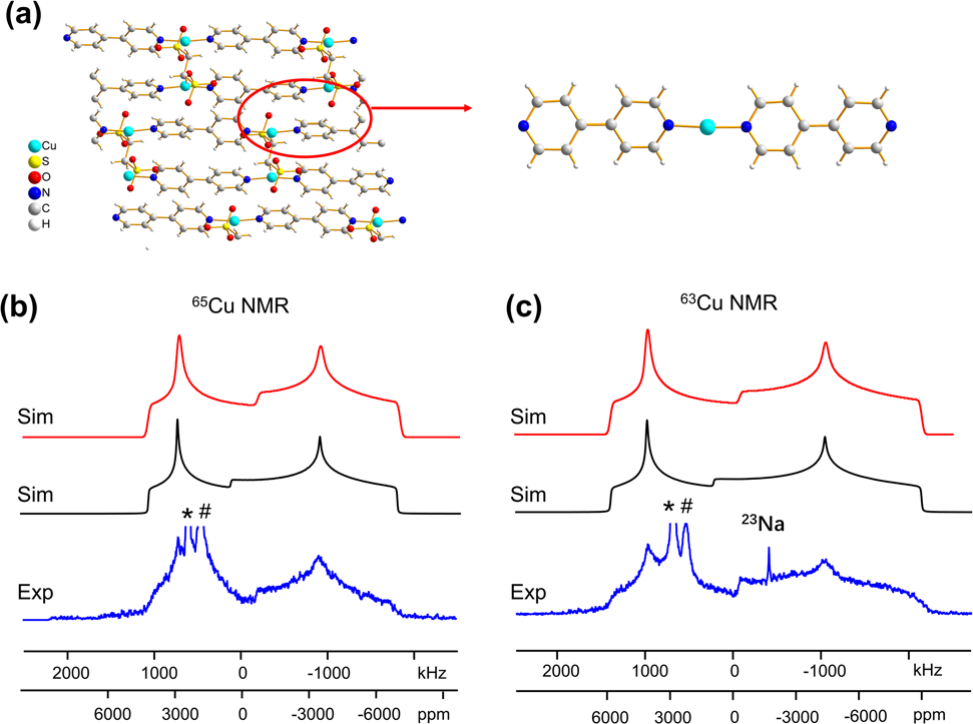
(a) The 3D framework structure and local structure of the SLUG-22 MOF. The (b) ^65^Cu and (c) ^63^Cu static NMR spectra at 21.1 T are shown in blue, along with the red simulation which includes CSA effects, and the black trace which does not include CSA effects. Note the difference in the central “divot” spectral feature between the red (CSA) and black (no CSA) simulations; CSA is necessary to properly fit this feature. The asterisks (*) denote the signal from metallic copper (Cu^0^) and the pound (#) marks the signal from probe background, which are truncated for clarity. The signal from ^23^Na background is also marked in the ^63^Cu NMR spectrum.

Some MOFs feature Cu(i) in two-coordinate linear arrangements. The SLUG-22 MOF is composed of Cu_2_(4,4′-bpy)_2_ units, where the two-coordinate Cu(i) center is connected to two nitrogen atoms from separate 4,4′-bpy linkers in a distorted N–Cu–N linear geometry, with a long-range structure consisting of one-dimensional chains of infinite length (Fig. 7(a)). ^63/65^Cu static solid-state NMR spectra of SLUG-22 at 21.1 T (Fig. 7(b and c)) are extremely broad, owing to the low local symmetry of the linear two-coordinate environment at Cu and the correspondingly large *C*_Q_ value. Simulations (Table 1) necessitated the use of CS parameters, but indicated only a single Cu(i) site with *C*_Q_(^65^Cu) = 63.0(1.0) MHz and *η*_Q_ = 0.34(2) was present; this contrasts with the reported crystal structure^18^ that indicated there are two inequivalent Cu(i) sites. A careful examination of the ^63/65^Cu NMR spectra reveals slightly broader features in the experimental spectra, which could be indicative of two nearly identical overlapping Cu powder patterns that cannot be resolved. Indeed, the crystal structure shows that the Cu centers reside in very similar local environments.^18^ Furthermore, Fig. 7 illustrates how the frequency of the central spectral discontinuity is affected by CSA, and in particular, the exceptionally large span value of 1500 ppm.

In order to investigate the origins of the abnormally large Cu CSA in SLUG-22, EPR and XPS experiments were performed (Fig. S13†), which indicated this material was largely free of Cu(ii) or other paramagnetic impurities. The lack of any plausible hyperfine interactions indicates the sizeable CSA in SLUG-22 likely arises from the linear two-coordinate local geometry at Cu. Large CSA spans have been recorded from other transition metal compounds in linear configurations (*e.g.*, linear HgX_2_).^85^ We found that cluster DFT calculations using the RHF method and 6-31++G**/6-311++G** basis sets were the most reliable avenue for calculating Cu span values (*vide infra*, Table S5†); these particular calculations also predicted a substantial Cu CSA span value in SLUG-22 arising from the local linear coordination geometry at Cu.

There are more complicated MOFs featuring mixed coordinate Cu(i) local environments that can be examined using ^63/65^Cu NMR. We investigated the [Cu_6_I_6_(DABCO)_2_] framework, which contains four distinct Cu sites and produced a complicated Cu NMR spectrum that lacked clear singularities. The results and discussion regarding these experiments can be found in the ESI.†

### DFT calculations of EFG tensors

To obtain further insight into experimental NMR results and the local Cu(i) environments, plane-wave DFT calculations using GIPAW methods were performed. The EFG tensor parameters are sensitive to the local environment, which provides a metric to predict and optimize crystal structures.^86–92^ In this section, we examine Cu(i) structural insights obtained from plane-wave DFT calculations of ^63/65^Cu EFG tensors in MOFs. It should be noted that the XRD crystal structures were obtained at low temperatures while our Cu NMR experiments were performed at room temperature, which could lead to discrepancies between calculated and experimental Cu NMR parameters due to issues such as temperature-dependent unit cell dimensions and dynamics. To verify that temperature changes did not introduce significant changes to Cu NMR parameters, low-temperature NMR experiments were performed on selected MOFs, which yielded Cu NMR spectra nearly identical to room temperature spectra (Fig. S15†). This finding signified that, in this case, the experimental temperature did not play a significant role in the accuracy of calculated NMR parameters. This result is not particularly surprising, as all the MOFs investigated in this work have rather rigid frameworks.

GIPAW DFT calculations on the Cu MOF systems were evaluated by first examining the correlations between the calculated and experimental EFG tensor parameters in discrete Cu(i) coordination complexes with relevant Cu(i) local environments. The experimental Cu NMR data was taken from previous reports by Tang^35^ and Yu,^56^ using crystal structures obtained from the Cambridge Crystallographic Data Centre (CCDC). As shown in Fig. S16 and Appendix A of the ESI,† the calculated principal components of the Cu EFG tensor in this dataset exhibit a decreased *Γ*_RMSE_ (the EFG distance metric expressing the deviation between experimental and computed EFG parameters;^93^ see ESI†) after geometry optimization, from 0.071 a.u. before to 0.053 a.u. after. After geometry optimization, the slope of the respective plot is closer to 1, the *y*-intercept is reduced, and *R*^2^ is higher, which all indicate a better agreement with experimental values.

**Fig. 8:**
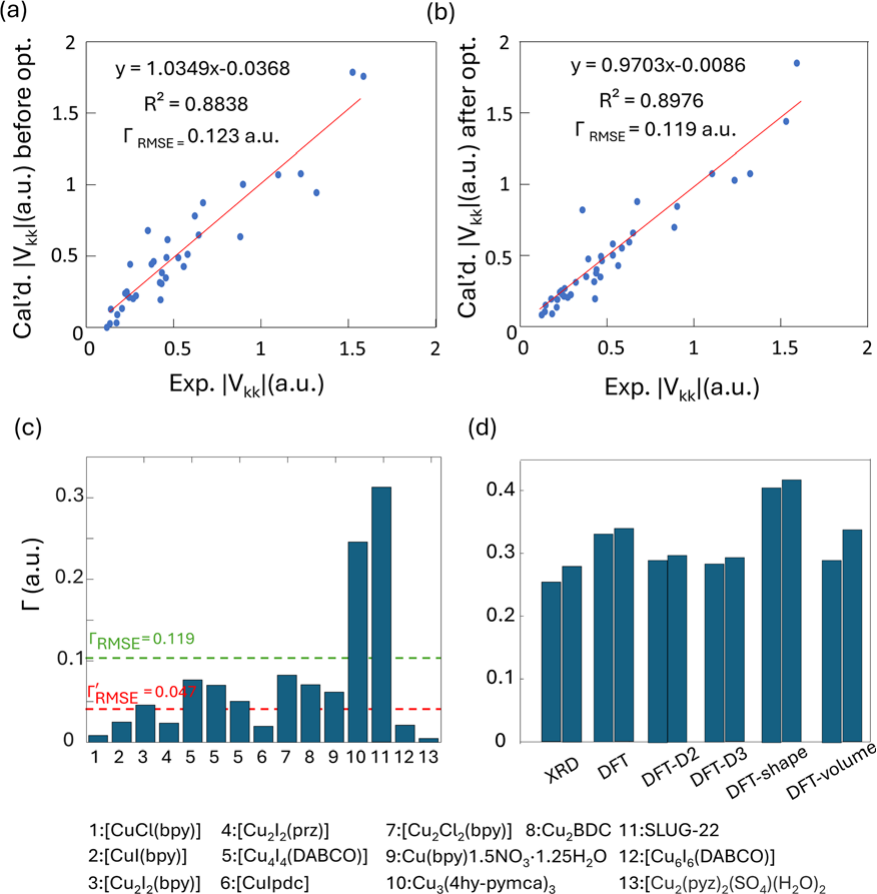
Relationships between the principal components (|*V*_*kk*_|, *k* = 1,2,3) of calculated and experimental ^63/65^Cu EFG tensors in MOFs (a) before and (b) after geometry optimization. (c) The *Γ* values for all MOFs after geometry optimization. The *Γ*_RMSE_ including the results from all MOFs is shown as a green dashed line, while the *Γ*_RMSE_ excluding SLUG-22 and Cu_3_(4hypymca)_3_ is shown as a red dashed line. (d) The EFG distance in the optimized SLUG-22 structure is shown, as obtained after plane-wave DFT calculations using different geometry optimization approaches. The *x*-axis labels in (d) are as follows; XRD structure: no optimization; DFT: geometry optimization without optimization of unit cell dimensions; DFT-shape: optimization with unit cell using a fixed-shape constraint; DFT-volume: optimization with unit cell dimensions using a fixed-volume constraint; DFT-D2 and DFT-D3: optimization using the D2 and D3 dispersion corrections with an optimized damping parameter (Fig. S17†).^86,93^ Note that there are two inequivalent but similar Cu sites in the reported XRD structure of SLUG-22.

The correlation between DFT-calculated and experimental Cu EFG tensor components |*V*_*kk*_| (*k* = 1, 2, 3) based on our current MOF dataset are shown in Fig. 8(a and b). After geometry optimization, the *Γ*_RMSE_ of the MOF Cu dataset was calculated to be 0.119 a.u., which is significantly larger than the 0.053 a.u. of the dataset constructed from prior reports. Bar plots of calculated *versus* experimental *C*_Q_ and *η*_Q_ values are shown in Fig. S25;†*C*_Q_ depends on a single EFG tensor component (*V*_33_) and is generally calculated quite accurately, while *η*_Q_ calculations are dependent on all three EFG tensor components and are therefore less accurate. The SLUG-22 and Cu_3_(4hypymca)_3_ MOFs are responsible for the increased *Γ* value in the MOF dataset (Fig. 8(c)). When excluding SLUG-22 and Cu_3_(4hypymca)_3_, the *Γ*_RMSE_ value is a much more reasonable 0.047 a.u. These results indicate that further investigation of the local structure and geometry optimization in SLUG-22 and Cu_3_(4hypymca)_3_ is warranted.

#### Discussion regarding the local structure of SLUG-22

There are three potential explanations for the significant differences observed between the experimental and calculated Cu EFG parameters of SLUG-22. The first possibility involves the presence of paramagnetic Cu(ii) that could impact spectral appearance and influence the accuracy of extracted Cu EFG parameters; however, XPS and EPR experiments (Fig. S13†) indicated no detectable amounts of Cu(ii) species. The second source is a potential temperature dependence of the SLUG-22 phase or unit cell dimensions, since the original XRD structure was obtained at low temperature and our NMR experiments were performed at room temperature. Hardware limitations prohibited low-temperature ^63/65^Cu NMR experiments at 21.1 T, and ^63/65^Cu WURST-CPMG NMR experiments at 9.4 T on these systems were not successful due to low *T*_2_ values. As a surrogate, the ^1^H–^13^C CP/MAS NMR spectra of SLUG-22 at 208 and 298 K were acquired and found to be quite similar (Fig. S18†), suggesting that no significant structural deviations or phase changes occur at lower temperatures. The third source is fundamental structural issues arising from the DFT geometry optimizations performed prior to EFG calculations, which was found to merit further investigation.

Four DFT optimization schemes of the SLUG-22 crystal structure were explored (Fig. 8(d)). All the geometry-optimized structures yielded lower SCF energies *versus* the XRD structure, yet the agreement between calculated and experimental Cu EFG tensor parameters using any of the calculation strategies did not show significant improvement, which is puzzling. A more detailed examination of the experimental PXRD patterns in the original work describing SLUG-22 ^18^ (Fig. S19(a)†) revealed that several intense reflections expected at low angles from the reported single crystal structure are not apparent in the original experimental data, particularly the prominent reflection at *ca.* 9°, while additional unexpected reflections are present. Our geometry-optimized structures generated using a myriad of DFT-based approaches failed to improve the agreement between experimental and calculated XRD patterns (Fig. S19(b)†). Based on the considerable deviation between the experimental and calculated PXRD patterns, along with the inaccuracy of calculated EFG tensor parameters, it appears that the reported single crystal structure of SLUG-22 is incorrect in some manner, which illustrates another practical application of Cu NMR.

#### Discussion regarding the local structure of Cu_3_(4hypymca)_3_

**Fig. 9:**
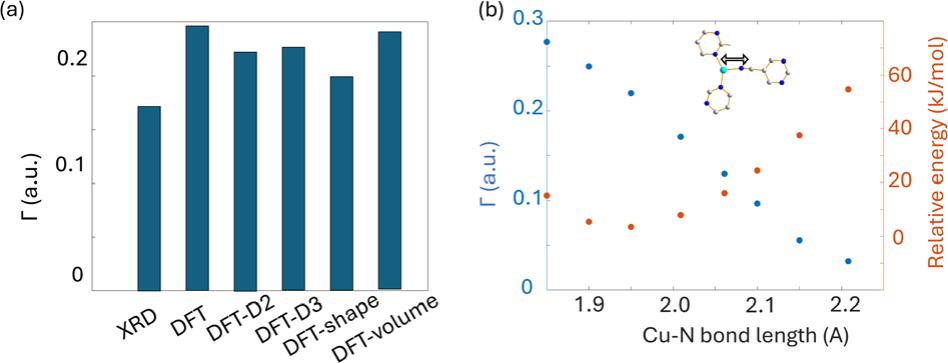
(a) The EFG distance of optimized Cu_3_(4hypymca)_3_ structure with different approaches, along with (b) the dependence of *Γ* on Cu–N bond length.

As with SLUG-22, we observed no improvement in the agreement between calculated and experimental EFG tensor components of the Cu_3_(4hypymca)_3_ MOF despite employing a variety of different geometry optimization strategies prior to NMR calculations (Fig. 9(a)). The most striking change in the local optimized geometry about Cu is a reduction of > 0.1 Å in the Cu–N bond length to the linker cyanide group. Variations in bond lengths and bond angles have a known influence on EFG parameters.^94,95^ To investigate further, we systematically altered the Cu–N bond length and then calculated the EFG tensor parameters and relative energy of the system, as shown in Fig. 9(b). While the minimum energy is associated with a Cu–N bond distance of 1.95 Å, this distance leads to a considerable gap between calculated and experimental Cu EFG tensor parameters. In comparison, a Cu–N bond length of 2.20 Å maximizes the accuracy of the calculated EFG tensor parameters, but results in an unacceptably high system energy. In this case, the most likely situation is a Cu–N bond length that results in a mutual minimization of system energy and *Γ*, where the trends intersect in Fig. 9(b). This data suggests that the Cu–N bond length in the Cu_3_(4hypymca)_3_ MOF crystal structure is slightly longer than the reported value of 2.00 Å. In addition, it is possible that plane-wave DFT calculations do not properly account for intermolecular interactions between the 2D sheets in this MOF, or there could be “slipping” of relative positions between the 2D MOF sheets that cannot be clearly identified *via* XRD studies.

#### Calculations of Cu EFG tensor orientations and CS tensor parameters

DFT calculations are known to reliably yield the orientation of the Cu EFG tensor, and the EFG tensor orientations for these Cu MOF systems along with a brief discussion are provided in Appendix B of ESI.† The calculated CS parameters are listed in Table 1 for reference, but we highlight that agreement between calculated and experimental CS values is rather poor (Fig. S26 in Appendix C†), and plane-wave DFT calculations evidently are not a reliable predictor of Cu CS parameters in MOFs at this time. While calculating EFG parameters only involves the electronic ground state of a system, CS calculations involve both the ground and excited states, which increases the computational complexity. For instance, Tang *et al.* performed CS calculations using different DFT basis sets and methods on discrete cluster models of Cu(i) compounds,^35^ but only obtained partial agreement with experimental results depending on the particular approach. Calculating CS parameters for heavier atoms such as Cu is also challenging due to factors such as spin–orbit effects,^96^ relativistic considerations,^97,98^ and the many possible hybrid functionals that can be applicable.^99^ The relatively high uncertainty of experimental CS values (Table S6†) as a result of multi-variable fitting may also influence the agreement with calculated CS values; larger uncertainties in the experimental CS parameters will generally result in poorer agreement with accurately calculated values.

We also performed calculations on geometry-optimized cluster models. The results using several different methods and basis sets are listed in Table S5.† The CS span values calculated using RHF/6-31++G** and RHF/6-311++G** (Fig. S21†) demonstrated better agreement with experimental span values when compared to plane-wave DFT calculations. In contrast, the calculated EFG tensors with all cluster models (Fig. S20†) yielded poorer agreement with experimental values when compared to plane-wave DFT calculations (Fig. 8(b)).

### Quadrupolar coupling constant and the Cu(i) coordination number

**Fig. 10:**
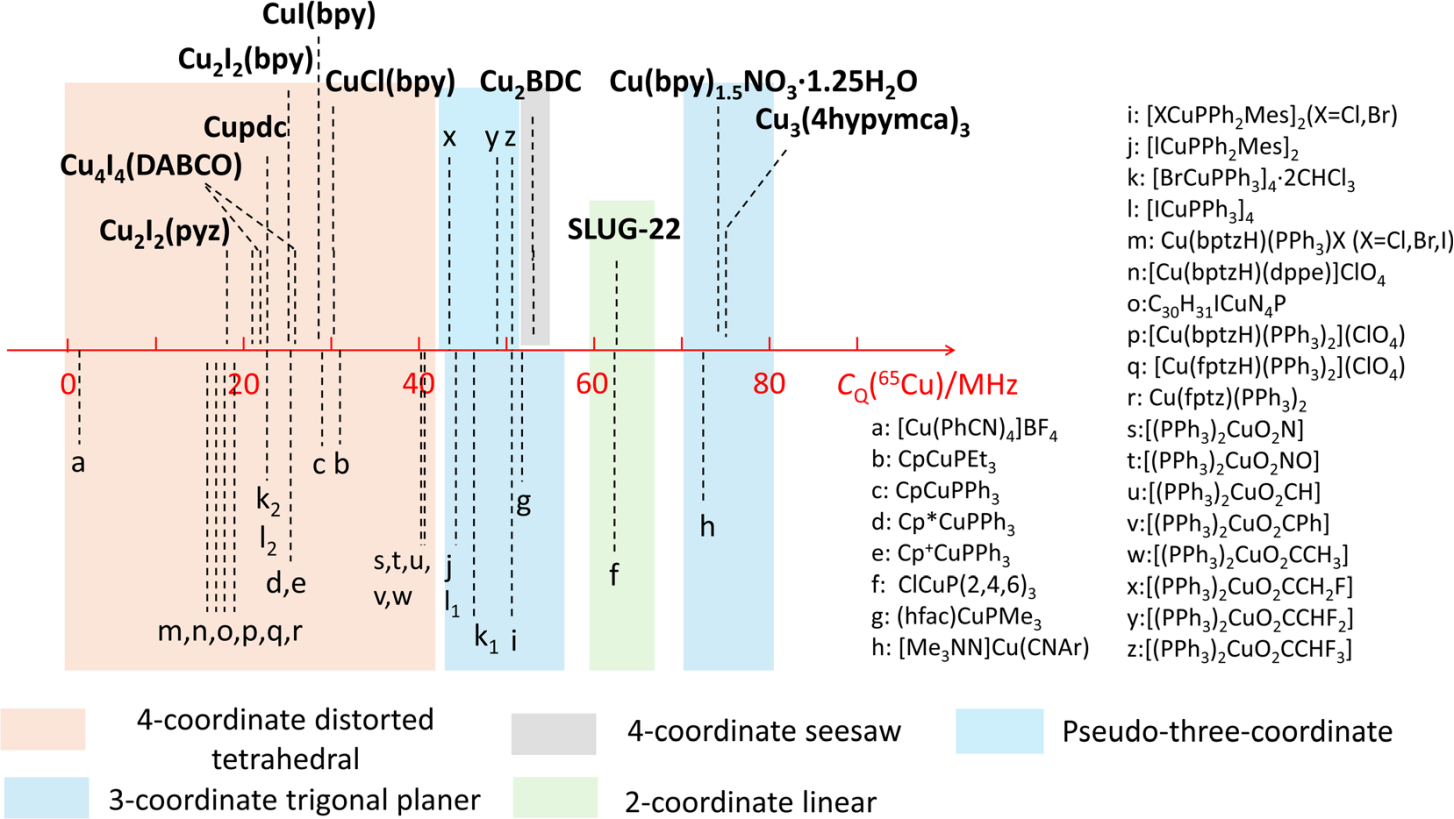
Copper *C*_Q_(^65^Cu) values of Cu(i) MOFs, along with those previously reported for other Cu(i) compounds.

The observed quadrupolar coupling constant (*C*_Q_) largely depends on the coordination number of Cu(i). A summary of the *C*_Q_(^65^Cu) values in MOFs is illustrated in Fig. 10, along with relevant values in small metal–organic coordination compounds from previous reports.^35,51,56^ The *C*_Q_(^65^Cu) values of four-coordinate tetrahedral Cu(i) centers are generally <40 MHz. The *C*_Q_(^65^Cu) values of three-coordinate Cu(i) range from 40 MHz to 80 MHz. Four-coordinate Cu(i) in a pseudo-three coordinate environment is correlated to *C*_Q_(^65^Cu) values between 40 and 50 MHz,^51^ which lies just between the bulk of four- and three-coordinate Cu environments. ^63/65^Cu NMR reports on two-coordinate Cu(i) ions are not common; both the SLUG-22 MOF in this work and the previously reported small molecule ClCuP(2,4,6)_3_^35^ yielded *C*_Q_(^65^Cu) values between 60 and 65 MHz. There were no prior ^63/65^Cu solid-state NMR reports of Cu(i) centers in a four-coordinate seesaw local geometry before this work; this environment appears to produce *C*_Q_ values comparable to three- and two-coordinate Cu(i) arrangements. The compiled empirical results from this and prior studies in Fig. 10 provides a convenient and general NMR-based tool to estimate the coordinate state of Cu(i) in unknown environments across a variety of materials and compounds.

### ^65^Cu solid-state NMR at 9.4 T

The Cu MOFs in this study were examined by ^63/65^Cu NMR using a high magnetic field of 21.1 T. Unfortunately, high fields are not always readily accessible, but there are several previous studies regarding ^65^Cu ultra-wideline NMR at 9.4 T.^35,51^ In MOFs, the Cu concentration is diluted, which poses an additional obstacle.

**Fig. 11:**
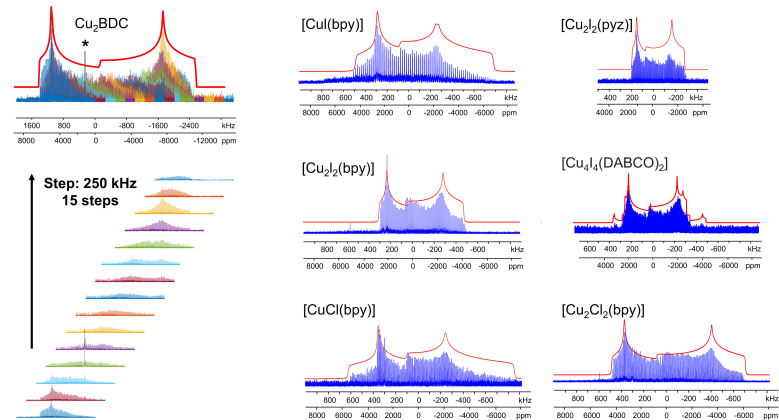
The experimental (blue) and simulated (red) ^65^Cu static WURST-CPMG NMR spectra of [CuCl(bpy)], [Cu_2_Cl_2_(bpy)] [CuI(bpy)], [Cu_2_I_2_(bpy)], [Cu_2_I_2_(pyz)] and [Cu_4_I_4_(DABCO)_2_] at 9.4 T are shown at right, along the 15 sub-spectra required to assemble the overall spectrum of Cu_2_BDC at left.

We set out to find if ^65^Cu solid-state NMR of Cu(i) MOFs was feasible at a more accessible field of 9.4 T (*i.e.*, *ν*_0_(^1^H) = 400 MHz), which would open up this technique to researchers across a broad swath of institutions. In addition, performing the Cu NMR experiments at different magnetic fields allows one to extract unambiguous CS and QI parameters along with the second-order quadrupolar isotropic shift. A major challenge at 9.4 T is spectral width; broadening from the second-order quadrupolar interaction is inversely proportional to *B*_0_, thus ^63/65^Cu NMR spectra are spread across a significantly larger frequency range at lower magnetic fields. To increase the signal-to-noise ratio and reduce experimental times, the WURST-CPMG pulse sequence can be employed.^100^ The WURST-CPMG sequence yields NMR spectra composed of a series of spikelets that trace out the overall spectral manifold, rather than the smooth continuous lineshape obtained from solid echo experiments. A spikelet spectrum can be acquired significantly faster than a solid echo spectrum. Using seven Cu(i) MOFs from this study as examples, we obtained ^65^Cu NMR spectra at 9.4 T ranging from *ca.* 500 to 3000 kHz in breadth (Fig. 11). The 9.4 T data was then simulated independently in order to assess the reliability of these results against those obtained at 21.1 T. The ^65^Cu NMR parameters obtained at 9.4 T (Table S6†) were consistent with those obtained from ^63/65^Cu experiments at 21.1 T, validating the accuracy of the extracted NMR parameters in Table 1. These findings prove that ^63/65^Cu NMR of Cu(i) MOFs and other Cu-dilute systems is experimentally viable at 9.4 T. The experimental times are listed in Table S4.†

### Applications of ^63/65^Cu solid-state NMR for anion exchange reactions

Anions are present in many MOFs to maintain charge balance with the cationic framework, which opens the door for versatile anion-exchange applications in fields such as the capture of undesirable pollutants (*e.g.*, ClO_4_^−^, HCrO_4_^−^).^101,102^ Understanding the chemistry taking place during anion exchange in MOFs is critical for the design of tailored MOFs to address specific applications. We used the Cu_2_(SO_4_)(pyz)_2_(H_2_O)_2_ MOF to demonstrate how ^63/65^Cu solid-state NMR at 9.4 T can be used to investigate Cu local structural evolution during chemical reactions such as anion exchange.

**Fig. 12:**
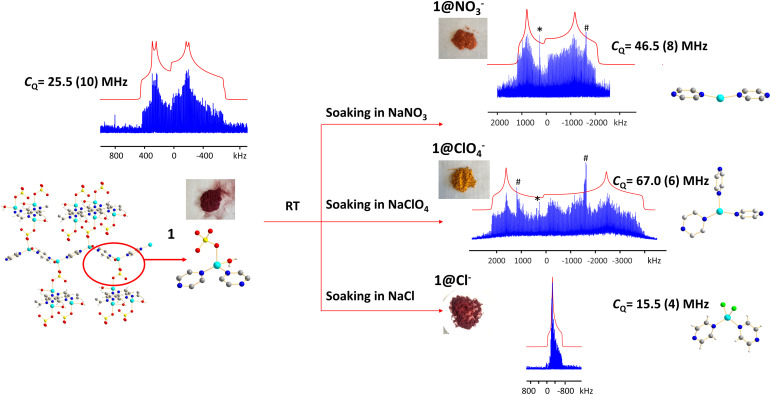
The ^65^Cu solid-state NMR spectra of 1 (Cu_2_(SO_4_)(pyz)_2_(H_2_O)_2_) and associated products after exposure to different aqueous solutions, as measured at 9.4 T. The asterisk (*) denotes a signal from metallic copper (Cu^0^) and the pound (#) indicates a resonance from a by-product containing Cu_2_O.

The (Cu_2_(SO_4_)(pyz)_2_(H_2_O)_2_) MOF, termed 1, was synthesized using CuSO_4_·5H_2_O and pyrazine in hydrothermal conditions.^103,104^ The Cu(i) center in 1 is in a distorted tetrahedral CuN_2_O_2_ local environment. Cu is connected to two pyrazine linkers, which form zig-zag one-dimensional chains that are bridged by sulfate ions, and Cu is also coordinated to water molecules that are oriented perpendicular to the 1D chains. The ^65^Cu solid-state NMR spectrum at 9.4 T (Fig. 12) features a well-defined powder pattern exhibiting a *C*_Q_(^65^Cu) of 25.2(2) MHz and a *η*_Q_ of 0.54(2), with the *C*_Q_ value lying in the established range of four-coordinate Cu (Fig. 10).

While 1 is stable in air and water, this material quickly turns from dark red to orange when exposed to aqueous NaNO_3_ solution, yielding 1@NO_3_^−^. A white insoluble precipitate is evident when BaCl_2_/HCl is added, indicating that a migration of SO_4_^2−^ from 1 into solution has occurred. As a soft acid, the Cu(i) ion prefers to coordinate with the soft base of nitrogen rather than the hard base of oxygen, which explains the formation of a precipitate. The ^65^Cu NMR spectrum of 1@NO_3_^−^ is a much broader *ca*. 3 MHz, which corresponds to a *C*_Q_(^65^Cu) of 46.5(8) MHz and a *η*_Q_ of 0.28(5) (Fig. 12). Using Fig. 10 as a guide, the significant increase in *C*_Q_(^65^Cu) from 1 to 1@NO_3_^−^ indicates that the local coordination at Cu has changed from four-coordinate to two- or three-coordinate. When 1 was immersed in a NaClO_4_ solution, the powder changed to an orange-yellow color, and the resulting product was termed 1@ClO_4_^−^. The ^65^Cu NMR spectrum of 1@ClO_4_^−^ at 9.4 T has an impressive breadth of *ca*. 7 MHz, with a *C*_Q_(^65^Cu) of 67.0(6) MHz and *η*_Q_ of 0.23(7); the increase in *C*_Q_ again indicates that Cu now resides in a two- or three-coordinate local environment. When 1 is exposed to aqueous NaCl, the original dark red color is retained, however, the ^65^Cu NMR spectral breadth is considerably narrowed and the lineshape is altered in a distinct fashion. The 1@Cl^−^ compound corresponds to a decreased *C*_Q_(^65^Cu) of 15.5(4) MHz, which indicates that the four-coordinate tetrahedral geometry is preserved at Cu, along with a significantly increased *η*_Q_ of 0.98(2). The well-defined ^65^Cu NMR powder patterns of 1 after exposure to NO_3_^−^, ClO_4_^−^ and Cl^−^ indicates that the local structure about Cu is relatively ordered in all instances. Using the estimated Cu coordination states obtained from the various ^65^Cu NMR spectra of 1 and its derivatives in mind, a search of the CCDC database was performed for any structures potentially matching or similar to the anion-exchanged products obtained in this study. Three compounds were identified: {Cu(pyz)(NO_3_)}_*n*_ which contains a two-coordinate CuN_2_ moiety,^105^ {Cu(pyz)_1.5_(ClO_4_)}_*n*_ with a three-coordinate CuN_3_ local structure,^105^ and {CuCl(pyz)}_*n*_ with a four-coordinate CuCl_2_N_2_ environment.^106^ The reported structures had been synthesized independently through solvothermal routes, and not *via* the anion-exchange approach we employed.

The experimental PXRD patterns of 1@NO_3_^−^, 1@ClO_4_^−^, 1@Cl^−^, along with the calculated PXRD patterns of {Cu(pyz)(NO_3_)}_*n*_, {Cu(pyz)_1.5_(ClO_4_)}_*n*_, and {CuCl(pyz)}_*n*_ from the reported solvothermal approaches are shown in Fig. S22.† The experimental PXRD pattern of 1@Cl^−^ matches perfectly with the calculated pattern of {CuCl(pyz)}_*n*_, indicating the anion-exchanged product 1@Cl^−^ is identical to solvothermally synthesized {CuCl(pyz)}_*n*._ This also confirms that a four-coordinate Cu(i) tetrahedral environment exists in 1@Cl^−^, as predicted from *C*_Q_(^65^Cu) NMR values. In contrast, the PXRD patterns of anion-exchanged 1@NO_3_^−^ and 1@ClO_4_^−^ look similar to those of solvothermally synthesized {Cu(pyz)(NO_3_)}_*n*_ and {Cu(pyz)_1.5_(ClO_4_)}_*n*_, but are not identical. It appears that the pairs of 1@NO_3_^−^ and {Cu(pyz)(NO_3_)}_*n*_ MOFs, and the 1@ClO_4_^−^, and {Cu(pyz)_1.5_(ClO_4_)}_*n*_ MOFs, are of similar connectivities but reside in different crystal structures (*i.e.,* space groups). The Cu(i) coordination numbers are two and three in {Cu(pyz)(NO_3_)}_*n*_ and {Cu(pyz)_1.5_(ClO_4_)}_*n*_, respectively, which is consistent with expectations based on the experimental 1@NO_3_^−^ and 1@ClO_4_^−^*C*_Q_(^65^Cu) values. The Cu(i) center is generally considered to be a soft acid, and preferentially binds with soft base ligands such as N donors and halogen ions, rather than with hard bases such as O donors. In good agreement, we observed that the formation of 1@Cl^−^ from 200 mg of 1 in a saturated aqueous solution concluded within *ca.* 30 min. In the context of hard and soft acids and bases, the cleavage of Cu(i)–O bonds to H_2_O and SO_4_^2−^ and the formation of Cu(i)–Cl bonds to yield a CuCl_2_N_2_ tetrahedral coordination environment in 1@Cl^−^ is favorable and should proceed quickly. In a similar finding, the reaction of 1 with NO_3_^−^ was also noted to conclude within 30 min; the zig-zag one-dimensional chains are sufficiently stable enough to exist without sulfate ions or coordinated water molecules, which then yields a two-coordinated Cu(i)N_2_ configuration with NO_3_^−^ solely as a charge balancing anion. In stark contrast, the formation of 1@ClO_4_^−^ requires *ca.* 12 hours of reaction time. After the cleavage of Cu–O bonds to H_2_O and SO_4_^2−^, the formation of Cu–N bonds to pyrazine linkers in a new trigonal geometry requires a much longer duration because perchlorate is a very weakly coordinating anion and is not directly bound to Cu(i).

## Conclusions

A series of Cu(i)-containing MOFs featuring Cu sites in different coordination environments have been examined using ^63/65^Cu ultra-wideline NMR and DFT calculations. The diversity of local environments of Cu(i) centers in MOFs leads to *C*_Q_(^65^Cu) values ranging from 18.8 to 74.8 MHz, which are diagnostic of the local Cu coordination environment and geometry. Multiple broad and overlapping ^63/65^Cu NMR signals arising from several unique Cu sites in MOFs can be resolved under favorable situations, and then simulated to extract information on the Cu local environment.

^63/65^Cu NMR spectroscopy provides direct evidence regarding the evolution of local Cu environments during MOF structural transformation processes. The sensitivity of this technique can also be exploited to monitor and characterize MOF phase transitions. Even in the challenging case of Cu(i/ii) mixed valence MOFs, ^63/65^Cu NMR spectra can be obtained, and are influenced by paramagnetic interactions when Cu(i) is especially proximate to Cu(ii). We have proven ^63/65^Cu NMR can be performed within reasonable experimental times at a lower magnetic field of 9.4 T despite the weight dilution of Cu(i) centers in MOFs. DFT-calculated ^63/65^Cu EFG tensor parameters have been presented and rigorously compared with experimental values; calculations using geometry-optimized structures generally lead to better agreement with experimental results except for two instances, and the origins of these disagreements were explored. We have established a list of *C*_Q_(Cu) values from this and previous studies that permits estimation of local Cu coordination using only the *C*_Q_ value, which is broadly applicable to many other Cu systems. This study highlights the versatility of ^63/65^Cu solid-state NMR, extending its relevance beyond MOFs and towards any chemical systems containing either abundant or dilute Cu(i) centers, with applications in fields such as catalysis, surface chemistry, solar cells, and biochemistry.

## Materials and Methods

### Sample preparation

The reported procedures were followed for MOF synthesis when possible.^12–16,18,65,107^ All details regarding synthesis and non-NMR characterization of the Cu compounds can be found in the ESI.†

### Solid-state NMR experiments

In general, ^63/65^Cu NMR spectra were acquired under static conditions using the solid echo or WURST-CPMG (Wideband Uniform Rate Smooth Truncation-Carr Purcell Meiboom Gill)^100,108^ pulse sequences. Solid echo experiments give rise to a smooth continuous lineshape, while WURST-CPMG experiments concentrate the signal into discrete spikelets that trace out the overall manifold of the powder pattern. Most of the ultra-wideline ^63/65^Cu NMR spectra in this work were too broad to be acquired in a single experiment, which necessitated the use of the VOCS (variable-offset cumulative spectra) method.^109^ The VOCS approach involves acquiring several sub-spectra at evenly spaced transmitter offsets using otherwise identical experimental parameters, and then co-adding the subspectra together to obtain the total ^63/65^Cu NMR spectrum. All ^63^Cu and ^65^Cu NMR spectra were referenced to solid CuCl at 0 ppm.

#### Solid-state NMR experiments at 21.1 T

Experiments at 21.1 T were conducted at the National Ultrahigh-field NMR Facility for Solids in Ottawa, Canada using a Bruker Avance II spectrometer. ^63^Cu and ^65^Cu NMR spectra were acquired using a home-built solenoid single-channel probe with a silver NMR coil (*ν*_0_(^63^Cu) = 238.73 MHz, *ν*_0_(^65^Cu) = 255.74 MHz). All ^63/65^Cu NMR spectra were acquired using a solid-echo pulse sequence (90°–90°). The interpulse delay was set to 30 μs. Additional experimental parameters are listed in Tables S2 and S3.† Background signals of the probe and various sample containers are discussed in footnote *b* of Table S3 and shown in Fig. S3.†

#### Solid-state NMR experiments at 9.4 T

All experimental parameters can be found in ESI and Table S4.†

### NMR simulations

Extraction of NMR parameters was performed using the WSolids software package.^110^ The experimental error bounds for each measured parameter were determined by visual comparison of simulated spectra; the parameter in question was varied bidirectionally from the best-fit value, keeping other parameters constant, until differences were observed. The reader is directed towards the “NMR interactions and NMR parameters” section in the ESI† for a discussion of NMR interactions.

### Quantum chemical calculations

The CASTEP Academic Release version code 19.11 ^111^ was used to calculate ^65^Cu magnetic shielding and electric field gradient (EFG) tensor parameters *via ab initio* plane-wave density functional theory (DFT) methods. Calculations were performed on the SHARCNET computational network (https://www.sharcnet.ca/). Perdew, Burke, and Ernzerhof (PBE) functionals were employed with the generalized gradient approximation (GGA)^112^ for the exchange correlation energy in all instances, with a plane-wave basis set cutoff energy of 800 eV. NMR parameters were calculated using “on-the-fly” ultrasoft pseudopotentials and the gauge-including projector-augmented wave (GIPAW) formalism.^113,114^ The DFT *C*_Q_ (MHz) values were obtained from calculated EFG tensor parameters using the most recently reported ^63/65^Cu quadrupole moment.^57^ The calculated ^63^Cu and ^65^Cu magnetic (chemical) shielding values (*σ*) in each MOF were converted to the corresponding chemical shift (*δ*) values using the formula *δ*_iso_ = *σ*_ref_ − *σ*_iso_. Note that *σ*_ref_ is the ^63/65^Cu shielding for the reference sample CuCl(s), where *σ*_ref_ (CuCl(s)) was calculated to be 702.68 ppm. Further details regarding geometry optimization schemes, along with the software and methodology used for cluster DFT calculations, can be found in the ESI.†

### EFG tensor analysis

The EFG tensor has three principal components denoted *V*_11_, *V*_22_, and *V*_33_, defined such that |*V*_33_| ≥ |*V*_22_| ≥ |*V*_11_|. The agreement between experimental *V*^exp^_*kk*_, *k* = 1, 2, 3 and *V*^cal^_*kk*_, *k* = 1, 2, 3 can be evaluated using the EFG distance metric *Γ*^86,93^ (in atomic units, a.u.). Please see the ESI† for a detailed explanation of EFG tensor parameters and *Γ*.

## Author contributions

W. Z.: conceptualization, investigation, formal analysis, methodology, computation, writing, review, editing; B. E. G. L.: writing, review, editing; V. V. T.: investigation, review, editing; S. C.: investigation; Y. H.: conceptualization, resources, funding, formal analysis, writing, review, editing.

## Conflicts of interest

The authors declare no conflicts of interest.

## Supplementary Material

SC-015-D4SC00782D-s001
