# Supplementary material for: Understanding Cu(i) local environments in MOFs via63/65Cu NMR spectroscopy
Source: Chem Sci. 2024 Feb 27;15(18):6690–706. doi: 10.1039/d4sc00782d (PMC11077522; doi:10.1039/d4sc00782d)
Supplement: SC-015-D4SC00782D-s001 [file SC-015-D4SC00782D-s001.pdf]

Electronic Supplementary Material (ESI) for Chemical Science.
This journal is © The Royal Society of Chemistry 2024

Supporting Information

Understanding Cu(I) Local Environments in MOFs via $^{63/65}$Cu NMR Spectroscopy

Wanli Zhang,[a] Bryan E.G. Lucier,[a] Victor V. Terskikh,[b] Shoushun Chen,[c] Yining Huang[a*]

[a] *Department of Chemistry, The University of Western Ontario, London, Ontario, Canada N6A 5B7.*

[b] *Metrology, National Research Council Canada, Ottawa, Ontario, Canada K1A 0R6.*

[c] *College of Chemistry and Chemical Engineering, Lanzhou University, Lanzhou, China 730000*

* Corresponding author; email: yhuang@uwo.ca.

Table of Contents

Supporting Items **Page**

1. Experimental Details. S4

2. Additional Results and Discussion. S9

The origin of EFG differences at Cu in Cu_2I_2(bpy) and Cu_2I_2(pyz) S9

Discussion of the assignment in MOF [Cu_4I_4(DABCO)$_2$]. S10

Discussion regarding [Cu_6I_6(DABCO)$_2$], a MOF with multiple Cu coordination environments S11

3. Additional Figures and Tables. S12

Table S1. MOF structural information S12

Table S2. ^{65}Cu static solid echo NMR experimental parameters at 21.1 T S13

Table S3. ^{63}Cu static solid echo NMR experimental parameters at 21.1 T S14

Table S4. ^{65}Cu static WURST-CPMG NMR experimental parameters at 9.4 T S15

Table S5. Experimental and calculated $^{63/65}$Cu NMR parameters in MOFs with cluster models S16

Table S6. Cu NMR parameters extracted from spectra obtained at 9.4 T and 21.1 T S24

Figure S1. Experimental (exp) and simulated (sim) PXRD patterns of [CuCl(bpy)], [Cu_2Cl_2(bpy)], [CuI(bpy)] and [Cu_2I_2(bpy)] S25

Figure S2. Experimental (exp) and simulated (sim) PXRD patterns of Cu(I) MOFs examined in this work. S26

Figure S3. ^{63}Cu NMR spectra of CuCl(bpy) that were obtained when the sample was packed in (a) glass and (b) Teflon tubes. (c) The ^{63}Cu NMR spectrum of probe background is shown, as obtained using an empty probe. S27

Figure S4. The TGA curve of [CuI(bpy)] measured under nitrogen atmosphere. S28

Figure S5. Experimental (blue) and simulated (red) $^{63/65}$Cu static solid echo NMR spectra of [CuCl(bpy)] and [Cu_2Cl_2(bpy)] at 21.1 T S29

Figure S6. The models used for DFT calculations on distinct molecules of [Cu_2I_2(bpy)] S30

Figure S7. (a) The Cu 2$p_{3/2}$ XPS spectrum of {[CuI][CuII(pdc)(H_2O)]·1.5MeCN· H_2O}$_n$, along with peak assignments in black; (b) the X-band EPR of {[Cu(I)][Cu(II)(pdc)(H_2O)]·1.5MeCN·H_2O}$_n$ MOF at 298 K S31

Figure S8. The ^{65}Cu (left) and ^{63}Cu (right) static solid echo NMR spectra of {[Cu(I)][Cu(II)(pdc)(H_2O)]·1.5MeCN·H_2O}$_n$ at 21.1 T S32

Figure S9. (a) The experimental (exp) and simulated (sim) PXRD patterns of CuBDC and Cu_2BDC, (b) The Cu 2$p_{3/2}$ XPS spectra of CuBDC and Cu_2BDC S33

Figure S10. The X-band EPR spectrum of Cu_2BDC at 298 K S34

Figure S11. An ilustration of the localized molecular orbital overlap between Cu(I) and Cu(II) metal centers in the Cu_2BDC MOF S34

Figure S12. Simulation of the experimental (blue) ^{65}Cu NMR spectrum of Cu(bpy)$_{1.5}$$NO_3$·1.25$H_2O$ S35

Figure S13. The X-band EPR spectrum at 298 K of SLUG-22 is shown in (a), along with the Cu 2$p_{3/2}$ XPS spectrum of SLUG-22 in (b) S35

Figure S14. (a) A schematic illustration of the long- and short-range structure within [Cu_6I_6(DABCO)$_2$] is displayed, along with (b) the experimental (blue) and simulated (red, with individual site contributions in orange and purple) ^{63}Cu static solid echo NMR spectra of [Cu_6I_6(DABCO)$_2$] at 21.1 T. (c) A simulated ^{63}Cu NMR spectra at 21.1 T, constructed using the DFT-calculated NMR parameters S36
Figure S15. The static ^{65}Cu NMR spectra of (a) [CuI(bpy)] and (b) Cu_2BDC at 9.4 T under 298 K and 208 K S37
Figure S16. The relationship between the calculated and experimental principal $^{63/65}$Cu EFG tensor components ($|V_{kk}|$,, k = 1,2,3) of small complexes (a) before and (b) after geometry optimization S37
Figure S17. The root mean square EFG distances (Γ_{RMSE}) between the experimental and calculated principal components of the Cu EFG tensors corresponding to the Cu(I) complexes plotted as a function of the damping parameter S38
Figure S18. The ^{1}H-^{13}C CP/MAS NMR spectra of SLUG-22, as acquired at 9.4 T and a temperatures of 298 K and 208 K S38
Figure S19. The simulated and experimental XRD patterns of SLUG-22 from previous accounts and from this work S39
Figure S20. A series of comparisons charts plotting the calculated EFG tensor components obtained using DFT cluster calculations versus the experimentally determined EFG tensor components S40
Figure S21. The experimentally obtained Cu MOF CS span values plotted against the calculated span values using a DFT cluster approach. The calculated span values were obtained using different methods and basis sets, as indicated at top of each graph S41
Figure S22. The experimental (blue) PXRD pattern of $Cu_2(SO_4)(pyz)_2(H_2O)_2$ (termed **1**) before and after anion exchange are shown, where the products are denoted as **1**@NO_3^-, **1**@ClO_4^-, and **1**@Cl^-, respectively S42
Appendix A: The calculated Cu EFG parameters of small molecules from prior reports S43
Appendix B: Discussion on the EFG tensor orientations S44
Appendix C: Correlation plots of EFG and CSA from plane-wave DFT and cluster calculations S46

4. References S48

1. Experimental Details

Sample synthesis.

All chemicals were purchased from Sigma-Aldrich and used as received without any further purification.

[CuCl(bpy)].[1] The addition of a 10 mL solution of 4,4'- bipyridine (0.78 g, 5.0 mmol) in acetonitrile to a 50 mL solution of CuCl (0.50 g, 5.0 mmol) in acetonitrile at room temperature resulted in the immediate formation of a dark red microcrystalline material which was collected via centrifugation, washed with ethanol followed by diethyl ether, and then dried under vacuum overnight.

[CuI(bpy)].[2] Solutions of CuI (20 mg, 0.11 mmol) in 5 mL acetonitrile and 4,4'-bipyridine (33 mg, 0.21 mmol) in 5 mL acetonitrile were combined and mixed. The resulting mixture produced relatively thick red plates of product after stirring overnight at room temperature. The resulting materials were collected by centrifugation, washed with ethanol followed by diethyl ether, and dried under vacuum overnight.

[Cu_2Cl_2(bpy)].[3] The [CuCl(bpy)] product was heated at 200 °C overnight under dynamic vacuum (≤ 1 mbar) while attached to a Schlenk line. The original deep red color of [CuCl(bpy)] changed to a pale-yellow color, and the resulting powder product was collected after cooling the apparatus down to room temperature.

[Cu_2I_2(bpy)].[3] A solution of 0.38 g (2.0 mmol) of CuI in 15 mL acetonitrile was added to a solution of 0.16 g (1.0 mmol) of 4,4'-bipyridine in 10 mL acetonitrile while stirring. The reaction mixture was then stirred at room temperature overnight, yielding a yellow powder that was collected and washed with EtOH and diethyl ether.

[Cu_4I_4(DABCO)].[4] 1,4-Diazabicyclo[2.2.2]octane (DABCO, 0.13 g, 1.2 mmol) was dissolved in 15 mL acetonitrile at 70 °C, and was then added to a solution of CuI (0.19 g, 1.0 mmol) in 15 mL acetonitrile at 70 °C. The resulting reaction mixture was stirred for 30 min. The product precipitated as a white powder, was collected by centrifugation, and washed with deionized water.

[Cu_6I_6(DABCO)].[5] A hydrothermal reaction of CuI (0.11 g, 0.56 mmol) and DABCO (0.02 g, 0.19 mmol) in a mixed solution consisting of 1 mL DMF and 6 mL acetonitrile (6 mL) solution at 150°C for 3 days. The mixture was then cooled to room temperature, yielding a pale yellow powder that was then washed three times with acetonitrile and dried under vacuum.

SLUG-22.[6] A mixture of copper acetate monohydrate (0.27 g, 1.35 mmol), 1,2-ethanedisulfonic acid (0.29 g, 1.52 mmol), 4,4′- bipyridine (0.21 g, 1.34 mmol) was established in 10 mL of water. This mixture was stirred at room temperature for 10 min and then transferred to a 15 mL Teflon-lined autoclave, which was heated in an oven at 175 °C for 4 days under autogenous pressure. The yellow needle-like crystalline product was isolated via vacuum filtration, washed with deionized water and acetone, and then dried under vacuum.

$Cu(bpy)_{1.5}NO_3\cdot 1.25H_2O$.[7] A mixture of $Cu(NO_3)_2\cdot 2.5H_2O$ (0.17 g, 0.74 mmol), 4,4'-bipyridine (bpy, 0.17 g, 1.11 mmol), and 1,3,5-triazine (0.04 g, 0.49 mmol) was created in 15 mL of deionized water, which was then transferred to a 25 mL Teflon-lined autoclave and placed into an oven. The oven temperature was raised from room temperature to 140 °C at a rate of 5 °/min and held at that temperature for 24 h. The oven was then cooled at a rate of 0.1 °C/min to 90 °C, after which the temperature was held constant for 12 h. Finally, the oven was cooled at a rate of 0.1 °C/min to 70 °C, held constant at 70 °C for 12 h, then cooled down to room temperature. The yellowish precipitate was vacuum filtered, washed with deionized water and ethanol and dried under vacuum.

$Cu_3(4hypymca)_3$.[8] Solution **1**: 0.9 mmol (100.0 mg) of 4-hidroxypyrimidine-5-carbonitrile was dissolved in 5 mL of dimethylformamide (DMF) and then 5 mL of distilled water was added. Solution **2**: 0.6 mmol (155.9 mg) of $CuCl_2\cdot 2H_2O$ was dissolved in 5 mL of distilled water and then 5 mL of DMF was added. Solutions **1** and **2** were then mixed in a closed 50 mL screw-cap jar, placed in an oven, and heated at 100 °C for 24 h. The greenish precipitate was centrifuged and washed with water.

Cu_2BDC.[9] 0.21 g of CuBDC was dispersed in 8 mL of deionized water with the aid of sonication for 10 min; 0.12 g of ascorbic acid was then added to the solution at room temperature. Following 1 h of sonication, the initially blue suspension changed color to white. The precipitate was collected, washed with DMF and deionized water three times, and dried under vacuum.

$\{[Cu^{I}I][Cu^{II}(pdc)(H_2O)]\cdot 1.5MeCN\cdot H_2O\}_n$.[10] A 15 mL solution of CuI (0.19 g, 1 mmol) in acetonitrile was mixed with 15 mL of pyridine-3,5-dicarboxylic acid (pdc, 0.17 g, 1 mmol) in DMF at room temperature, and the resulting solution was transferred into a 50 mL screw-top jar and mixed for 30 s in a ultrasonic bath, yielding a yellow mixture that was then placed in an oven at 100 °C for 24 h. The green precipitate was filtered, washed with DMF and acetonitrile, then dried under vacuum.

Powder X-ray diffraction. All powder X-ray diffraction patterns were recorded using a Rigaku SmartLab X-ray diffractometer operating with Cu Kα radiation (λ = 1.5406 Å). The reflections were collected at 2θ values ranging between 5 and 45 °, using an increment of 0.01 ° and an acquisition rate of 4 °/min. The PXRD patterns can be found in Figures S1 and S2.

Thermogravimetric analysis (TGA). All TGA measurements were performed on a TA Instruments Q50 thermogravimetric instrument under N_2 flow (40 mL/min), using a heating profile from 30 to 800 °C that was stepped at a rate of 10 °C/min.

Electron paramagnetic resonance (EPR). The EPR spectrum was collected on a Bruker ER200DSRC10/12 apparatus. The powder sample were packed in a 5 mm NMR tube for testing.

Solid-state NMR experiments at 9.4 T. ^{65}Cu SSNMR experiments were carried out on a Varian Infinity Plus spectrometer equipped with a 9.4 T Oxford Instruments superconducting magnet (ν_0(^{65}Cu) = 113.4 MHz) and a double channel (HX) 5 mm Varian/Chemagnetics static probe. The WURST-CPMG pulse sequence[11] was used with a WURST pulse length of 50 μs. A silver NMR coil was utilized to minimize the background signal from Cu metal. Several frequency-stepped subspectra were coadded to assemble the overall ^{65}Cu WURST-CPMG NMR spectra, following the variable offset cumulative spectrum (VOCS) protocol.[12] A total of 10-30 echoes in the CPMG train were collected per scan depending on the T_2(^{65}Cu) value in a particular system, and a ^{1}H decoupling field of 30 kHz was used in all instances.

NMR interactions and NMR parameters. A quadrupolar nucleus has an intrinsic electric quadrupole moment *(Q)*. There is an electric field gradient (EFG) at Cu(I) metal centers, which originates from the surrounding nuclear environment. *Q* and the EFG are coupled in a process known as the quadrupolar interaction (QI), which has a profound influence on Cu NMR spectra and thereby provides valuable information on local structure. The QI can be quantified using two parameters, the quadrupolar coupling constant (C_Q) and the asymmetry parameter (η_Q). C_Q is a measure of the QI magnitude; in general, more symmetric ground-state local electronic environments about Cu give rise to lower C_Q values and narrower Cu NMR spectra, while more asymmetric ground-state local electronic environments are linked to higher C_Q values and significantly broadened NMR spectra. The Cu(I) local environments found in MOFs often lead to relatively high C_Q values and broad $^{63/65}$Cu solid-state NMR resonances spread across

hundreds of kHz or even several MHz, which severely reduces the spectral intensity at any given point and renders signal acquisition challenging. The η_Q value is a measure of the axial symmetry of the EFG tensor and ranges from 0 to 1, with 0 corresponding to a perfectly axially symmetric tensor while 1 represents an axially asymmetry tensor. In a practical sense, η_Q is another measure of local symmetry about the Cu center and determines if the characteristic quadrupolar "horn" features in Cu NMR spectra are located nearer to the center ($\eta_Q \approx 1$) or nearer to the edges ($\eta_Q \approx 0$) of the spectral powder pattern. In addition to the QI, the $^{63/65}$Cu NMR signal in MOFs is influenced to a lesser degree by the chemical shift (CS) interaction, which offers additional information about local structure. The CS interaction itself, along with distinct spectral effects from its interplay with the QI, can introduce complicated spectral features such as the splitting of the quadrupolar "horns" that are challenging to simulate yet encode valuable structural information.

The quadrupolar coupling constant is defined as $C_Q = eQV_{33}/\mathrm{h}$, where the principal components of the EFG tensor are V_{11}, V_{22}, and V_{33}, ordered such that $|V_{33}| \geqslant |V_{22}| \geqslant |V_{11}|$. The EFG tensor can also be described *via* the asymmetry parameter η_Q, with $\eta_Q = (V_{11} - V_{22})/V_{33}$. The Euler angles ($\alpha$, β, γ) describe the relative orientation of the EFG and chemical shift (CS) tensors. The chemical shift (CS) interaction is modeled by a second-rank tensor with three mutually perpendicular components, δ_{11}, δ_{22}, and δ_{33}, ordered such that $\delta_{11} \geq \delta_{22} \geq \delta_{33}$. The Herzfeld-Berger convention describes the CS tensor using three unique parameters: the isotropic chemical shift (δ_{iso}), the span (Ω), and the skew (κ). The isotropic chemical shift (δ_{iso}) is calculated as the average of the CS tensor components, such that $\delta_{iso} = (\delta_{11} + \delta_{22} + \delta_{33})/3$. The CS span is defined as $\Omega = \delta_{11} - \delta_{33}$, while the skew is $\kappa = 3(\delta_{22} - \delta_{iso})/\Omega$.

Geometry optimization schemes with CASTEP. The crystal structures taken from the CCSD dataset were geometry optimized using CASTEP with a total energy convergence tolerance of 1×10^{-5} eV/ atom, maximum ionic force tolerance of 0.03 eV/Å, maximum ionic displacement tolerance of 0.001 Å, and a maximum stress component tolerance of 0.05 GPa.

The dispersion corrected geometry optimization used the Grimme (DFT-D2) two-body dispersion model, with the dispersion energy defined as:

$$E_{disp} = -\sum_{i,j>1}^{N} \frac{c_{6,ij}}{R_{ij}^{6}} f_{damp}(R_{ij}) \quad (1)$$

$$f_{damp}(R_{ij}) = \frac{1}{1 + \exp\left[-d\left(\frac{R_{ij}}{R_0} - 1\right)\right]} \quad (2)$$

where the R_0 is the sum of van der Waals' radii, and R_{ij} is the interatomic distance between the atomic pair of i and j. The $c_{6,ij}$ and d parameters are semi-empirical. The default parameters in CASTEP were used, except for the damping parameter (d), which was varied over the range $3.0 \leq d \leq 20.0$ with a step size of 1.0. Visualization of EFG tensors was performed using the *MagresView* code.[13]

The DFT-D3 geometry optimization technique is only available with CASTEP 20 and later versions. In this instance, CASTEP 20 was employed using the keyword SEDC SCHEME: D3-BJ.[14] In the current implementation, users cannot supply custom parameters, thus the default parameters were used.

EFG tensor analysis. The EFG tensor has three principal components denoted V_{11}, V_{22}, and V_{33}, defined such that $|V_{33}| \geq |V_{22}| \geq |V_{11}|$. The agreement between experimental V_{kk}^{exp}, k = 1, 2, 3 and V_{kk}^{cal}, k = 1, 2, 3 can be evaluated using the EFG distance metric Γ [15,16] (in atomic units, a.u.), defined such that:

$$\Gamma = \left(\frac{1}{15}[3\Delta_{11}^2 + 3\Delta_{22}^2 + 3\Delta_{33}^2 + 2\Delta_{11}\Delta_{22} + 2\Delta_{11}\Delta_{33} + 2\Delta_{22}\Delta_{33}]\right)^{\frac{1}{2}}, \quad (3)$$

$$\Delta_{kk} = \left|V_{kk}^{cal}\right| - \left|V_{kk}^{exp}\right|. \quad (4)$$

The EFG distance metric Γ expresses the deviation between an experimental EFG and a computed one using a single scalar value (in a.u.). The root-mean-square of M distances is calculated as

$$\Gamma_{RMSE} = \left(\frac{1}{M}\sum_{m} \Gamma_m^2\right)^{\frac{1}{2}}. \quad (5)$$

Quantum chemistry calculations with cluster models. Copper EFG and CSA tensor parameters were calculated using Gaussian 16[17] running on the SHARCNET computer network. The restricted Hartree–Fock (RHF) or DFT (B3LYP functional[18,19]) method was utilized with

the 6-31++G** and 6-311++G** basis sets.[20] The 2023 version of the Amsterdam Density Functional (ADF) program[21] was also used to calculate the NMR parameters. Scalar and spin–orbit relativistic effects were included using the zeroth-order regular approximation (ZORA). The revised Perdew, Burke, and Ernzerhof generalized gradient approximation exchange-correlation functional (GGA revPBE) and PBE0 functional[22,23] (25% HF exchange, hybrid form of PBE) were used. The basis sets used for all atoms were Slater-type triple-ζ with polarization functions (TZ2P). The localized molecular orbital analysis is with NBO 6.0 code,[24] and visualization was performed using the Amsterdam Modeling Suite running on the SHARCNET computer network. The EFG parameters were extracted using the *EFG*Shield software.[25]

2. Additional Results and Discussion

The origin of EFG differences at Cu in Cu_2I_2(bpy) and Cu_2I_2(pyz). To evaluate the impact of the (bpy) and (pyz) linker lengths and structures on Cu NMR parameters, DFT calculations were performed on three simplified models of Cu local structure (Figure S6). Structures 1 and 3 were directly extracted from the reported crystal structures of [Cu_2I_2(bpy)][3] and [Cu_2I_2(pyz)],[26] while structure 2 was obtained via manually replacing the 4,4'-bipyridine ligand with the pyrazine ligand coordinates from structure 3 ([Cu_2I_2(pyz)]).

There are two main differences between the local configurations of structure 1 and structure 3: (i) the specific locations of the atoms directly coordinated to Cu, and (ii) the ligand length. The purpose of structure 2 is to keep the coordinates of atoms directly bound to Cu fixed in order to investigate the effect of linker length. In both [Cu_2I_2(bpy)] and [Cu_2I_2(pyz)], DFT calculations orient the V_{33} component of the EFG tensor along or nearly along the Cu-N bond, which means that any changes involving the linker will have a significant impact on C_Q($^{63/65}$Cu). The Cu NMR calculations for structures 1 and 3 do not replicate experimental observations particularly well, but calculations do correctly predict the differences in C_Q and η_Q between [Cu_2I_2(bpy)] and [Cu_2I_2(pyz)]. Calculations involving structure 2 confirm that both the nature of linker and the local geometry of coordinated atoms affect the Cu NMR parameters in this case. More specifically in this system, changes in the linker structure at longer distances from Cu lead to decreases in C_Q and increases in η_Q, while linker modifications more proximate

to Cu slightly decrease both C_Q and η_Q. These model structure calculations illustrate how the NMR parameters of Cu(I) in MOFs are not only sensitive to the identity of bound atoms and their respective bond lengths/angles in the immediate coordination environment but are also reflective of longer-range interactions beyond directly bound atoms (*i.e.*, the effect of different linkers).

Discussion of the assignment in MOF [$Cu_4I_4(DABCO)_2$]. In order to assign the powder patterns to crystallographic sites, structural features must be correlated to the Cu local environment. DFT calculations orient the largest component of the EFG tensor, V_{33}, along the Cu-N bond. The local symmetry in the plane perpendicular to V_{33} around the Cu1 and Cu2 sites (*i.e.*, the distribution of ∠N-Cu-I bond angles and bond lengths[4]) determines the magnitudes of the V_{11} and V_{22} tensor components. The relative values of V_{11} and V_{22} determine the Cu η_Q value, and can be used to assign the $^{63/65}$Cu NMR signals to crystallographic Cu sites. The standard deviation of ∠N-Cu-I bond angles and Cu-I bond lengths is smallest about Cu3, which indicates this site should exhibit higher symmetry in the plane perpendicular to V_{33} and thus yield the lowest η_Q value of all three Cu sites. A comparison of local structural parameters between Cu2 and Cu1 shows that Cu2 has both the larger Cu-I bond length distribution and ∠N-Cu-I distribution of all Cu sites; this combination reflects a relatively lower symmetry in the Cu2 local environment perpendicular to V_{33} and should result in a relatively higher η_Q value. In contrast, the ∠N-Cu-I bond angle and Cu-I bond length distributions are smaller about Cu1, which translates to a more symmetrical Cu local environment perpendicular to V_{33} and should correspond to a lower η_Q. Cu2 is thus assigned to the signal with η_Q = 0.14(4) and Cu1 is assigned to the signal with a smaller η_Q of 0.09(3); the high-frequency portion of the spectral "horn" at ca. +200 kHz (*i.e.*, 700 ppm) in both the ^{63}Cu and ^{65}Cu NMR spectra belongs to the Cu1 signal, while the lower-frequency portion of the same horn arises from Cu2. This assignment is also consistent with DFT calculations (Table 1, η_Q(Cu2) > η_Q(Cu1) > η_Q(Cu3)). Note that the Cu-I bond length of *ca*. 3.4 Å in this MOF has been classified as a weak semicoordinative Cu···I contact in a different system,[27] which implies that there could be a long-range halogen bond type contact in this instance.

Discussion regarding [Cu_6I_6(DABCO)$_2$], a MOF with multiple Cu coordination environments. There are several MOFs that feature Cu(I) with different coordination numbers and environments.[5] In the [Cu_6I_6(DABCO)$_2$] framework, there are four inequivalent Cu(I) sites in a given Cu_6I_6 cluster (Figure S14(a)). The Cu1, Cu2, and Cu4 centers all reside in a four-coordinate distorted tetrahedral local environment, while Cu3 is in a nearly linear two-coordinate arrangement. The ^{63}Cu NMR spectral lineshape of [Cu_6I_6(DABCO)$_2$] (Figure S15(b)) is rather featureless and lacks obvious singularities, owing to the presence of multiple overlapping signals and/or disordered local structures. Despite the presence of four unique Cu centers, simulations reveal that only two major signals are present, termed S1 and S2 (Table 1). The Cu4 site is in a CuI_4 local environment and should have the smallest C_Q value, while both Cu1 and Cu2 reside in $CuNI_3$ motifs and should exhibit relatively larger C_Q values. The Cu3 site is two-coordinate and bound to two I atoms, which should give rise to an extremely high C_Q value due to the lack of local symmetry about Cu.

There are two possible factors accounting for the unexpectedly small observed C_Q value in this system. The distances between the two-coordinate Cu3 and Cu1/Cu2 centers are approximately 2.7-2.8 Å, which is shorter than the 3.92 Å sum of two copper van der Waals radii; two copper centers at this distance may be participating in a cuprophilic interaction,[28,29] which could reduce experimental C_Q(Cu) values. There are also two iodine atoms that are not formally bound to Cu3, yet still reside in the extended Cu3 coordination sphere; the Cu3-I distance is 3.348 Å can be classified as a weak semi-coordinative Cu-I contact.[27] This puts Cu3 in a pseudo-four-coordinate environment, which increases local symmetry at Cu and gives rise to an unexpectedly low C_Q value that is in the regime of distorted four-coordinate Cu centers. The calculated C_Q(Cu) values are shown in Figure S15(c).

3. Additional Figures and Tables

Table S1. MOF structural information.

**MOF**	**Dimensionality**	**Cu(I) coordination**	**Number of Cu(I) sites**	**Local geometry at Cu(I)**	**Organic linker**
[CuCl(bpy)]	3D	$CuCl_2N_2$	1	Tetrahedral	4,4'-bipyridine
[CuI(bpy)]	3D	CuI_2N_2	1	Tetrahedral	4,4'-bipyridine
[Cu_2I_2(bpy)]	2D	$CuIN_3$	1	Tetrahedral	4,4'-bipyridine
[Cu_2I_2(pyz)]	2D	$CuIN_3$	1	Tetrahedral	pyrazine
[$Cu_4I_4(DABCO)_2$]	3D	$CuIN_3$	3	Tetrahedral	1,4-diazabicyclo[2.2.2]octane
{[CuI][Cu(pdc)(H_2O)]·1.5MeCN·H_2O}$_n$	3D	$CuIN_3$	1	Tetrahedral	pyridine-3,5-dicarboxylic acid
Cu_2BDC	2D	CuO_4	1	Seesaw	1,4-benzenedicarboxylic acid
Cu(bpy)$_{1.5}NO_3$·1.25H_2O	3D	CuN_3	1	Trigonal planer	4,4'-bipyridine
Cu_3(4hypymca)$_3$	2D	CuN_3	1	Trigonal planar	4-hydroxypyrimidine-5-carbonitrile
SLUG-22	1D	CuN_2	2	Linear	4,4'-bipyridine
[$Cu_6I_6(DABCO)_2$]	3D	CuI_2, CuI_4, CuI_2N_2	4	Tetrahedral and linear	1,4-diazabicyclo[2.2.2]octane

Table S2. ^{65}Cu static solid echo NMR experimental parameters at 21.1 T.

Sample	π/2 ^{65}Cu pulse width (μs)	Recycle delay (s)	Step size (kHz)	Number of scans	Number of pieces[a]
[CuCl(bpy)]	1.0	0.2	300	65536	3
[Cu_2Cl_2(bpy)]	1.0	0.2	300	65536	3
[CuI(bpy)]	1.0	0.2	300	32768	3
[Cu_2I_2(bpy)]	1.0	0.2	N/A	65536	1
[Cu_2I_2(pyz)]	1.0	0.2	N/A	32768	1
[$Cu_4I_4(DABCO)_2$]	1.0	0.2	300	16384	3
Cu(bpy)$_{1.5}NO_3$· 1.25H_2O	1.0	0.2	600	32768	6
Cu_3(4hypymca)$_3$	1.0	0.2	600	32768	9
SLUG-22	1.0	0.2	600	32768	6
{[CuI][Cu(pdc)(H_2O)]·1.5 MeCN·H_2O}$_n$	1.0	0.2	300	32768	3
Cu_2BDC	1.0	0.2	600	32768	5

[a] The "number of pieces" column refers to the number of frequency-stepped subspectra that were acquired in order to assemble the overall ultra-wideline spectrum using the variable-offset cumulative spectra (VOCS)[12] protocol.

Table S3. ^{63}Cu static solid echo NMR experimental parameters used at 21.1 T.

Sample	π/2 ^{63}Cu pulse width (μs)	Recycle delay (s)	Step size (kHz)	Number of scans	Number of pieces[a]
[CuCl(bpy)]	1.0	0.2	300	32768	4
[Cu_2Cl_2(bpy)]	1.0	0.2	200	32768	4
[CuI(bpy)]	1.0	0.2	300	32768	4
[Cu_2I_2(bpy)]	1.0	0.2	200	16384	3
[Cu_2I_2(pyz)]	1.0	0.2	200	16384	3
[$Cu_4I_4(DABCO)_2$]	1.0	0.2	200	8192	3
[$Cu_6I_6(DABCO)_2$]	1.0	0.2	200	8192	3
$Cu(bpy)_{1.5}NO_3\cdot 1.25H_2O$	1.0	0.2	600	32768	9
$Cu_3(4hypymca)_3$	1.0	0.2	600	32768	10
SLUG-22	1.0	0.2	300	16384	14
$\{[CuI][Cu(pdc)(H_2O)]\cdot 1.5 MeCN\cdot H_2O\}_n$	1.0	0.2	300	32768	3
Cu_2BDC	1.0	0.2	600	32768	6

[a] The "number of pieces" column refers to the number of frequency-stepped subspectra that were acquired in order to assemble the overall ultra-wideline spectrum using the variable-offset cumulative spectra (VOCS)[12] protocol.

[b] To minimize the ^{23}Na background signal originating from glass sample tubes, all samples were packed in a 5 mm Teflon tube. An example of a ^{63}Cu NMR spectrum collected at 21.1 T using a glass sample holder versus a Teflon tube is provided in Figure S3(a,b). Despite the use of a Teflon sample holder, interference from the probe background (*i.e.*, elements present in the NMR probe and circuits) is present in some spectra. An NMR spectrum of the probe background signal when tuned to the ^{63}Cu frequency at 21.1 T is shown in Figure S3(c). All $^{63/65}$Cu NMR experiments were performed at room temperature using a dry nitrogen purge.

Table S4. ^{65}Cu static WURST-CPMG NMR experimental parameters at 9.4 T.

Sample	Spectral width (kHz)	Recycle delay (s)	Step size (kHz)	Number of scans	Number of pieces[a]	Experimental time[b] (h)
[CuCl(bpy)]	2000	0.4	300	32768	3	10.9
[Cu_2Cl_2(bpy)]	1000	0.4	300	16384	2	3.6
[CuI(bpy)]	2000	0.4	300	8192	3	2.7
[Cu_2I_2(bpy)]	1000	0.4	300	8192	2	1.8
[Cu_2I_2(pyz)]	1000	0.4	N/A	4096	1	0.5
[Cu_4I_4(DABCO)$_2$]	2000	0.4	300	16384	3	5.5
Cu_2BDC	2000	0.2	250	8192	15	6.8

[a] The "number of pieces" column refers to the number of frequency-stepped subspectra that were acquired in order to assemble the overall ultra-wideline spectrum using the variable-offset cumulative spectra (VOCS)[12] protocol. The spectral width column refers to the spectral width used in the acquisition of each individual "piece" or sub-spectrum.

[b] The listed experimental time does not include the time required to change the transmitter frequency offset and tune the probe between the acquisition of each individual sub-spectrum.

Table S5. Experimental and calculated $^{63/65}Cu$ NMR parameters in MOFs with cluster models.

Site	Method	Calculation method/basis set	$C_Q(^{65}Cu)$ (MHz)	$C_Q(^{63}Cu)$ (MHz)	η_Q	δ_{iso} or σ_{iso} (ppm)	Ω (ppm)	κ	α (°)	β (°)	γ (°)
						**[CuCl(bpy)]**					
Cu1	Exp.		30.0(4)	33.5(4)	0.45(3)	500(50)	600(200)	0.4(1)	10(3)	28(3)	35(3)
Cu1	Calc.	RHF/6-31++G**	28.6	30.8	0.23	1284.8	357.5	0.4	232.4	4.2	74.5
		RHF/6-311++G**	41.6	44.9	0.24	1194.9	354.7	0.5	330.6	3.2	103.7
		B3LYP/6-31++G**	14.5	15.7	0.42	146.2	1152.9	0.3	110.2	38.7	237.4
		B3LYP/6-311++G**	21.3	23.0	0.26	-30.1	1224.0	0.3	228.9	37.3	303.8
		RPBE/TZ2P	24.1	26.0	0.67	-1647.8	2863.3	0.4	240.8	64.4	131.8
		PBE0/TZ2P	22.9	24.7	0.26	-27.9	1242.1	0.3	319.7	37.0	57.1
		CASTEP	34.1	36.8	0.41	2188.7	2402.7	-0.2	27.3	60.4	-134.0
						**[CuI(bpy)]**					
Cu1	Exp.		28.7(3)	30.2(4)	0.50(4)	400(100)	300(200)	1.0(1)	90(2)	35(2)	10(2)
Cu1	Calc.	RHF/6-31++G**	25.3	27.3	0.09	1238.3	101.8	-0.1	315.0	7.1	96.6
		RHF/6-311++G**	35.9	38.7	0.20	1155.3	90.5	-0.2	295.0	13.2	101.7
		B3LYP/6-31++G**	14.1	15.2	0.38	417.9	445.0	0.5	309.8	39.7	29.3
		B3LYP/6-311++G**	19.2	20.7	0.38	255.0	477.5	0.5	34.3	39.4	148.6
		RPBE/TZ2P	19.7	21.3	0.62	-898.9	1587.6	0.5	308.2	62.0	47.3
		PBE0/TZ2P	21.23	22.9	0.43	275.8	509.5	0.487	28.46	38.5	149.4
		CASTEP	28.6	30.8	0.45	1215.0	806.2	-0.2	34.3	45.0	-172.4
						**[Cu_2I_2(bpy)]**					

Cu1	Exp.		24.0(4)	26.0(5)	0.18(3)	280(50)	400(200)	-1.0(3)	0(3)	25(2)	65(5)
Cu1	Calc.	RHF/6-31++G**	10.9	11.7	0.65	1363.2	252.3	0.3	16.7	85.7	56.1
		RHF/6-311++G**	17.0	18.3	0.67	1271.3	251.8	0.4	14.4	85.6	57.6
		B3LYP/6-31++G**	25.7	27.7	0.53	-423.7	1996.8	-0.4	189.8	25.4	241.6
		B3LYP/6-311++G**	33.7	36.3	0.46	-692.7	2238.5	-0.4	184.7	27.4	242.0
		RPBE/TZ2P	51.5	55.5	0.57	-3303.6	6846.3	-0.3	359.6	22.3	307.7
		PBE0/TZ2P	33.9	36.6	0.48	-900.5	2575.4	-0.5	189.3	25.2	242.9
		CASTEP	25.5	27.5	0.47	867.3	741.4	0.1	23.4	20.0	-57.2
**[Cu_2Cl_2(bpy)]**											
Cu1	Exp.		30.0(3)	32.0(5)	0.25(2)	230(30)	500(100)	0.1(2)	0(2)	25(3)	58(2)
Cu1	Calc.	RHF/6-31++G**	14.3	15.4	0.34	1359.2	382.0	0.5	172.8	2.9	241.5
		RHF/6-311++G**	23.8	25.7	0.27	1269.3	402.3	0.5	287.3	2.3	132.9
		B3LYP/6-31++G**	37.1	40.0	0.55	-3773.5	8926.4	0.1	151	13.1	286.4
		B3LYP/6-311++G**	43.4	46.8	0.51	-4154.3	9424.1	0.1	149.1	16.6	284.3
		RPBE/TZ2P	57.6	62.1	0.52	-9197.4	21367.6	0.1	245.1	14.8	78.3
		PBE0/TZ2P	47.8	51.6	0.53	-5414.0	12013.3	0.0	329.5	16.3	103.3
		CASTEP	27.6	29.4	0.80	1070.0	906.5	-0.7	103.1	89.5	92.8
**[Cu_2I_2(pyz)]**											
Cu1	Exp.		18.8(4)	19.6(5)	0.35(2)	300(50)	480(50)	-0.8(2)	10(3)	25(2)	60(4)
Cu1	Calc.	RHF/6-31++G**	9.2	9.9	0.65	1356.5	228.1	0.0	13.2	77.7	58.7
		RHF/6-311++G**	14.7	15.8	0.65	1264.6	227.9	0.1	12.6	78.7	56.8
		B3LYP/6-31++G**	18.0	19.3	0.91	-97.6	1244.6	-0.9	359.2	66.2	98.1
		B3LYP/6-311++G**	24.1	25.9	0.76	-361.5	1400.4	-0.9	356.6	52.0	99.8

		RPBE/TZ2P	37.5	40.5	0.87	-2446.4	4270.5	-0.9	186.9	36.0	258.2
		PBE0/TZ2P	24.5	26.5	0.72	-350.3	1471.8	-0.9	179.8	67.2	98.8
		CASTEP	18.2	19.6	0.53	3701.7	2585.8	-0.5	-53.5	4.6	43.5
						**[Cu_4I_4(DABCO)]**					
Cu1	Exp.		22.1(5)	23.8(3)	0.09(3)	320(40)	250(75)	1.0(4)	0	0	0
Cu1	Calc.	RHF/6-31++G**	22.3	24.0	0.11	1252.7	113.9	0.9	270.0	14.0	180.0
		RHF/6-311++G**	31.0	33.4	0.10	1155.1	110.4	0.8	270.0	17.8	180.0
		B3LYP/6-31++G**	14.4	15.6	0.18	647.6	154.7	0.6	0.0	15.1	0.0
		B3LYP/6-311++G**	20.3	22.0	0.17	490.0	160.7	0.6	0.0	14.8	0.0
		RPBE/TZ2P	20.1	21.6	0.20	139.1	309.4	0.7	180.1	13.5	359.9
		PBE0/TZ2P	22.8	24.5	0.25	485.7	420.7	-0.2	356.8	26.2	93.5
		CASTEP	16.4	17.7	0.22	-58.3	335.6	-0.6	-90.0	12.5	-180.0
Cu2	Exp.		20.6(3)	22.0(4)	0.14(4)	280(20)	280(50)	1.0(3)	0.0	0.0	0.0
Cu2	Calc.	RHF/6-31++G**	18.5	20.0	0.37	1228.0	255.8	-0.1	171.2	37.2	283.7
		RHF/6-311++G**	26.4	28.5	0.34	1130.7	251.0	-0.1	350.9	41.0	284.9
		B3LYP/6-31++G**	13.0	14.0	0.31	639.3	386.5	-0.2	178.1	26.5	271.6
		B3LYP/6-311++G**	19.0	20.5	0.28	481.4	390.4	-0.2	174.0	28.6	274.3
		RPBE/TZ2P	18.1	19.5	0.22	112.0	540.4	-0.2	4.1	18.0	263.5
		PBE0/TZ2P	24.0	25.9	0.13	495.8	181.5	0.7	0.0	13.0	0.4
		CASTEP	15.3	16.5	0.37	-90.4	589.8	0.2	7.4	85.8	-160.5
Cu3	Exp.		26.7(6)	29.1(5)	0.03(3)	320(40)	200(50)	1.0(3)	0	0	0
Cu3	Calc.	RHF/6-31++G**	19.8	18.4	0.00	1223.5	38.7	0.6	0.0	1.9	0.0
		RHF/6-311++G**	25.8	27.8	0.01	1125.0	28.7	0.3	0.0	1.0	0.0

		B3LYP/6-31++G**	16.8	18.2	0.02	662.0	174.4	0.9	180.0	1.3	270.0
		B3LYP/6-311++G**	23.6	25.4	0.02	505.6	173.9	0.9	180.0	0.9	270.0
		RPBE/TZ2P	23.0	24.8	0.07	138.4	274.3	1.0	179.8	8.7	88.3
		PBE0/TZ2P	27.5	29.7	0.02	510.5	191.0	0.9	182.4	1.2	88.5
		CASTEP	23.0	24.8	0.02	-58.8	265.7	-0.9	90.0	5.4	-90.0
						**{[CuI][Cu(pdc)(H_2O)]·1.5MeCN·H_2O}$_n$**					
Cu1	Exp.		22.0(3)	24.0(2)	0.02(2)	400(15)	150(100)	1.0(2)	0	0	0
Cu1	Calc.	RHF/6-31++G**	10.2	10.9	0.37	1423.0	139.9	-0.8	265.9	88.3	320.9
		RHF/6-311++G**	16.7	15.5	0.18	1334.2	150.2	-0.9	184.3	53.1	355.7
		B3LYP/6-31++G**	10.9	11.7	0.16	238.2	1597.4	0.1	56.3	8.1	21.3
		B3LYP/6-311++G**	14.2	15.3	0.08	35.3	1814.3	0.1	45.1	10.5	36.2
		RPBE/TZ2P	22.4	24.2	0.33	-1813.0	6452.1	-0.5	36.9	29.0	253.3
		PBE0/TZ2P	14.9	16.2	0.15	14.7	1705.0	0.3	342.6	17.1	117.0
		CASTEP	23.2	25.0	0.02	970.0	441.2	-0.9	0.0	15.9	90.0
						**Cu_2BDC**					
Cu1	Exp.		53.0(3)	57.0(4)	0.22(3)	200(150)	1800(300)	1.0(4)	0	0	0
Cu1	Calc.	UHF/6-31++G**	49.3	53.1	0.82	285.2	1325.2	0.44	8.3	88.3	202.9
		UHF/6-311++G**	37.8	40.7	0.12	-215.2	2365.3	0.1	27.1	70.9	74.3
		B3LYP/6-31++G**	73.5	79.3	0.42	-720.6	6891.4	0.7	167.4	68.2	15.7
		B3LYP/6-311++G**	40.8	44.0	0.91	-2101.1	7017.9	-0.2	57.0	52.1	188.8
		RPBE/TZ2P	78.8	85.0	0.91	-2397.9	3598.5	0.1	203.5	7.5	56.1
		PBE0/TZ2P	34.7	37.4	0.44	-6489.7	21556.0	-0.7	93.4	82.0	331.6
		CASTEP	57.5	62.0	0.17	3713.6	6569.0	0.2	158.7	2.2	25.4

					**$Cu(bpy)_{1.5}NO_3 \cdot 1.25H_2O$**						
Cu1	Exp.		74.0(4)	79.0(6)	0.18(2)	300(100)	0	0	0	0	0
Cu1	Calc.	RHF/6-31++G**	79.7	85.9	0.34	1249.5	947.9	0.2	95.5	88.8	348.9
		RHF/6-311++G**	108.5	117.0	0.41	1165.0	988.6	0.2	94.9	88.8	349.9
		B3LYP/6-31++G**	61.7	66.6	0.03	555.1	1261.1	0.2	91.3	89.7	355.2
		B3LYP/6-311++G**	83.1	89.6	0.13	413.0	1343.3	0.2	95.8	89.6	355.8
		RPBE/TZ2P	74.2	80.0	0.14	-193.5	1669.9	-0.1	87.4	89.7	179.3
		PBE0/TZ2P	86.9	93.6	0.19	453.1	1334.6	0.1	86.2	89.4	183.7
		CASTEP	74.5	80.3	0.17	1337.3	2229.1	0.2	-40.8	1.3	39.0
					**$Cu_3(4hypymca)_3$**						
Cu1	Exp.		74.8(6)	80.6(4)	0.55(2)	150(200)	0	0	0	0	0
Cu1	Calc.	RHF/6-31++G**	58.7	63.3	0.63	1372.7	961.5	-0.0	267.4	90.0	0.0
		RHF/6-311++G**	86.2	92.9	0.65	1261.8	959.4	0.0	267.7	90.0	0.0
		B3LYP/6-31++G**	50.5	54.5	0.17	801.5	1077.7	0.0	258.6	90.0	0.0
		B3LYP/6-311++G**	72.0	77.6	0.27	655.8	1112.6	0.1	262.7	90.0	0.0
		RPBE/TZ2P	74.4	80.3	0.14	230.3	1042.0	0.2	103.1	90.0	0.0
		PBE0/TZ2P	76.9	83.0	0.30	697.5	1085.6	0.1	95.9	90.0	0.0
		CASTEP	95.6	103.1	0.11	786.4	744.3	0.2	98.5	180.0	-136.0
					**SLUG-22**						
	Exp.		63.0(10)	67.0(8)	0.34(2)	100(150)	1500(200)	1.0(1)	0	0	0
Cu1	Calc.	RHF/6-31++G**	62.5	67.4	0.36	1435.6	1424.0	1.0	19.0	0.6	61.1
		RHF/6-311++G**	99.8	107.7	0.24	1347.2	1492.0	0.9	35.9	0.5	48.4
		B3LYP/6-31++G**	27.5	29.6	0.54	1049.6	1860.8	0.9	29.4	1.4	56.1

		B3LYP/6-311++G**	50.7	54.7	0.28	927.3	1983.0	0.9	37.9	1.1	47.6
		RPBE/TZ2P	43.4	46.8	0.10	674.1	2168.2	0.8	138.0	1.4	318.3
		PBE0/TZ2P	58.7	63.3	0.21	932.7	1961.5	0.8	135.9	1.0	318.4
		CASTEP	40.0	44.2	0.74	786.5	3181.5	-0.2	174.4	172.4	53.0
Cu2	Calc.	RHF/6-31++G**	62.5	67.4	0.36	1435.7	1423.8	1.0	19.0	0.6	61.2
		RHF/6-311++G**	99.8	107.7	0.24	1347.3	1491.9	0.9	35.9	0.5	48.4
		B3LYP/6-31++G**	27.5	29.6	0.54	1049.7	1860.7	0.9	29.3	1.4	56.2
		B3LYP/6-311++G**	50.7	54.7	0.28	927.4	1982.9	0.9	38.0	1.1	47.6
		RPBE/TZ2P	44.5	47.4	0.12	674.2	2163.0	0.8	138.5	1.4	318.3
		PBE0/TZ2P	60.1	64.2	0.22	932.7	1964.0	0.8	135.9	0.9	318.4
		CASTEP	42.2	45.5	0.86	859.8	3055.1	-0.2	-17.3	3.2	-22.3
						**$[Cu_6I_6(DABCO)_2]$**					
Cu1,2,3	Exp.		19.1(3)	21.4(3)	0.70(2)	670(20)	0	0	0	0	0
Cu1	Calc.	RHF/6-31++G**	22.2	23.9	0.59	1316.6	216.6	-0.5	277.5	24.5	187.3
		RHF/6-311++G**	31.3	33.7	0.54	1225.6	237.2	-0.6	75.4	23.0	355.8
		B3LYP/6-31++G**	16.1	17.4	0.71	807.6	479.7	0.2	17.9	31.4	209.2
		B3LYP/6-311++G**	22.9	24.7	0.72	666.6	520.6	0.2	170.5	30.6	324.4
		RPBE/TZ2P	20.4	22.0	0.76	349.9	674.0	0.1	171.3	32.3	329.0
		PBE0/TZ2P	25.6	27.6	0.66	680.1	533.8	0.2	188.8	30.5	218.8
		CASTEP	19.2	20.7	0.54	81.3	709.4	0.5	-28.6	125.2	175.7
Cu2	Calc.	RHF/6-31++G**	24.3	26.2	0.60	1335.0	298.1	0.2	15.1	28.7	233.2
		RHF/6-311++G**	34.0	36.6	0.61	1244.5	321.2	0.2	171.1	29.1	303.5
		B3LYP/6-31++G**	15.3	16.6	0.48	780.1	403.9	-0.5	78.2	36.2	162.0

		B3LYP/6-311++G**	21.6	23.4	0.47	635.8	439.5	-0.5	248.7	33.0	345.7
		RPBE/TZ2P	20.2	21.7	0.45	312.2	611.8	-0.5	304.2	36.5	190.7
		PBE0/TZ2P	24.6	26.5	0.44	648.9	454.4	-0.5	294.1	32.1	190.0
		CASTEP	19.3	20.8	0.25	124.5	724.9	-0.2	96.3	21.1	-143.4
Cu3	Calc.	RHF/6-31++G**	17.7	19.1	0.54	1433.4	262.8	-0.0	350.5	31.8	97.7
		RHF/6-311++G**	26.1	28.2	0.51	1353.7	266.7	-0.0	352.5	35.3	98.8
		B3LYP/6-31++G**	8.9	9.6	0.78	957.5	423.4	-0.0	351.5	16.3	86.7
		B3LYP/6-311++G**	13.7	14.8	0.71	827.5	433.4	-0.1	352.8	19.4	89.4
		RPBE/TZ2P	17.5	18.88	0.84	497.3	842.0	0.88	179.4	1.04	270.8
		PBE0/TZ2P	27.0	29.1	0.54	862.4	847.0	0.9	1.3	0.5	267.9
		CASTEP	7.2	7.8	0.34	324.5	704.7	0.5	0	104.3	0
Cu4	Exp.		24.1(2)	27.0(2)	0.20(3)	280(50)	0	0	0	0	0
Cu4	Calc.	RHF/6-31++G**	39.5	42.6	0.21	1509.1	680.3	1.0	359.9	0.2	0.0
		RHF/6-311++G**	53.2	57.4	0.26	1429.2	728.0	1.0	180.4	0.1	179.5
		B3LYP/6-31++G**	19.1	20.6	0.46	985.4	750.2	1.0	179.9	0.5	270.0
		B3LYP/6-311++G**	26.8	28.9	0.57	854.3	816.5	1.0	179.9	0.7	180.5
		RPBE/TZ2P	20.1	21.7	0.45	312.1	610.9	-0.5	55.7	36.6	169.1
		PBE0/TZ2P	24.5	26.4	0.44	648.9	453.4	-0.5	66.2	32.1	169.6
		CASTEP	24.1	26.0	0.24	257.0	868.6	-0.9	-90.0	0.2	90.0
						**$Cu_2(pyz)_2(SO_4)(H_2O)_2$**					
Cu1	Exp.		25.2(2)	27.2(4)	0.54(2)	500(100)	900(100)	0.0	70(2)	-4(2)	-11(3)
Cu1	Calc.	RHF/6-31++G**	30.7	33.1	0.55	1309.9	664.9	0.2	82.0	86.4	2.6
		RHF/6-311++G**	43.0	46.4	0.61	1222.4	677.1	0.1	82.7	86.5	2.9

B3LYP/6-31++G**	10.0	10.7	0.18	-245.8	2197.3	-0.5	224.0	52.4	115.9
B3LYP/6-311++G**	15.2	16.3	0.33	-443.6	2339.9	-0.5	341.6	48.2	70.2
RPBE/TZ2P	18.1	19.5	0.75	-1192.7	2416.3	-0.3	306.6	46.6	256.3
PBE0/TZ2P	17.0	18.4	0.25	-451.7	2427.6	-0.6	345.2	47.7	70.3
CASTEP	23.7	25.5	0.55	2079.4	2995.0	-0.4	43.5	58.0	-73.0

Table S6. Cu NMR parameters extracted from spectra obtained at 9.4 T and 21.1 T. The $C_Q(^{63}Cu)$ value at 9.4 T was obtained by scaling the $C_Q(^{65}Cu)$ value using the quadrupolar moment ratio of 1.078.[30]

Site	Field	$C_Q(^{65}Cu)$ (MHz)	$C_Q(^{63}Cu)$ (MHz)	η_Q	δ_{iso} (ppm)	Ω (ppm)	κ	α (°)	β (°)	γ (°)
					**[CuCl(bpy)]**					
1	21.1 T	30.0(4)	33.5(4)	0.45(3)	500(50)	600(200)	0.4(1)	10(3)	28(3)	35(3)
1	9.4 T	30.4(8)	33.9(6)	0.44(5)	500(100)	600(450)	0.4(3)	-	-	-
					**[Cu_2I_2(pyz)]**					
1	21.1 T	18.8(4)	19.6(5)	0.35(2)	300(50)	480(50)	-0.8(2)	10(3)	25(2)	60(4)
1	9.4 T	18.8(6)	19.6(7)	0.35(5)	300(100)	480(150)	-0.8(4)	-	-	-
					**[Cu_4I_4(DABCO)]**					
1	21.1 T	22.1(5)	23.8(3)	0.09(3)	320(40)	250(75)	1.0(4)	-	-	-
1	9.4 T	22.1(8)	23.8(8)	0.09(6)	320(80)	250(120)	1.0(8)	-	-	-
2	21.1 T	20.6(3)	22.0(4)	0.14(4)	280(20)	280(50)	1.0(3)	-	-	-
2	9.4 T	20.2(6)	21.8(6)	0.14(7)	280(100)	280(120)	1.0(6)			
3	21.1 T	26.7(6)	29.1(5)	0.03(4)	320(40)	200(50)	1.0(3)	-	-	-
3	9.4 T	26.7(10)	29.1(10)	0.03(6)	320(100)	200(120)	1.0(6)			
					**[Cu_2I_2(bpy)]**					
1	21.1 T	24.0(4)	26.0(5)	0.18(3)	280(50)	400(200)	-1.0(3)	0(3)	25(2)	65(5)
1	9.4 T	24.5(10)	26.5(10)	0.18(8)	280(100)	400(350)	-1.0(8)			
					**[CuI(bpy)]**					
1	21.1 T	28.7(3)	30.2(4)	0.50(4)	400(100)	300(200)	1.0(1)	90(2)	35(2)	10(2)
1	9.4 T	28.7(10)	30.2(10)	0.50(8)	400(200)	300(300)	1.0(4)	-	-	-
					**Cu_2BDC**					
1	21.1 T	53.0(3)	57.0(4)	0.22(3)	200(150)	1800(300)	1.0(4)	-	-	-
1	9.4 T	53.0(10)	57.0(10)	0.22(8)	200(250)	1800(700)	1.0(5)	-	-	-

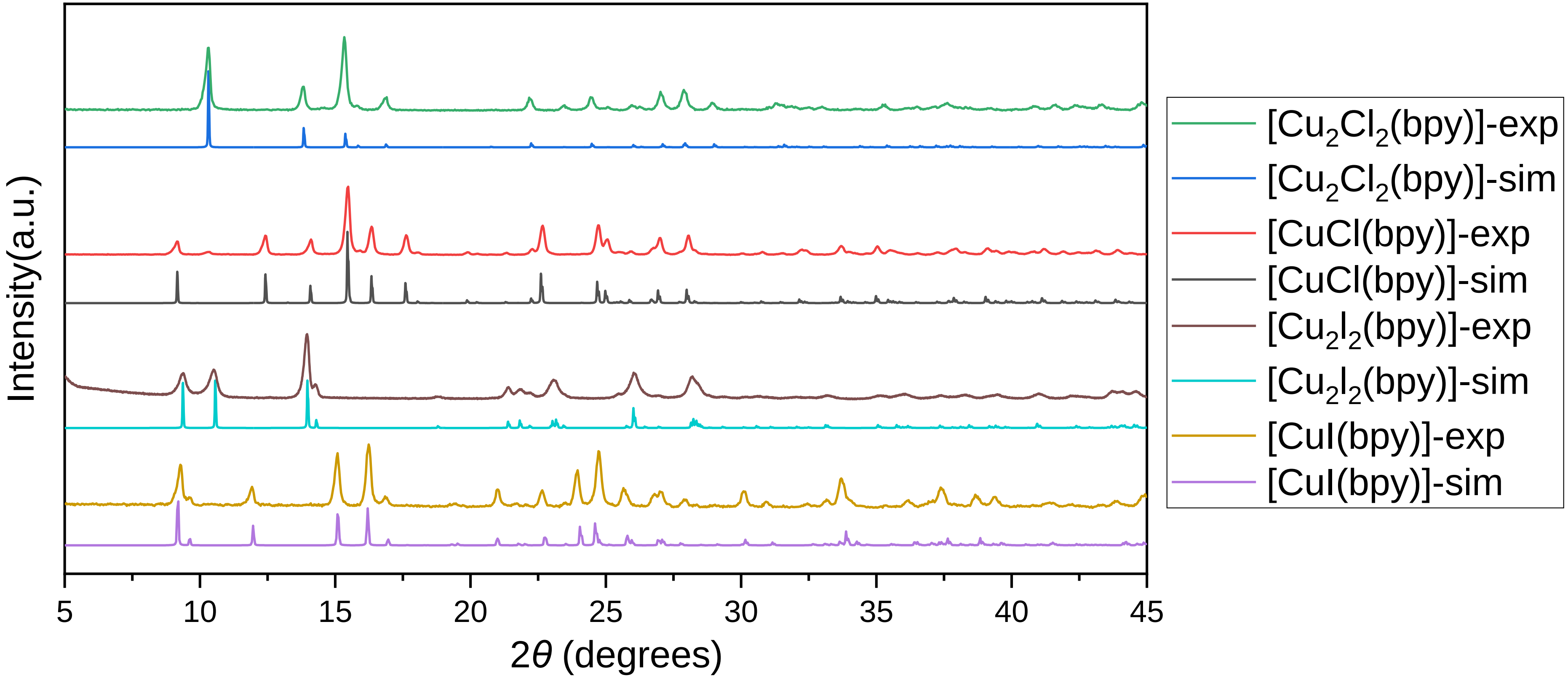

Figure S1. Experimental (exp) and simulated (sim) PXRD patterns of the [CuCl(bpy)], [Cu_2Cl_2(bpy)], [CuI(bpy)] and [Cu_2I_2(bpy)] MOFs.

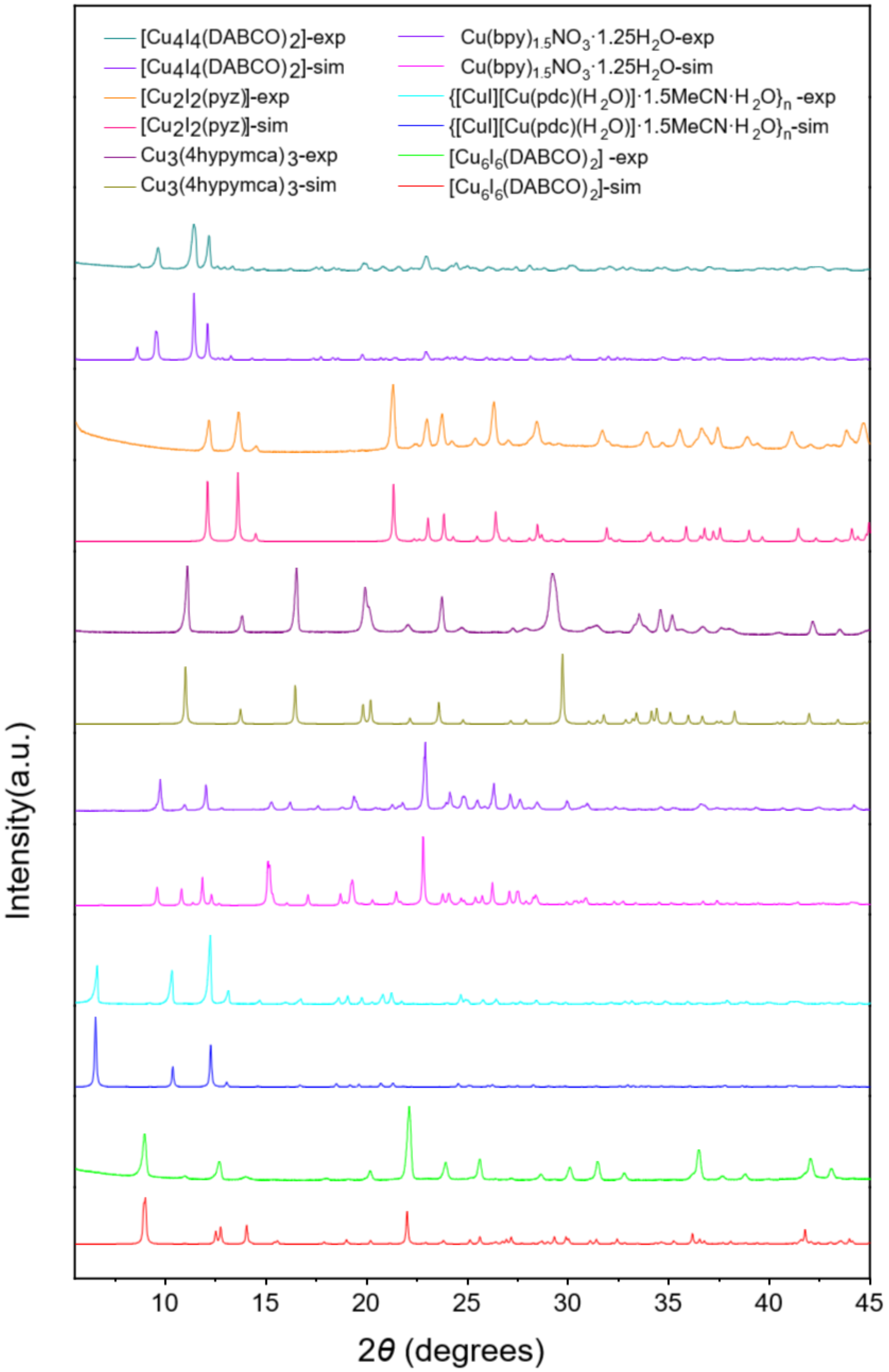

Figure S2. Experimental (exp) and simulated (sim) PXRD patterns of the Cu(I) MOFs examined in this work.

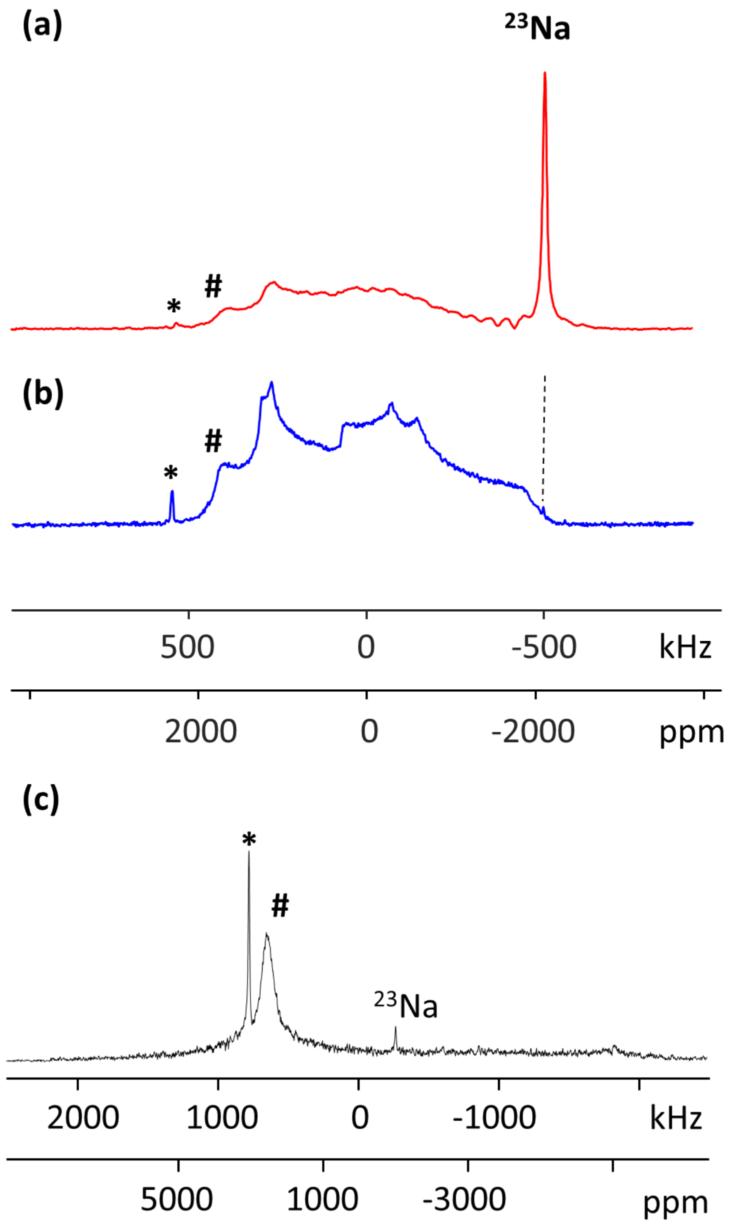

Figure S3. ^{63}Cu NMR spectra of CuCl(bpy) that were obtained when the sample was packed in (a) glass and (b) Teflon tubes as sample holders. Note the lack of a strong ^{23}Na background signal from the Teflon tube. The asterisks (*) denote a signal from metallic copper (Cu(0)) and the pound (#) marks a signal from probe background. (c) The ^{63}Cu NMR spectrum of probe background is shown, as obtained using an empty probe.

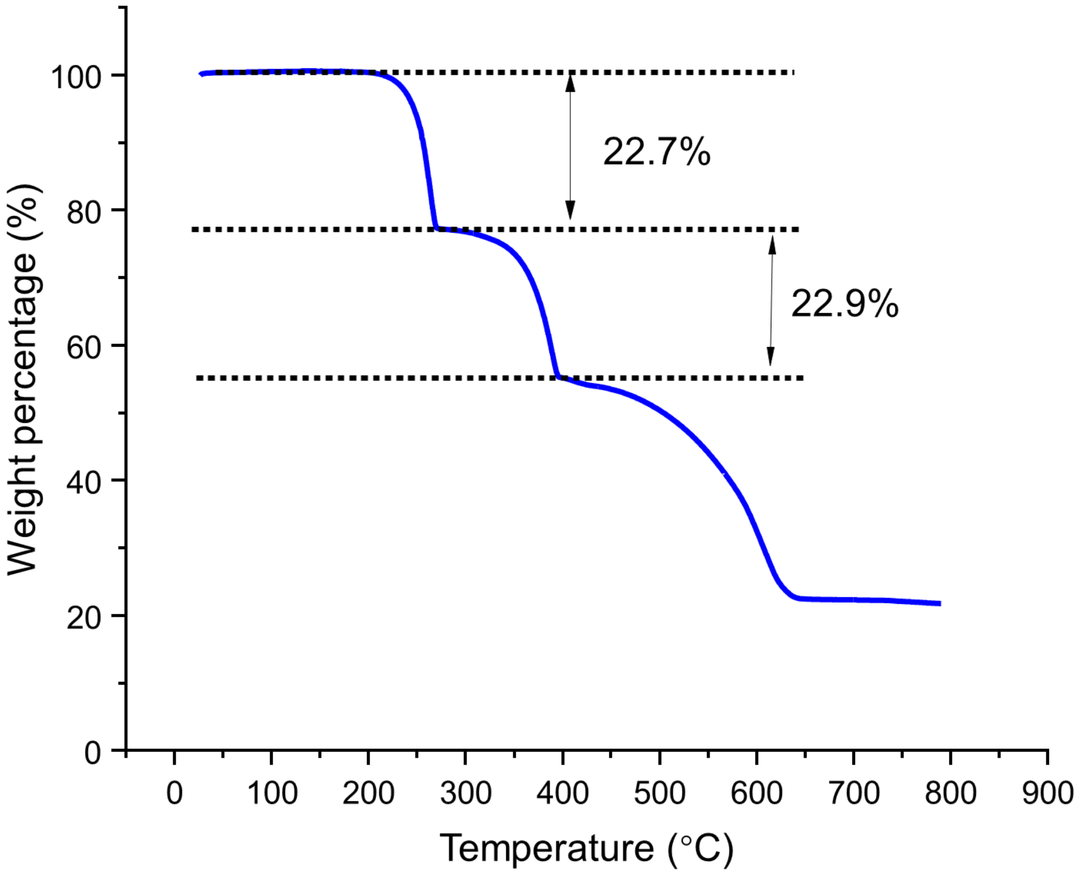

Figure S4. The TGA curve of [CuI(bpy)], as measured under nitrogen atmosphere, is shown. The initial weight loss of 22.7 % corresponds to removal of the MOF linker according to 2[CuI(bpy)] → [Cu_2I_2(bpy)] + bpy↑, which has a calculated weight loss of 22.5 %. The second stage weight loss of 22.9 % corresponds to the collapse of the framework and loss of the remaining linkers.

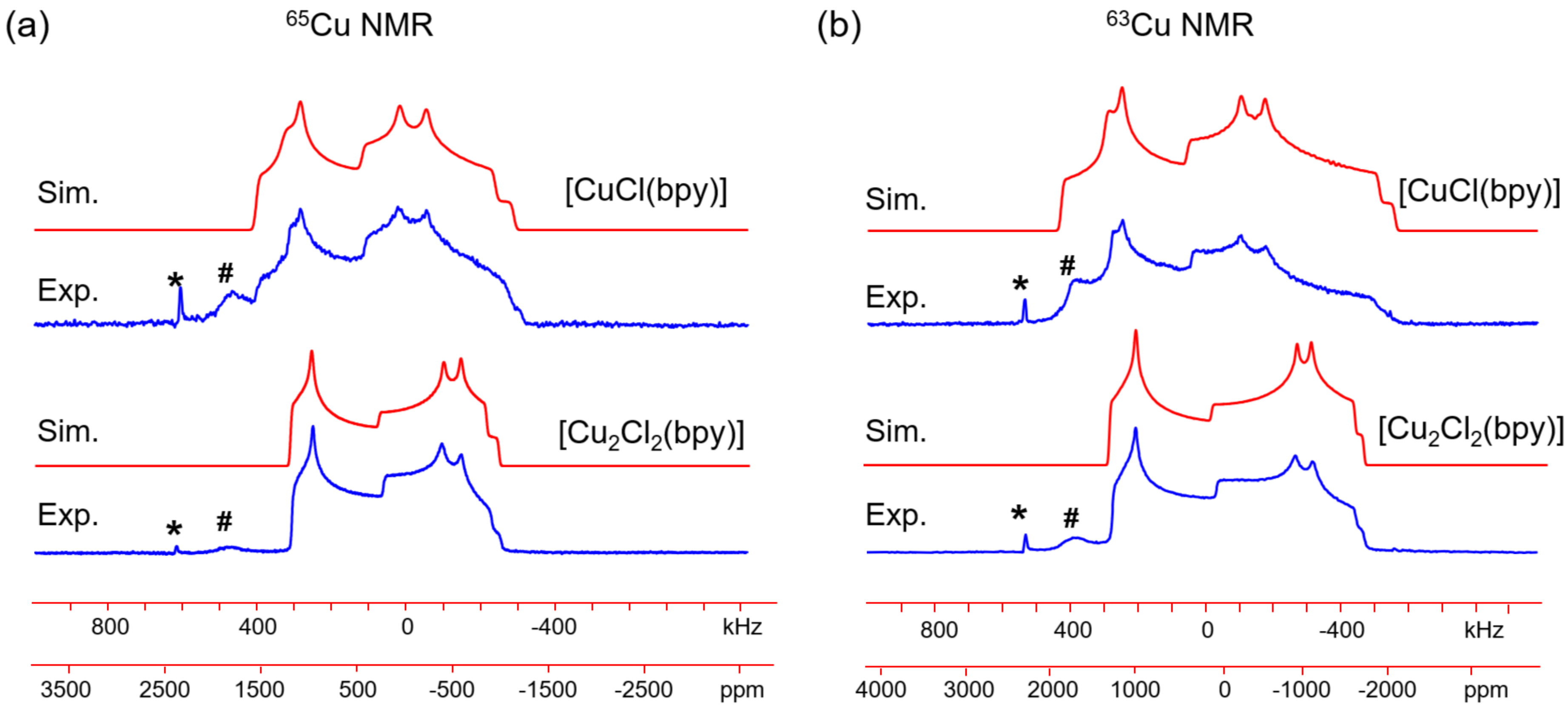

Figure S5. Experimental (blue) and simulated (red) $^{63/65}$Cu static solid echo NMR spectra of [CuCl(bpy)] and [Cu_2Cl_2(bpy)] at 21.1 T. The asterisk (*) denotes a signal from metallic copper (Cu(0)) and the pound (#) marks a signal from probe background.

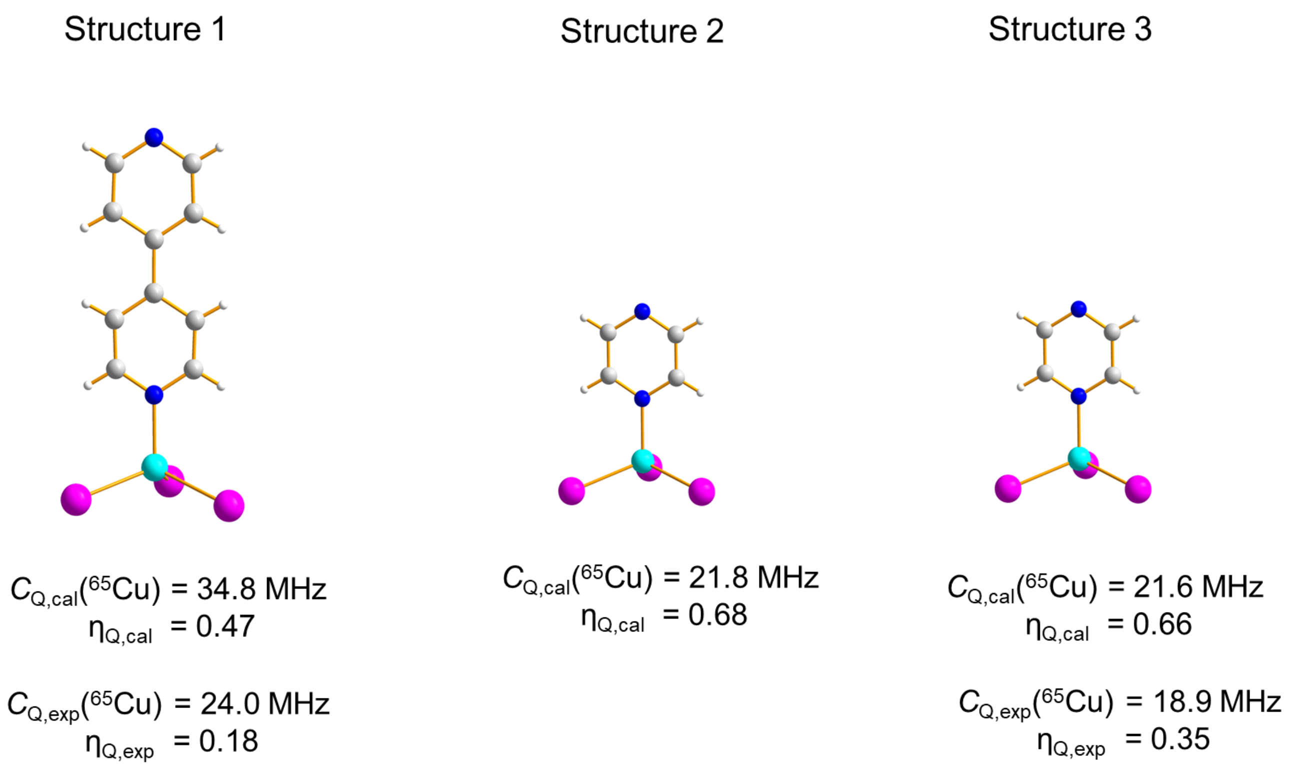

Figure S6. The models used for DFT calculations on distinct molecules. Structures 1 and 3 were taken from the crystal structures of [Cu_2I_2(bpy)] and [Cu_2I_2(pyz)].[3,15] Structure 2 was constructed by manually replacing the 4,4'-bipyridine linker with the pyrazine ligand coordinates taken from structure 3. The V_{33} component of the EFG tensor, which is proportionate to C_Q, is oriented along or nearly along the Cu-N bond.

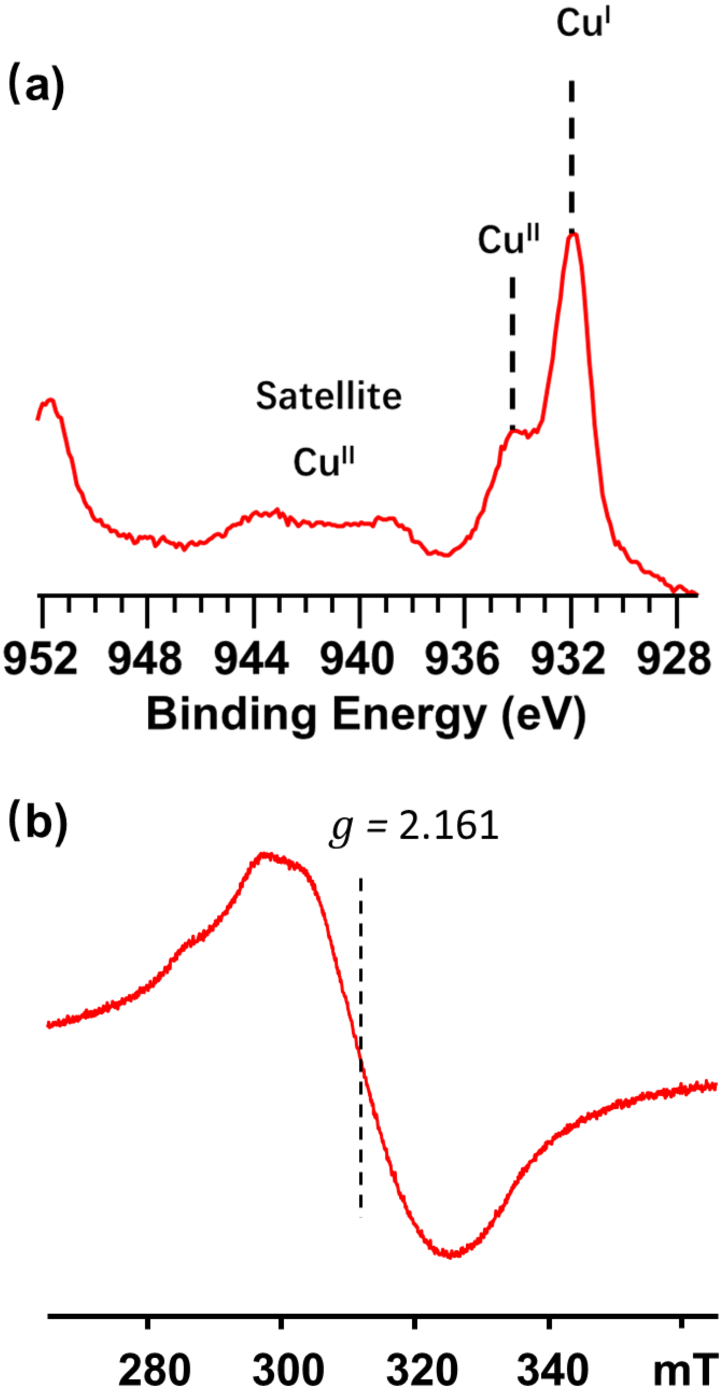

Figure S7. (a) The Cu $2p_{3/2}$ XPS spectrum of $\{[Cu(I)][Cu(II)(pdc)(H_2O)]\cdot 1.5MeCN\cdot H_2O\}_n$, along with peak assignments in black. (b) the X-band EPR spectrum of $\{[Cu(I)][Cu(II)(pdc)(H_2O)]\cdot 1.5MeCN\cdot H_2O\}_n$ MOF at 298 K, with the g-value of 2.161 indicated.

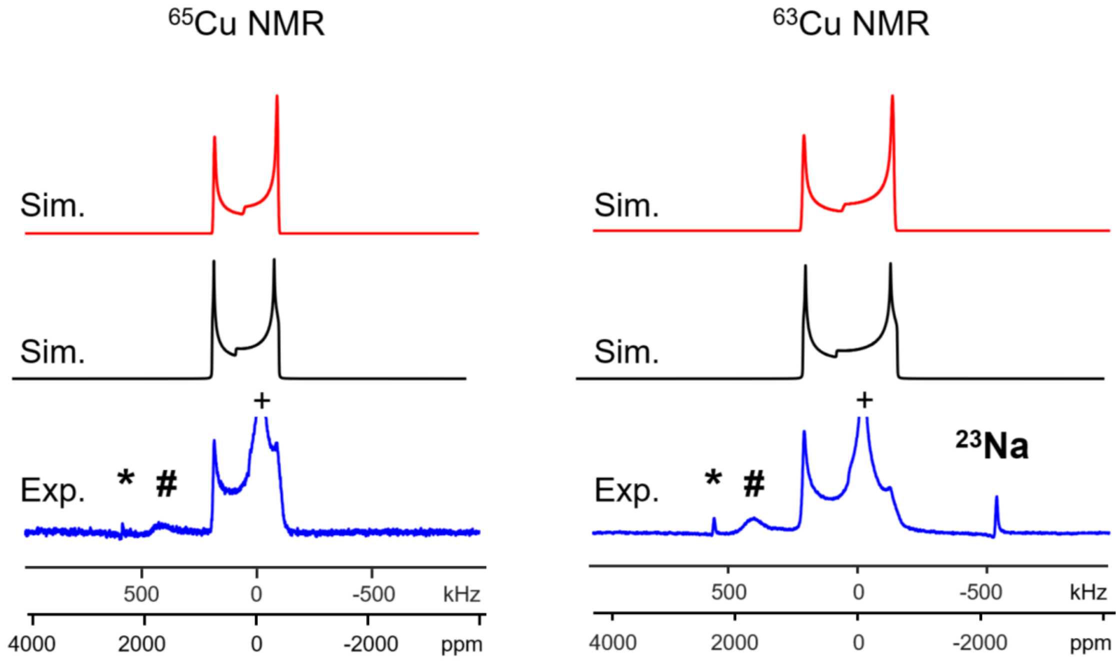

Figure S8. The [65]Cu (left) and [63]Cu (right) static solid echo NMR spectra of $\{[Cu(I)][Cu(II)(pdc)(H_2O)]\cdot 1.5MeCN\cdot H_2O\}_n$ at 21.1 T are shown in blue. The simulated spectra are shown at top, where the red trace includes CSA and the black trace excludes CSA. The asterisk (*) denotes a signal from metallic copper (Cu(0)), the pound (#) marks the signal from probe background, the plus (+) signifies a truncated signal associated with CuI impurities from residual reagent, and [23]Na marks interference from a background sodium signal.

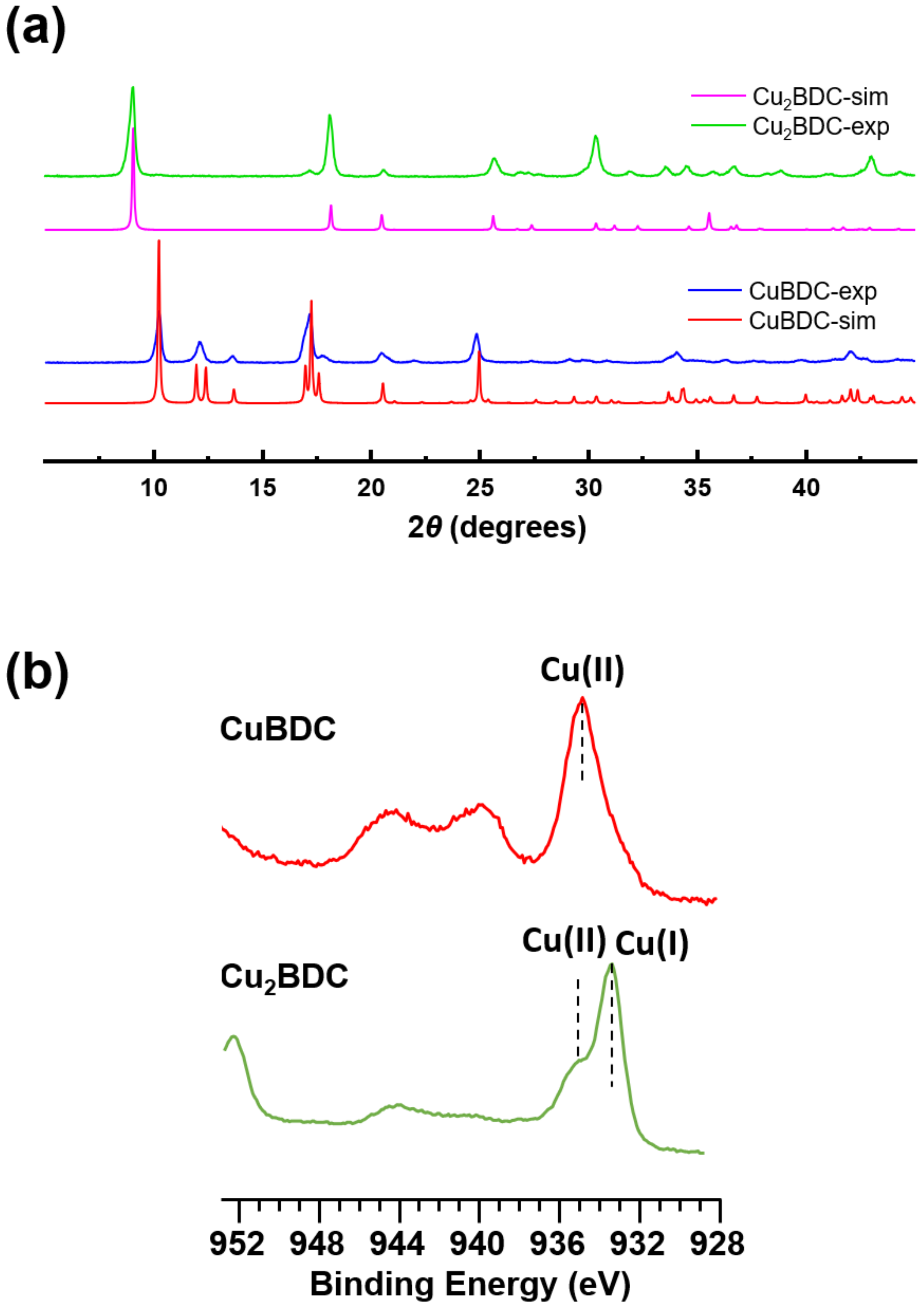

Figure S9. (a) The experimental (exp) and simulated (sim) PXRD patterns of CuBDC and Cu_2BDC. (b) The Cu $2p_{3/2}$ XPS spectra of CuBDC and Cu_2BDC. In the parent CuBDC MOF, the Cu(II) ions give rise to strong satellite signals in the region between 946 and 938 eV. After post-synthetic modification to Cu_2BDC and reduction of half the Cu(II) to Cu(I), the intensity of satellite Cu(II) signals in the XPS spectrum was significantly reduced, indicating a Cu(II) reduction to Cu(I) had occurred.

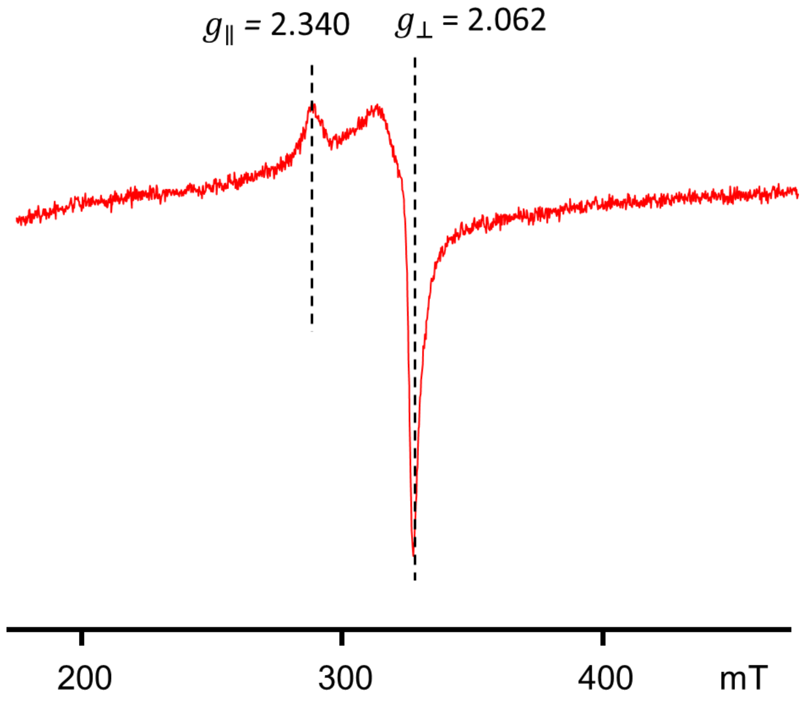

Figure S10. The X-band EPR spectrum of Cu_2BDC at 298 K.

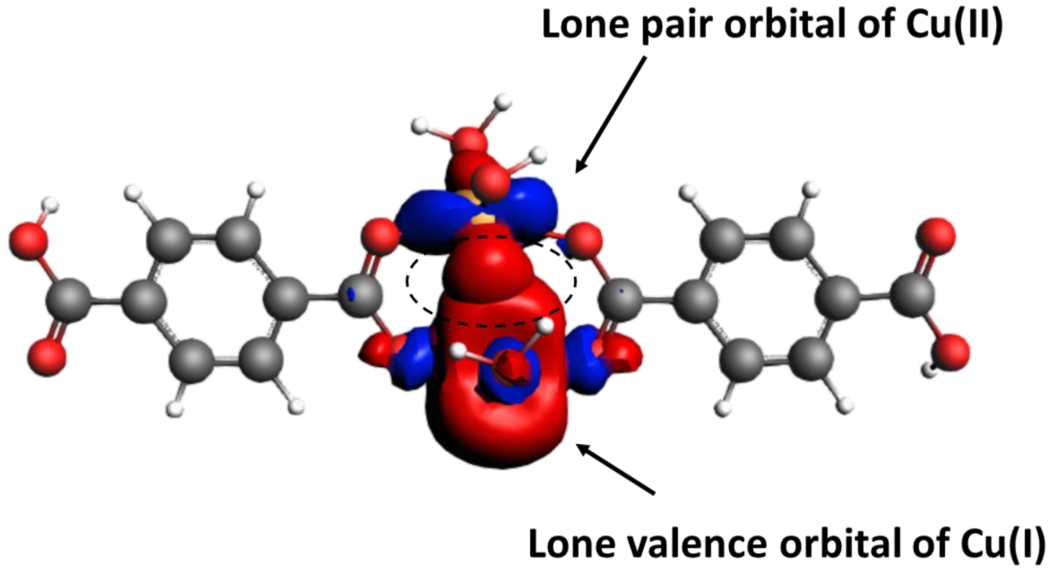

Figure S11. An illustration of the localized molecular orbital[31] overlap between the Cu(I) and Cu(II) metal centers in the Cu_2BDC MOF, which was obtained from DFT calculations performed using the ADF software package with general GGA, revPBE, and the TZ2P basis set. The specific location of maximum overlap is denoted by the black dashed line circle. The molecular orbital overlap demonstrates how the Cu(II) unpaired electrons may be delocalized into regions proximate to the diamagnetic Cu(I) center, influencing $^{63/65}$Cu NMR spectra and Cu CSA parameters.

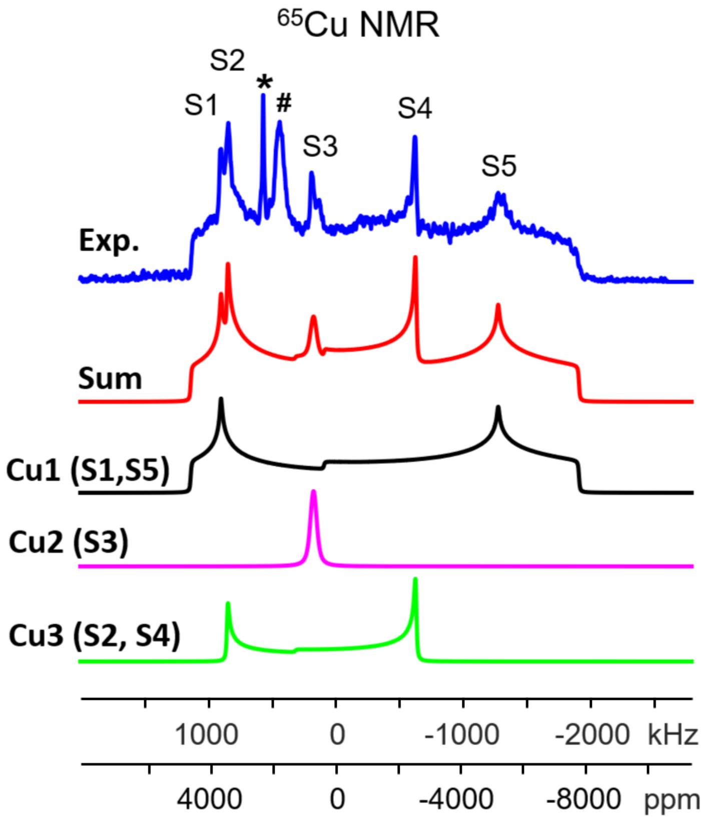

Figure S12. Simulations of the blue experimental ^{65}Cu NMR spectrum of $Cu(bpy)_{1.5}NO_3 \cdot 1.25H_2O$ are shown. The red "sum" simulation incorporates the simulation of all individual Cu sites. Cu1 (black simulation) originates from the Cu(I) site in the MOF, while the Cu2 (purple simulation) and Cu3 (green simulation) signals arise from side products. The S1 and S5 features are due to Cu1, while the S2, S3, and S4 features come about from Cu2 and Cu3 of the side products.

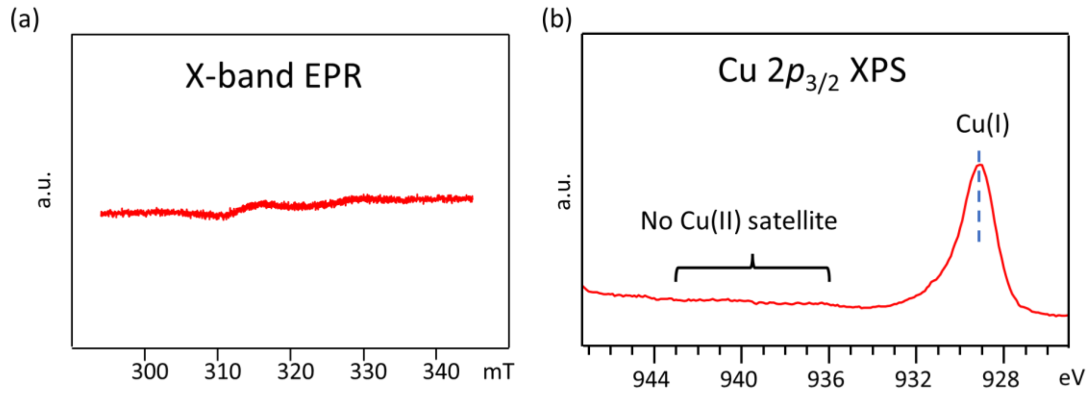

Figure S13. The X-band EPR spectrum at 298 K of SLUG-22 is shown in (a), along with the Cu $2p_{3/2}$ XPS spectrum of SLUG-22 in (b). Note the lack of Cu(II) satellites in (b).

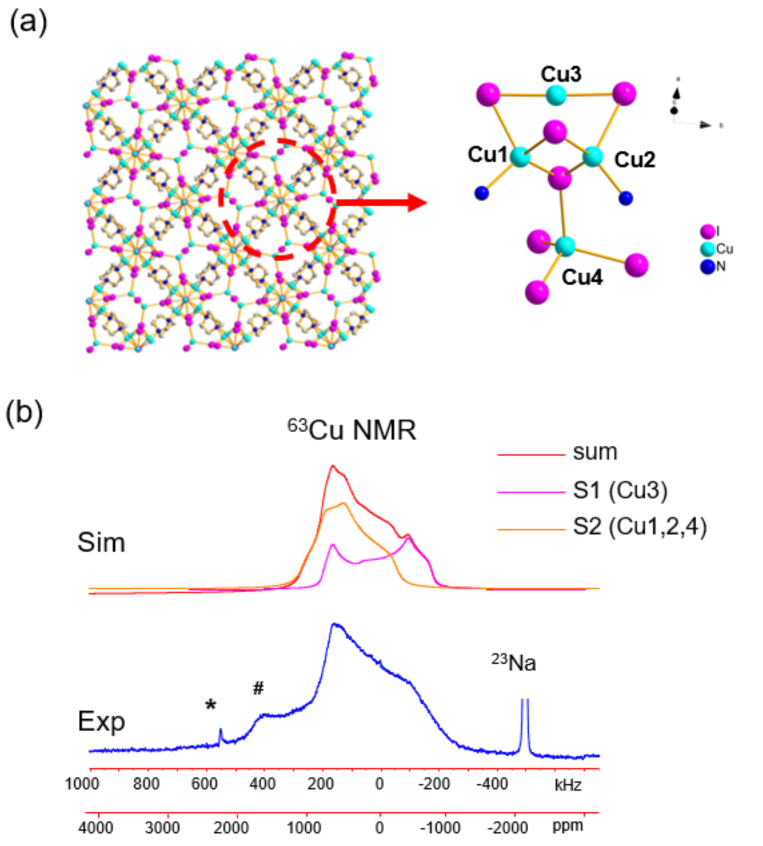

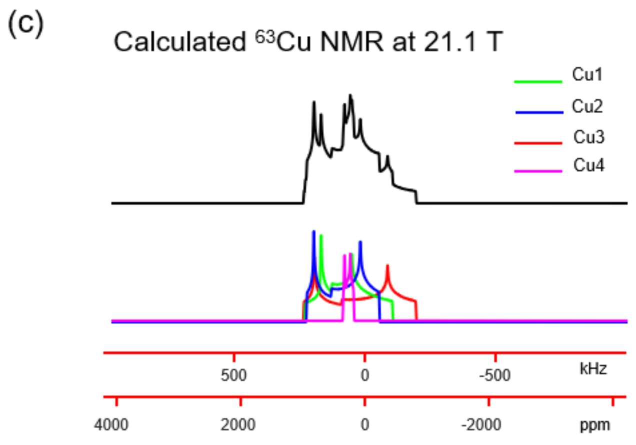

Calculated ^{63}Cu NMR parameters

Site	δ_{iso}(ppm)	$\lvert C_Q \rvert$(MHz)	η_Q
Cu3	446.1	25.7	0.24
Cu1	577.6	20.4	0.54
Cu2	622.8	20.6	0.25
Cu4	377.1	7.8	0.34

Figure S14. (a) A schematic illustration of the long- and short-range structure within [Cu_6I_6(DABCO)$_2$], along with (b) the experimental (blue) and simulated (red, with individual site contributions in orange and purple) ^{63}Cu static solid echo NMR spectra of [Cu_6I_6(DABCO)$_2$] at 21.1 T. The asterisk (*) denotes a signal from metallic copper (Cu(0)), the pound (#) marks the signal from probe background, and a ^{23}Na resonance from the probe background is also shown but truncated in intensity. (c) A simulated ^{63}Cu NMR spectrum at 21.1 T, constructed using the DFT-calculated NMR parameters. The calculated ^{63}Cu NMR parameters are included below (c), at bottom right.

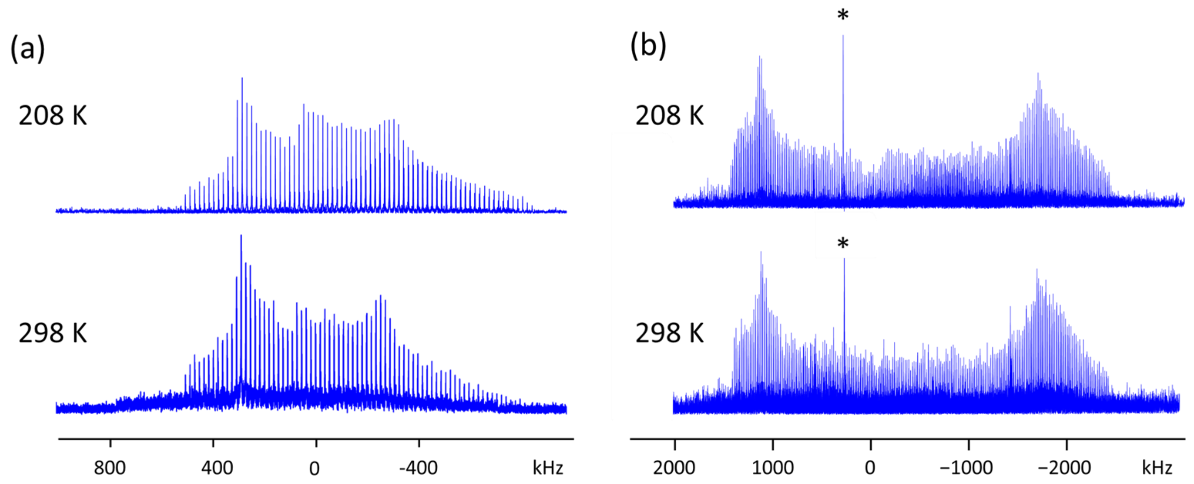

Figure S15. The static [65]Cu NMR spectra of (a) [CuI(bpy)] and (b) Cu_2BDC are shown at 9.4 T, as acquired at temperatures of 298 K and 208 K. The asterisk (*) denotes a signal from metallic copper (Cu(0)). Note that there are very little temperature-related differences in both instances.

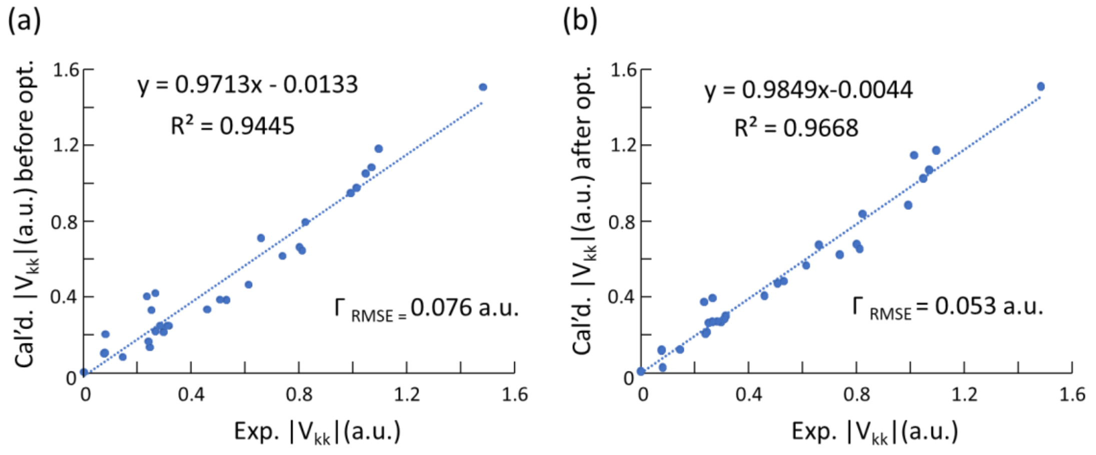

Figure S16. The relationship between the calculated and experimental principal [63/65]Cu EFG tensor components ($|V_{kk}|$, k = 1,2,3) of small complexes is plotted, in the situation when EFG calculations were performed (a) before and (b) after geometry optimization. Note the stronger correlation (*i.e.*, higher R^2) after geometry optimization. All calculations were performed in this study, with the experimental results taken from prior reports.

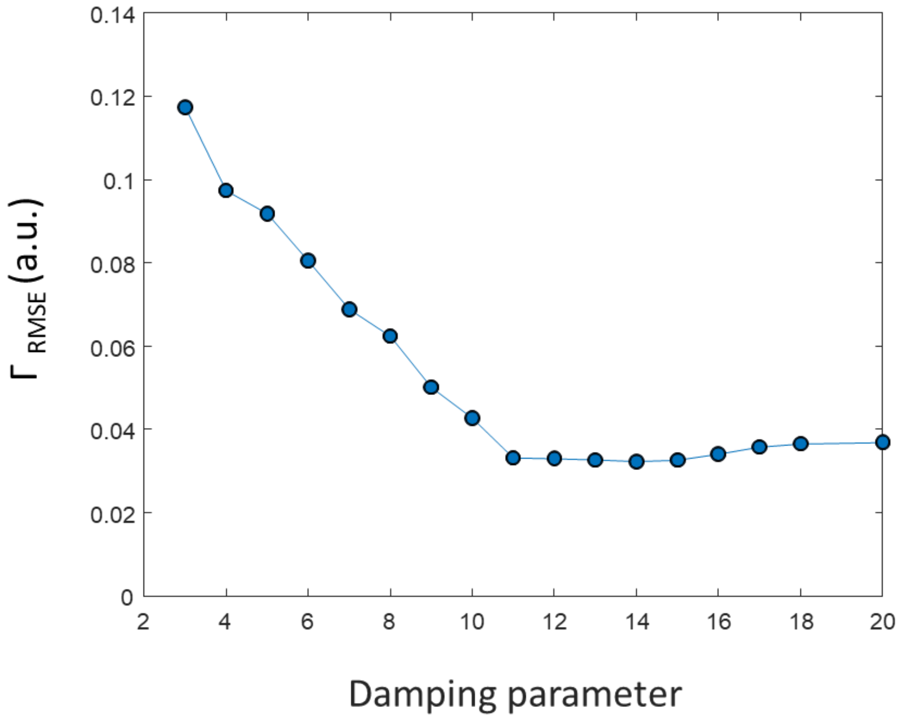

Figure S17. The root mean square EFG distances (Γ_{RMSE}) between the experimental and calculated principal components of the Cu EFG tensors corresponding to the Cu(I) complexes in Table S7, plotted as a function of the damping parameter. There is no obvious minimum between a damping factor (*d*) of 12-20, so a damping factor of 14 was adopted.

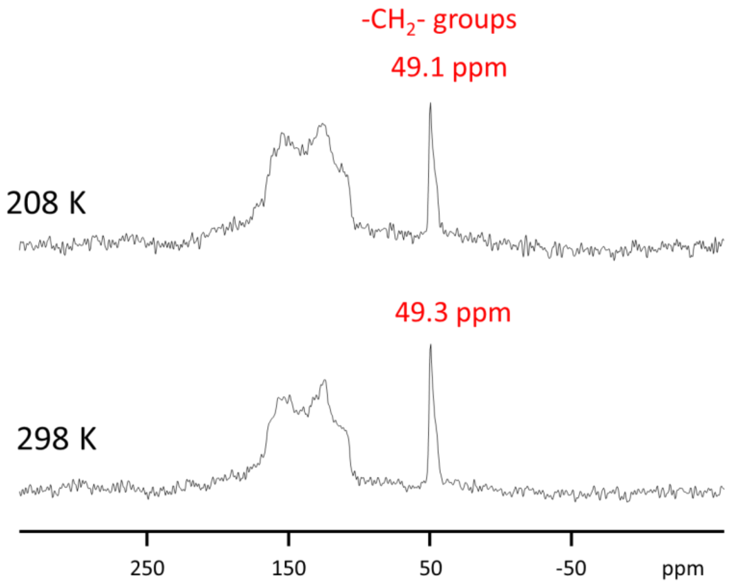

Figure S18. The ^{1}H-^{13}C CP/MAS NMR spectra of SLUG-22, as acquired at 9.4 T and temperatures of 298 K and 208 K as indicated; both spectra were obtained using a spinning rate of 14 kHz. Note that there are 20 inequivalent carbons in the SLUG-22 linkers, which resonate in the ca. 100 – 200 ppm range.

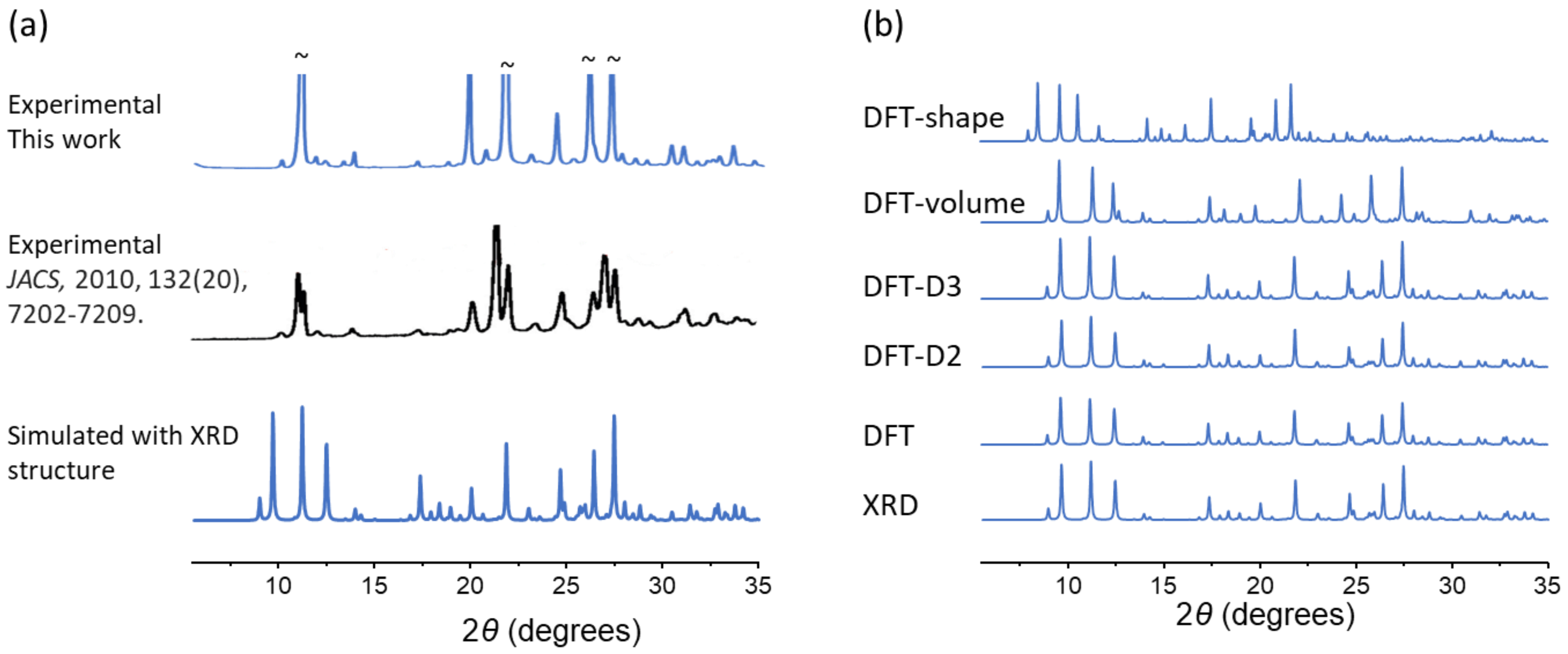

Figure S19. (a) The simulated and experimental XRD patterns of SLUG-22 from this work and previous accounts.[6] Note that the previously reported experimental PXRD data in (a) is not wholly consistent with the simulated XRD pattern generated from the single crystal structure described in the same paper, which raises questions regarding the accuracy of the reported structure. In (a), very intense reflections have been truncated for clarity and denoted by the ~ character. (b) The simulated PXRD patterns of SLUG-22 are shown, as generated from the geometry optimized structures of the single crystal structure using the indicated DFT method.

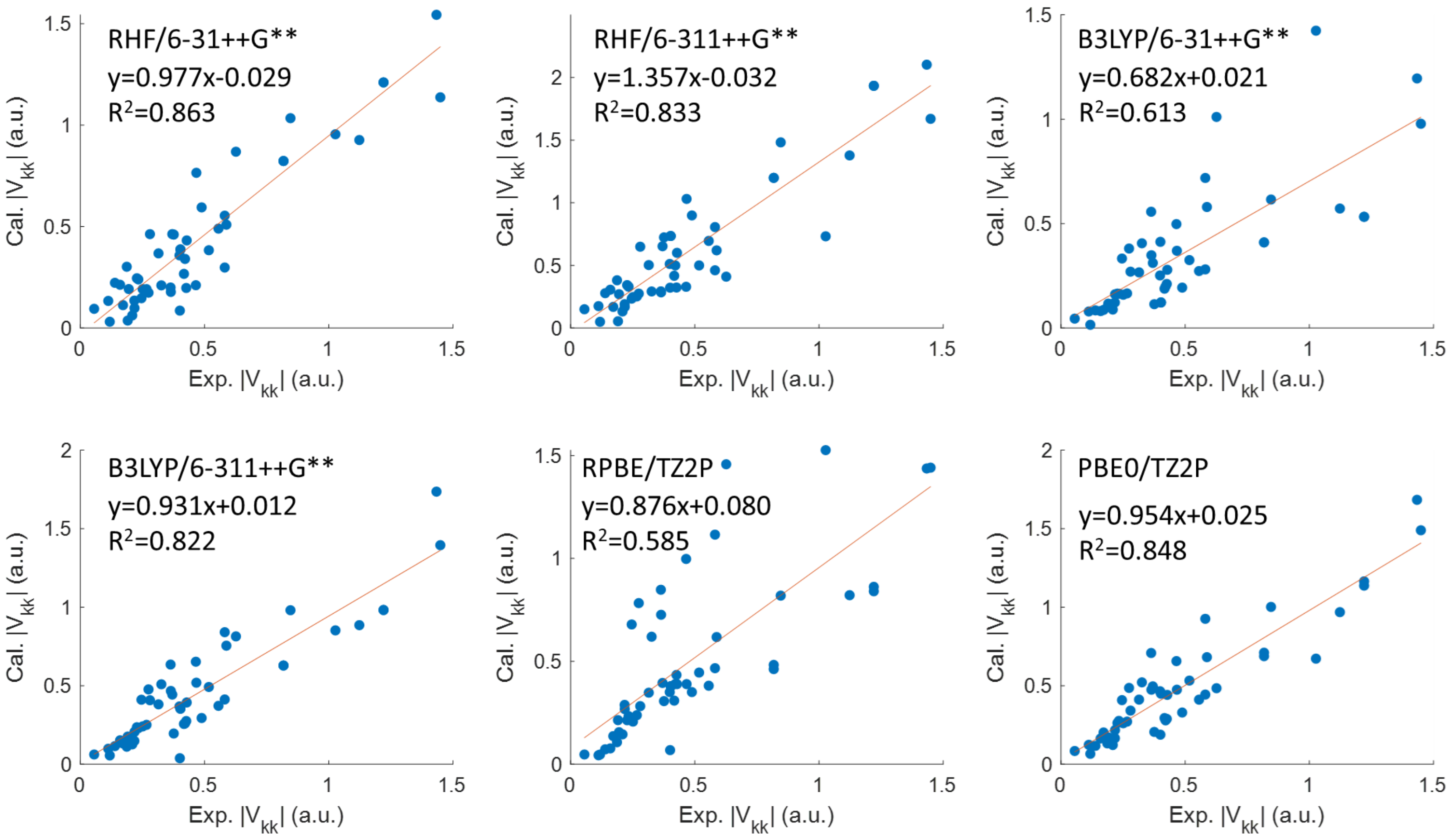

Figure S20. A series of comparison charts plotting the calculated EFG tensor components obtained using DFT cluster calculations versus the experimentally determined EFG tensor components.

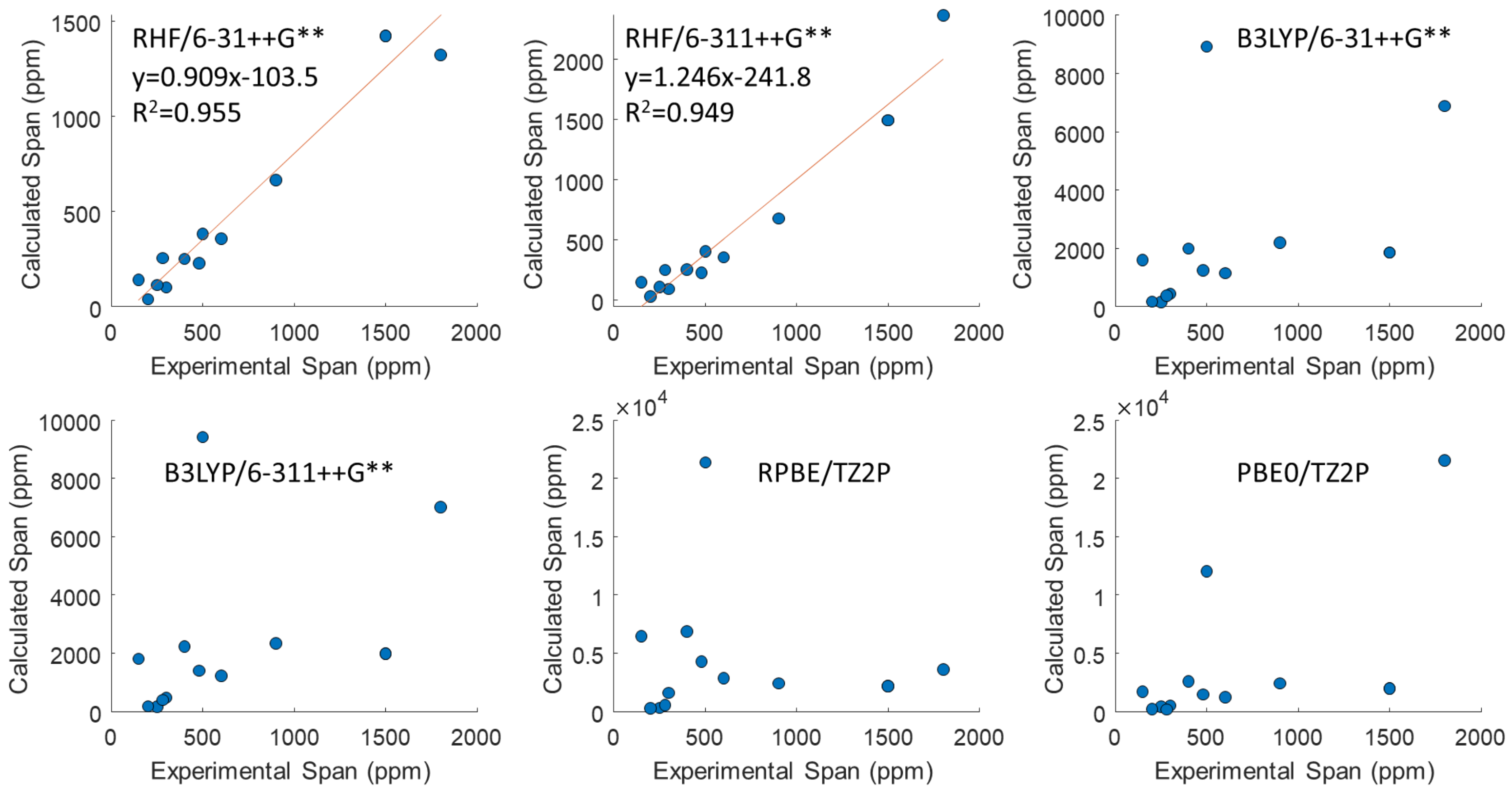

Figure S21. The experimentally obtained Cu MOF CS span values plotted against the span values calculated using a DFT cluster approach. The calculated span values were obtained using different methods and basis sets, as indicated at top of each graph.

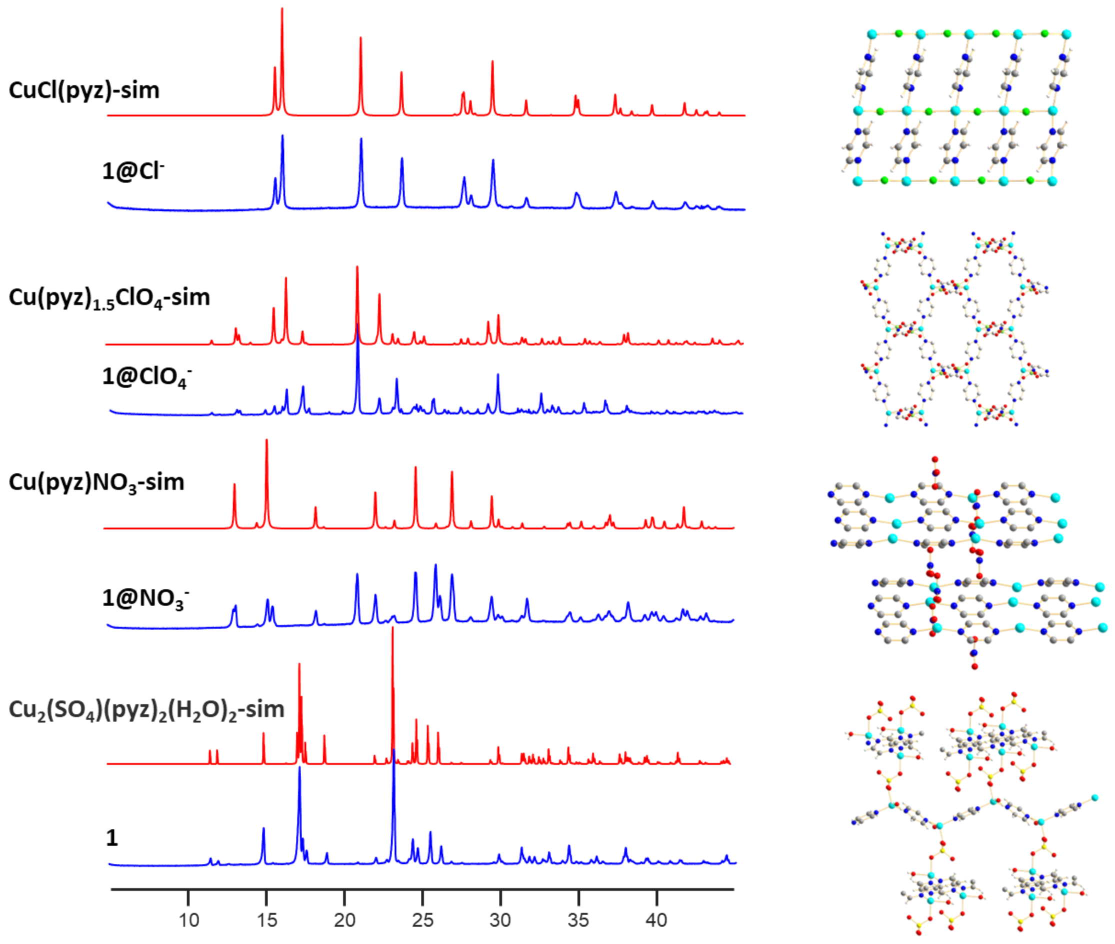

Figure S22. The experimental (blue) PXRD patterns of $Cu_2(SO_4)(pyz)_2(H_2O)_2$ (termed **1**) before and after anion exchange are shown, where the anion exchange products are denoted as **1**@NO_3^-, **1**@ClO_4^-, and **1**@Cl^-. The calculated (red) PXRD patterns of $Cu(pyz)NO_3$, $Cu(pyz)_{1.5}ClO_4$, and CuCl(pyz) formed from solvothermal methods instead of an anion exchange approach are also provided. Note that the experimental and simulated XRD patterns do not completely match for **1**@NO_3^- and **1**@ClO_4^-, indicating that these anion exchange products are not the exact same phase obtained from solvothermal methods.

Appendix A: The calculated Cu EFG parameters of small molecules from prior reports.

Table S7. Previously reported experimental $C_Q(^{65}Cu)$ and η_Q values, along with our corresponding DFT calculated values, for several molecular Cu(I) complexes.

**Compound**	**Experimental[a]**		**Calculated without geometry optimization**		**Calculated with geometry optimization**	
	$C_Q(^{65}Cu)$(MHz)	η_Q	$C_Q(^{65}Cu)$ (MHz)	η_Q	$C_Q(^{65}Cu)$ (MHz)	η_Q
$[Cu(PhCN)_4]BF_4$	3.6	0.95	5.2	0.90	5.7	0.94
$CpCuPPh_3$	29.4	0.03	22.3	0.07	27.1	0.06
$Cp^{+}CuPPh_3$	25.4	0.07	19.9	0.29	23.1	0.11
$Cp^{*}CuPPh_3$	24.3	0.05	18.8	0.01	22.5	0.08
$(hfac)CuPMe_3$	52.5	0.85	56.6	0.65	56.2	0.96
$[Me_3NN]Cu(CNAr)$	71.0	0.11	72.3	0.06	72.3	0.11
$[ClCuPPh_2Mes]_2$	51.2	0.50	52.0	0.22	51.2	0.27
$[BrCuPPh_2Mes]_2$	50.2	0.55	50.4	0.23	49.1	0.28
$C_{29}H_{29}ClCuN_4P$	19.2	0.46	12.4	0.87	12.6	0.74
$C_{29}H_{29}BrCuN_4P$	16.9	0.93	11.4	0.75	14.9	0.75
$[ICuPPh_3]_4$[b]	47.5	0.49	45.5	0.30	42.3	0.40
	22.0	0.36	16.0	0.49	19.4	0.41
$[ICuPPh_2Mes]_2$[c]	46.9	0.48	45.7	0.35	-	-
$[BrCuPPh_3]_4\cdot 2CHCl_3$[c,d]	51.0	0.39	49.0	0.25	-	-
	23.5	0.79	16.4	0.91	-	-

[a] The experimental EFG parameters were taken from ^{65}Cu NMR experiments at 21.1 T or 9.4 T by Tang et al.[32] and Yu et al.[33]

[b] The $[ICuPPh_3]_4$ complex has two unique Cu(I) sites. The top row, with the higher C_Q value, corresponds to the trigonal site. The bottom row describes the tetrahedral site.

[c] Geometry optimization of these two compounds was attempted, but both calculations failed to meet the convergence criteria.

[d] The $[BrCuPPh_3]_4\cdot 2CHCl_3$ complex has two unique Cu(I) sites. The top row, with the higher C_Q value, corresponds to the trigonal site. The bottom row describes the tetrahedral site.

Appendix B: Discussion of the EFG tensor orientations.

In the $CuNX_3$ and CuN_2X_2 (X = halide) tetrahedral configurations with N and halide donors within this study, the V_{33} component of the EFG tensor tends to point in the general direction of the N donor (Figure S23). This signifies that any changes in the Cu-N bond distances or ∠N-Cu-X will have a profound effect on the C_Q($^{63/65}$Cu) value, since C_Q is directly proportional to V_{33}. In addition, the donating ability of the N-based linker, and by consequence the identity of the N-based linker itself, will influence C_Q. In a three-coordinate trigonal Cu(I) environment with three N donors, V_{33} is aligned nearly perpendicular to the bonding plane, and any interactions involving Cu(I) in this direction will affect C_Q($^{63/65}$Cu). In a two-coordinate linear arrangement with two Cu-N bonds, V_{33} tends be oriented near the Cu-N bonds, with the ∠N-Cu-N angle and Cu-N bond distances having a direct impact on C_Q. The identity of the N-based linker and its donor strength will thus significantly influence C_Q.

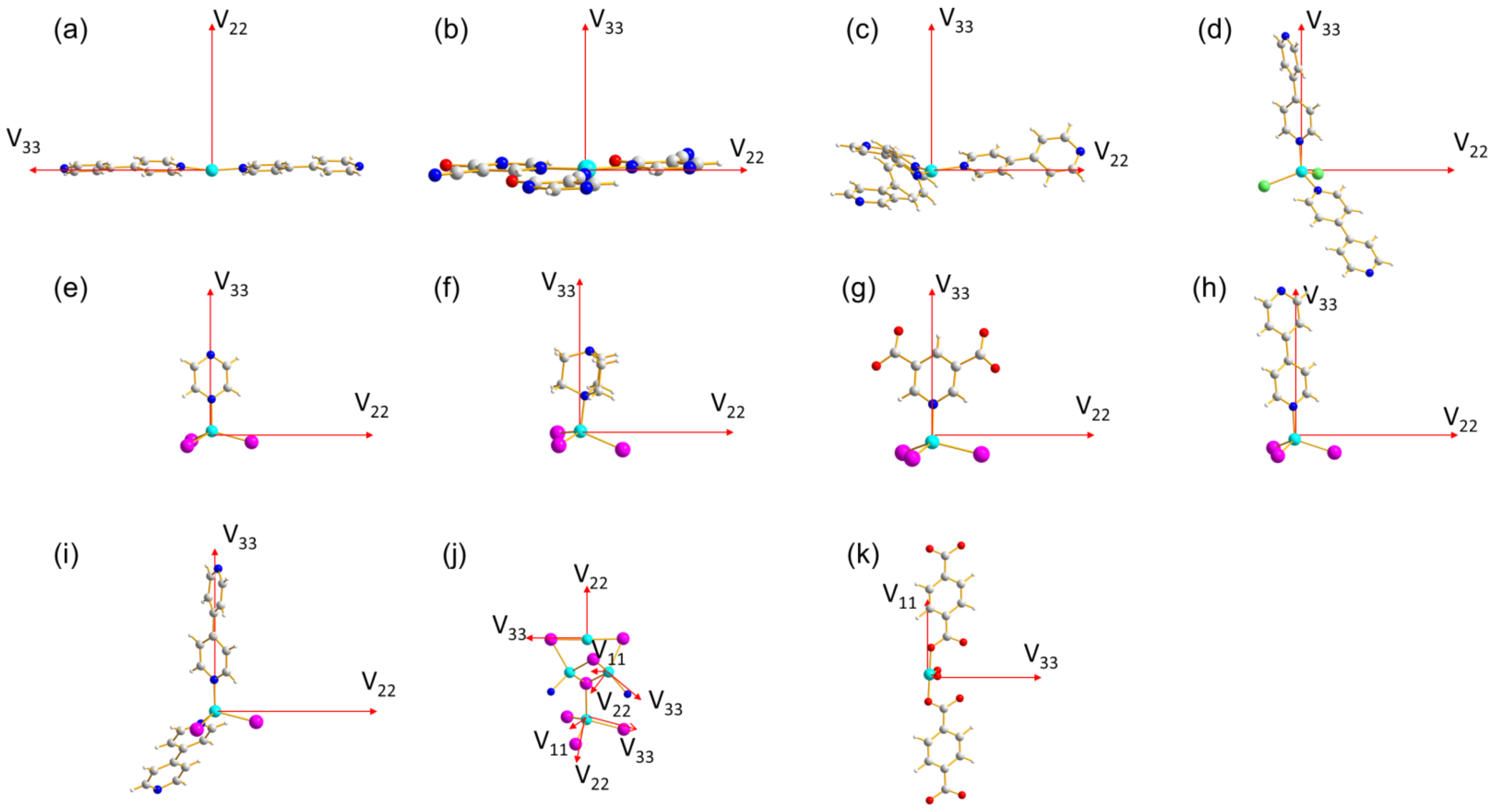

Figure S23. Visual representations of the $^{63/65}$Cu EFG tensors calculated from plane-wave DFT calculations for (a) SLUG-22, (b) $Cu_3(4hypymca)_3$, (c) Cu(bpy)$1.5NO_3 \cdot 1.25H_2O$, (d) [CuCl(bpy)], (e) [Cu_2I_2(pyz)], (f) [Cu_4I_4(DABCO)], (g) {[CuI][Cu(pdc)(H_2O)]·1.5MeCN·H_2O}$_n$, (h) [Cu_2I_2(bpy)], (i) [CuI(bpy)], (j) [Cu_6I_6(DABCO)], and (k) Cu_2BDC. In all instances, the tensor component not explicitly labeled (V_{11}, V_{22}, or V_{33}) is directed perpendicular to the plane of the page and through the Cu center.

Appendix C: Correlation plots of EFG and CSA from plane-wave DFT (CASTEP) and cluster calculations

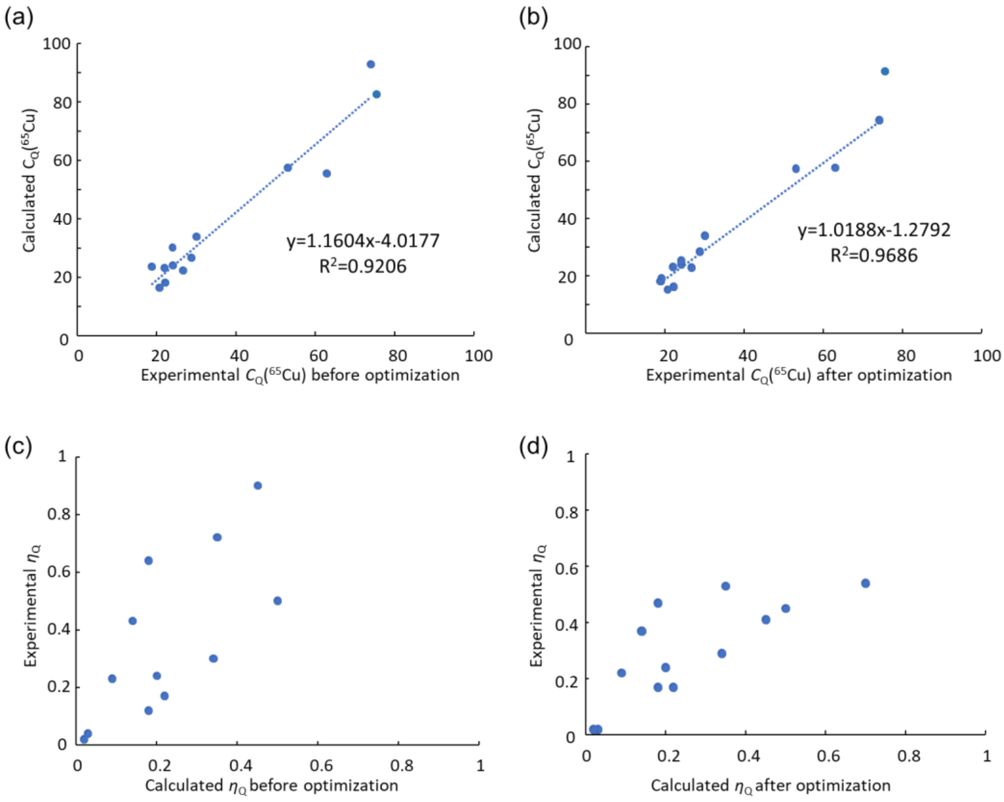

Figure S24. A plot of the calculated versus experimental ^{65}Cu EFG tensor parameters in MOFs without (a,c) and with (b,d) geometry optimization being performed prior to using CASTEP plane-wave DFT calculations.

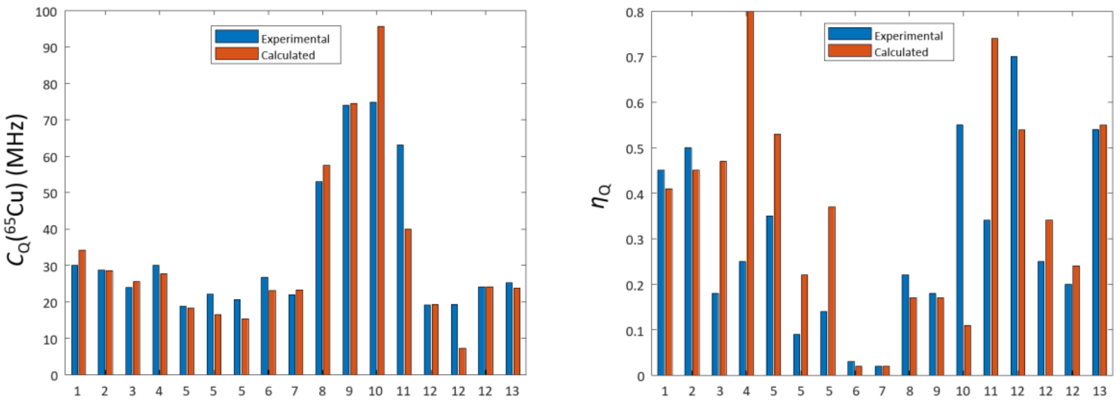

1:[CuCl(bpy)] 4:[Cu_2I_2(pyz)] 7:[Cu_2Cl_2(bpy)] 8:Cu_2BDC 11:SLUG-22
2:[CuI(bpy)] 5:[Cu_4I_4(DABCO)] 9:Cu(bpy)1.5NO_3·1.25H_2O 12:[Cu_6I_6(DABCO)]
3:[Cu_2I_2(bpy)] 6:[CuIpdc] 10:Cu_3(4hy-pymca)$_3$ 13:[Cu_2(pyz)$_2$(SO_4)(H_2O)$_2$

Figure S25. A plot of the calculated versus experimental ^{65}Cu EFG tensor parameters in MOFs after geometry optimization with plane-wave DFT calculations.

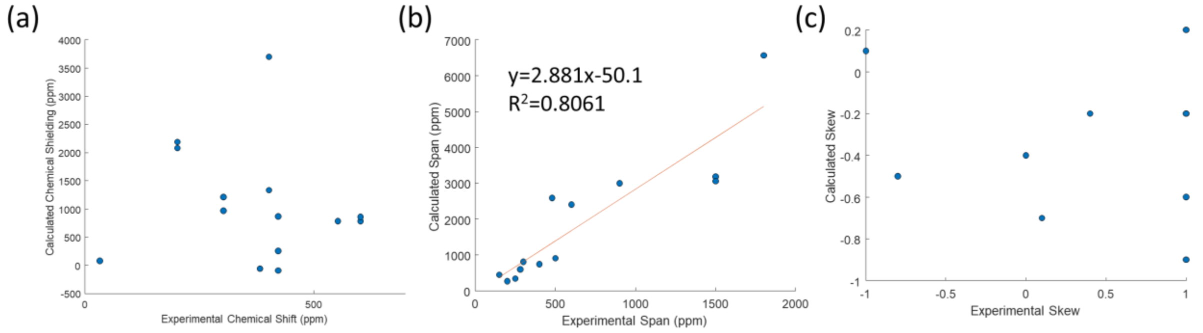

Figure S26. The experimental Cu MOF CS tensor parameters are shown, as compared to those obtained using CASTEP plane-wave DFT calculations.

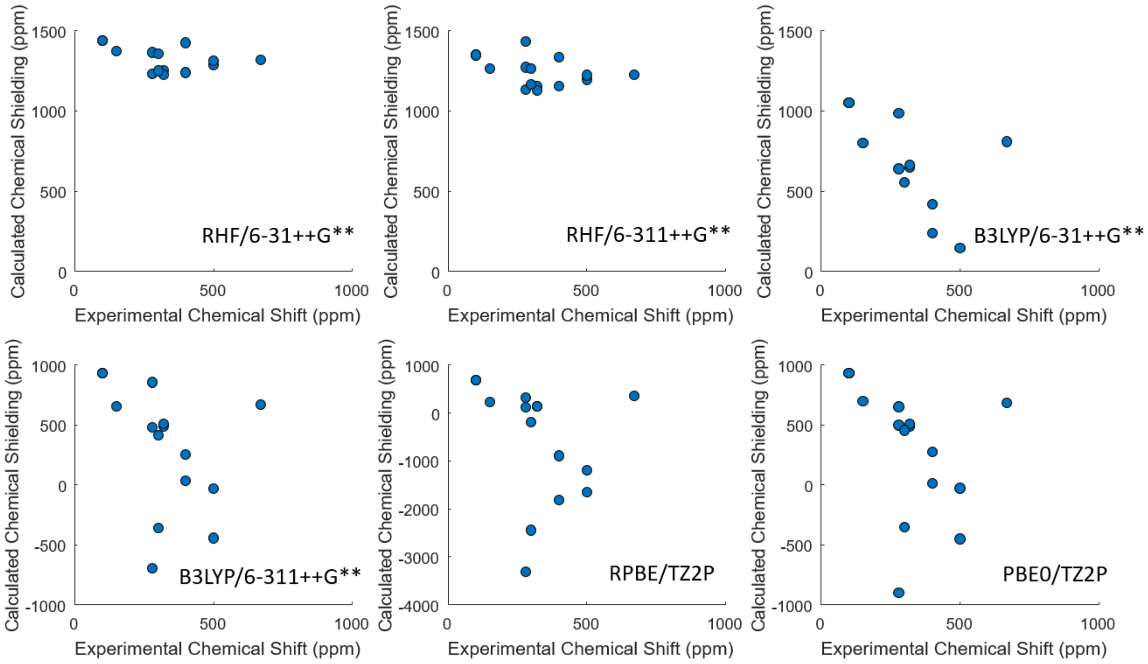

Figure S27. A comparison of experimentally measured Cu isotropic chemical shifts in MOFs versus the calculated isotropic chemical shieldings obtained using a DFT cluster strategy, with the method and basis set indicated at bottom right of each graph.

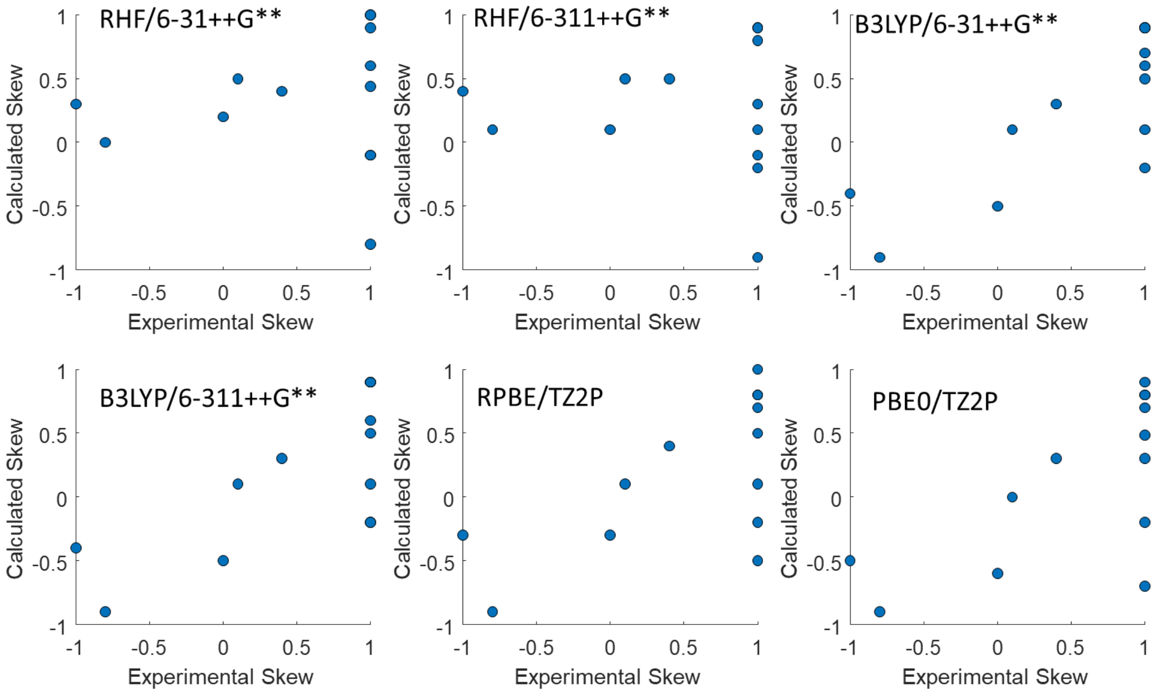

Figure S28. The experimentally determined Cu MOF CS skew values compared to those calculated using different methods and basis sets in a DFT cluster approach, with calculation conditions indicated at top left of every graph.

References

1 O. M. Yaghi and G. Li, *Angew. Chemie Int. Ed.*, 1995, **34**, 207–209.

2 S. R. Batten, J. C. Jeffery and M. D. Ward, *Inorganica Chim. Acta*, 1999, **292**, 231–237.

3 C. Näther and I. Jeß, *Monatshefte für Chemie/Chemical Mon.*, 2001, **132**, 897–910.

4 D. Braga, L. Maini, P. P. Mazzeo and B. Ventura, *Chem. Eur. J.*, 2010, **16**, 1553–1559.

5 Y. Zhang, T. Wu, R. Liu, T. Dou, X. Bu and P. Feng, *Cryst. Growth Des.*, 2010, **10**, 2047–2049.

6 H. Fei, D. L. Rogow and S. R. J. Oliver, *J. Am. Chem. Soc.*, 2010, **132**, 7202–7209.

7 O. M. Yaghi and H. Li, *J. Am. Chem. Soc.*, 1995, **117**, 10401–10402.

8 A. A. García-Valdivia, F. J. Romero, J. Cepeda, D. P. Morales, N. Casati, A. J. Mota, L. A. Zotti, J. J. Palacios, D. Choquesillo-Lazarte and J. F. Salmerón, *Chem. Commun.*, 2020, **56**, 9473–9476.

9 X. Zhou, J. Dong, Y. Zhu, L. Liu, Y. Jiao, H. Li, Y. Han, K. Davey, Q. Xu and Y. Zheng, *J. Am. Chem. Soc.*, 2021, **143**, 6681–6690.

10 S. Demir, H. M. Çepni, N. Bilgin, M. Hołyńska and F. Yilmaz, *Polyhedron*, 2016, **115**,

236–241.

11 L. A. O'Dell and R. W. Schurko, *Chem. Phys. Lett.*, 2008, **464**, 97–102.

12 D. Massiot, I. Farnan, N. Gautier, D. Trumeau, A. Trokiner and J. P. Coutures, *Solid State Nucl. Magn. Reson.*, 1995, **4**, 241–248.

13 S. Sturniolo, T. F. G. Green, R. M. Hanson, M. Zilka, K. Refson, P. Hodgkinson, S. P. Brown and J. R. Yates, *Solid State Nucl. Magn. Reson.*, 2016, **78**, 64–70.

14 S. Grimme, S. Ehrlich and L. Goerigk, *J. Comput. Chem.*, 2011, **32**, 1456–1465.

15 S. T. Holmes, C. S. Vojvodin and R. W. Schurko, *J. Phys. Chem. A*, 2020, **124**, 10312–10323.

16 S. T. Holmes and R. W. Schurko, *J. Phys. Chem. C*, 2018, **122**, 1809–1820.

17 M. J. Frisch, G. W. Trucks, H. B. Schlegel, G. E. Scuseria, M. A. Robb, J. R. Cheeseman, G. Scalmani, V. Barone, G. A. Petersson and H. H. Nakatsuji, *Gaussian 16*, 2016. M. J. Frisch, G. W. Trucks, H. B. Schlegel, G. E. Scuseria, M. A. Robb, J. R. Cheeseman, G. Scalmani, V. Barone, G. A. Petersson, H. Nakatsuji, X. Li, M. Caricato, A. V. Marenich, J. Bloino, B. G. Janesko, R. Gomperts, B. Mennucci, H. P. Hratchian, J. V. Ortiz, A. F. Izmaylov, J. L. Sonnenberg, D. Williams-Young, F. Ding, F. Lipparini, F. Egidi, J. Goings, B. Peng, A. Petrone, T. Henderson, D. Ranasinghe, V. G. Zakrzewski, J. Gao, N. Rega, G. Zheng, W. Liang, M. Hada, M. Ehara, K. Toyota, R. Fukuda, J. Hasegawa, M. Ishida, T. Nakajima, Y. Honda, O. Kitao, H. Nakai, T. Vreven, K. Throssell, J. A. Montgomery Jr, J. E. Peralta, F. Ogliaro, M. J. Bearpark, J. J. Heyd, E. N. Brothers, K. N. Kudin, V. N. Staroverov, T. A. Keith, R. Kobayashi, J. Normand, K. Raghavachari, A. P. Rendell, J. C. Burant, S. S. Iyengar, J. Tomasi, M. Cossi, J. M. Millam, M. Klene, C. Adamo, R. Cammi, J. W. Ochterski, R. L. Martin, K. Morokuma, O. Farkas, J. B. Foresman and D. J. Fox, *Gaussian* 16, *Revision C*, Gaussian, Inc., Wallingford CT, 2016.

18 P. J. Stephens, F. J. Devlin, C. F. Chabalowski and M. J. Frisch, *J. Phys. Chem.*, 1994, **98**, 11623–11627.

19 A. D. Becke, *J. Chem. Phys*, 1993, **98**, 5648–5652.

20 M. N. Glukhovtsev, A. Pross, M. P. McGrath and L. Radom, *J. Chem. Phys.*, 1995, **103**, 1878–1885.

21 G. Te Velde, F. M. Bickelhaupt, E. J. Baerends, C. Fonseca Guerra, S. J. A. van

Gisbergen, J. G. Snijders and T. Ziegler, *J. Comput. Chem.*, 2001, **22**, 931–967.

22 S. Grimme, *J. Comput. Chem.*, 2004, **25**, 1463–1473.

23 M. Ernzerhof and G. E. Scuseria, *J. Chem. Phys.*, 1999, **110**, 5029–5036.

24 E. D. Glendening, C. R. Landis and F. Weinhold, *J. Comput. Chem.*, 2013, **34**, 1429–1437.

25 S. Adiga, D. Aebi and D. L. Bryce, *Can. J. Chem.*, 2007, **85**, 496–505.

26 C. Cappuccino, F. Farinella, D. Braga and L. Maini, *Cryst. Growth Des.*, 2019, **19**, 4395–4403.

27 N. V. S. Harisomayajula, S. Makovetskyi and Y. Tsai, *Chem. Eur. J.*, 2019, **25**, 8936–8954.

28 N. Kuganathan and J. C. Green, *Chem. Commun.*, 2008, 2432–2434.

29 M. A. Kinzhalov, D. M. Ivanov, A. V Shishkina, A. A. Melekhova, V. V Suslonov, A. Frontera, V. Y. Kukushkin and N. A. Bokach, *Inorg. Chem. Front.*, 2023, **10**, 1522–1533.

30 P. Pyykkö, *Mol. Phys.*, 2018, **116**, 1328–1338.

31 A. E. Reed and F. Weinhold, *J. Chem. Phys.*, 1985, **83**, 1736–1740.

32 J. A. Tang, B. D. Ellis, T. H. Warren, J. V Hanna, C. L. B. Macdonald and R. W. Schurko, *J. Am. Chem. Soc.*, 2007, **129**, 13049–13065.

33 H. Yu, X. Tan, G. M. Bernard, V. V Terskikh, J. Chen and R. E. Wasylishen, *J. Phys. Chem. A*, 2015, **119**, 8279–8293.
